# Medicinal Chemistry of Small-Molecule c-Met Inhibitors: From Approved Therapies to Emerging Multitarget Anticancer Agents

**DOI:** 10.3390/ijms27188007

**Published:** 2026-09-09

**Authors:** Siva S. Panda, Mohamed S. Bekheit, Dalia R. Aboshouk, Sudhan Sivakumar, Mohamed A. Morsy, Mariam Abdur-Rahman, Abdelgawad Fahmi, Adel S. Girgis

**Affiliations:** 1 Department of Chemistry and Biochemistry, Augusta University, Augusta, GA 30912, USA; ssivakumar@augusta.edu; 2 Department of Biochemistry and Molecular Biology, Augusta University, Augusta, GA 30912, USA; 3 Department of Pesticide Chemistry, National Research Centre, Dokki, Giza 12622, Egypt; m_bekheit@yahoo.com (M.S.B.); daliaraslan205@yahoo.com (D.R.A.); 4 Al-Azhar Virology Research Center, Faculty of Medicine, Al-Azhar University, Cairo 71524, Egypt; mo.sci87@gmail.com; 5 Chemistry Department, Faculty of Science, Cairo University, Giza 12613, Egypt; mariam@sci.cu.edu.eg (M.A.-R.); abdelgawad5@hotmail.com (A.F.)

**Keywords:** c-Met, HGF/c-Met signaling, receptor tyrosine kinase, kinase inhibitors, medicinal chemistry, multitarget inhibitors, anticancer agents, drug discovery

## Abstract

The hepatocyte growth factor (HGF)/c-Met signaling pathway plays a central role in cellular proliferation, survival, migration, invasion, angiogenesis, and therapeutic resistance. Aberrant c-Met activation, driven by gene amplification, overexpression, activating mutations, exon 14-skipping alterations, or ligand-dependent stimulation, drives the development and progression of many solid tumors, positioning c-Met as a key target for anticancer drug development. The clinical effectiveness of c-Met-targeted treatments such as crizotinib, capmatinib, tepotinib, savolitinib, and cabozantinib has confirmed c-Met as a viable oncogenic driver for therapy, leading to the development of various next-generation inhibitors with different structures. This review provides a comprehensive perspective on small-molecule c-Met inhibitors from the perspectives of medicinal chemistry and structure-based drug design, encompassing approved drugs, investigational agents, natural-product-inspired leads, and emerging multitarget anticancer therapeutics. Particular emphasis is given to the principles of molecular recognition that govern c-Met inhibition. This includes the structure of the kinase domain, interactions at the ATP-binding site, recognition of the hinge region, and the different binding modes of Type I, Type II, and allosteric inhibitors. The design, synthesis, biological evaluation, and structure–activity relationships of diverse heterocyclic scaffolds that have shaped c-Met inhibitor discovery are critically analyzed. Key medicinal chemistry strategies, including scaffold hopping, bioisosteric replacement, conformational optimization, molecular hybridization, and multitarget pharmacophore integration, are discussed in the context of potency, selectivity, resistance management, and drug-like properties. Particular attention is given to the integration of structural biology, molecular docking, binding-mode analysis, and structure-guided optimization approaches that have enabled the development of potent c-Met-directed inhibitors. In addition, recent advances in dual- and multitarget agents that simultaneously modulate c-Met and complementary therapeutic targets, including VEGFR-2, EGFR, AXL, MER, PARP1, CDK2, and tubulin, are highlighted as promising strategies for overcoming pathway redundancy and acquired resistance. This review summarizes contemporary structure-based and medicinal chemistry principles underlying c-Met inhibitor discovery, critically evaluates the relationship between biochemical potency and therapeutic efficacy, and provides a framework for the rational design of next-generation c-Met-targeted and multitarget anticancer agents.

## 1. Introduction

Tyrosine kinases (TKs) are essential regulators of cellular signaling, controlling proliferation, survival, differentiation, migration, metabolism, and apoptosis by phosphorylating tyrosine residues on target proteins. By transferring the γ-phosphate group from adenosine triphosphate (ATP), TKs orchestrate signaling networks that maintain normal cellular homeostasis and coordinate cellular responses to extracellular stimuli [1,2]. Based on their structural organization and cellular localization, TKs are broadly classified as receptor tyrosine kinases (RTKs) or non-receptor tyrosine kinases. RTKs contain extracellular ligand-binding, transmembrane, and intracellular kinase domains, whereas non-receptor tyrosine kinases primarily function in the cytoplasm [3,4]. Among RTKs, the epidermal growth factor receptor (EGFR), vascular endothelial growth factor receptor (VEGFR), and cellular mesenchymal–epithelial transition factor (c-Met) have emerged as particularly important therapeutic targets because of their central roles in tumor growth, angiogenesis, metastasis, and therapeutic resistance [4,5].

Dysregulated tyrosine kinase signaling, driven by gene amplification, activating mutations, chromosomal rearrangements, ligand overproduction, or aberrant downstream pathway activation, is a hallmark of many human malignancies [5,6]. Consequently, kinase-directed therapies have become among the most successful classes of targeted anticancer agents. Advances in medicinal chemistry, structural biology, and molecular pharmacology have enabled the development of highly selective kinase inhibitors that have transformed precision oncology and improved outcomes for many patients with molecularly defined cancers [7,8].

Among these targets, c-Met has attracted considerable attention due to its diverse physiological functions and widespread involvement in cancer. c-Met is activated when hepatocyte growth factor (HGF) binds, triggering receptor dimerization, autophosphorylation of key tyrosine residues, and activation of downstream signaling pathways, including PI3K/AKT, RAS/MAPK, STAT3, SRC, and NF-κB [9,10]. Through these pathways, c-Met regulates proliferation, survival, migration, invasion, angiogenesis, epithelial–mesenchymal transition (EMT), tissue regeneration, and embryonic development. Aberrant c-Met signaling, driven by gene amplification, overexpression, activating mutations, exon 14-skipping alterations, or autocrine HGF stimulation, contributes to tumor progression, metastasis, therapeutic resistance, and poor prognosis in numerous cancers, including non-small-cell lung cancer, gastric cancer, hepatocellular carcinoma, colorectal cancer, pancreatic cancer, breast cancer, and renal cancer [11].

The biological and clinical importance of c-Met has led to the successful development of several c-Met-directed therapies. Agents such as crizotinib, capmatinib, tepotinib, savolitinib, and cabozantinib have demonstrated the therapeutic value of targeting c-Met-driven malignancies and have established c-Met inhibition as an effective precision-oncology strategy for selected patient populations. However, significant challenges still exist, including resistance due to kinase-domain mutations, bypass pathways, limited selectivity, off-target toxicities, and suboptimal pharmacokinetics. The need to maintain activity against tumors harboring c-Met exon 14-skipping alterations and acquired resistance mutations continues to drive the discovery of next-generation inhibitors with improved potency, selectivity, and resistance profiles. At the same time, more focus has been placed on dual- and multitarget inhibitors that can concurrently block c-Met and other related cancer pathways to counteract adaptive resistance.

Numerous reviews have examined c-Met biology, signaling, and clinical pharmacology [12,13,14,15,16,17,18,19]. However, relatively few have focused on the medicinal chemistry principles that underlie the discovery and optimization of small-molecule c-Met inhibitors. Moreover, rapid progress over the past five years has yielded a wide range of new heterocyclic scaffolds, scaffold-hopping strategies, and multitarget design approaches that have not been comprehensively integrated from a medicinal chemistry perspective.

This review critically summarizes advances in the medicinal chemistry of small-molecule c-Met inhibitors from 2021 to June 2026. A comprehensive literature search was conducted using key scientific databases, including PubMed, Scopus, and ScienceDirect, as well as the digital libraries of the American Chemical Society and the Royal Society of Chemistry. The review emphasizes heterocyclic scaffold design, structure–activity relationships, molecular recognition, kinase selectivity, resistance mechanisms, and emerging multitarget strategies. These developments offer valuable insights into the design of next-generation c-Met-targeted and multitarget anticancer agents.

## 2. Clinically Approved and Investigational Small-Molecule c-Met Inhibitors

**Figure 1:**
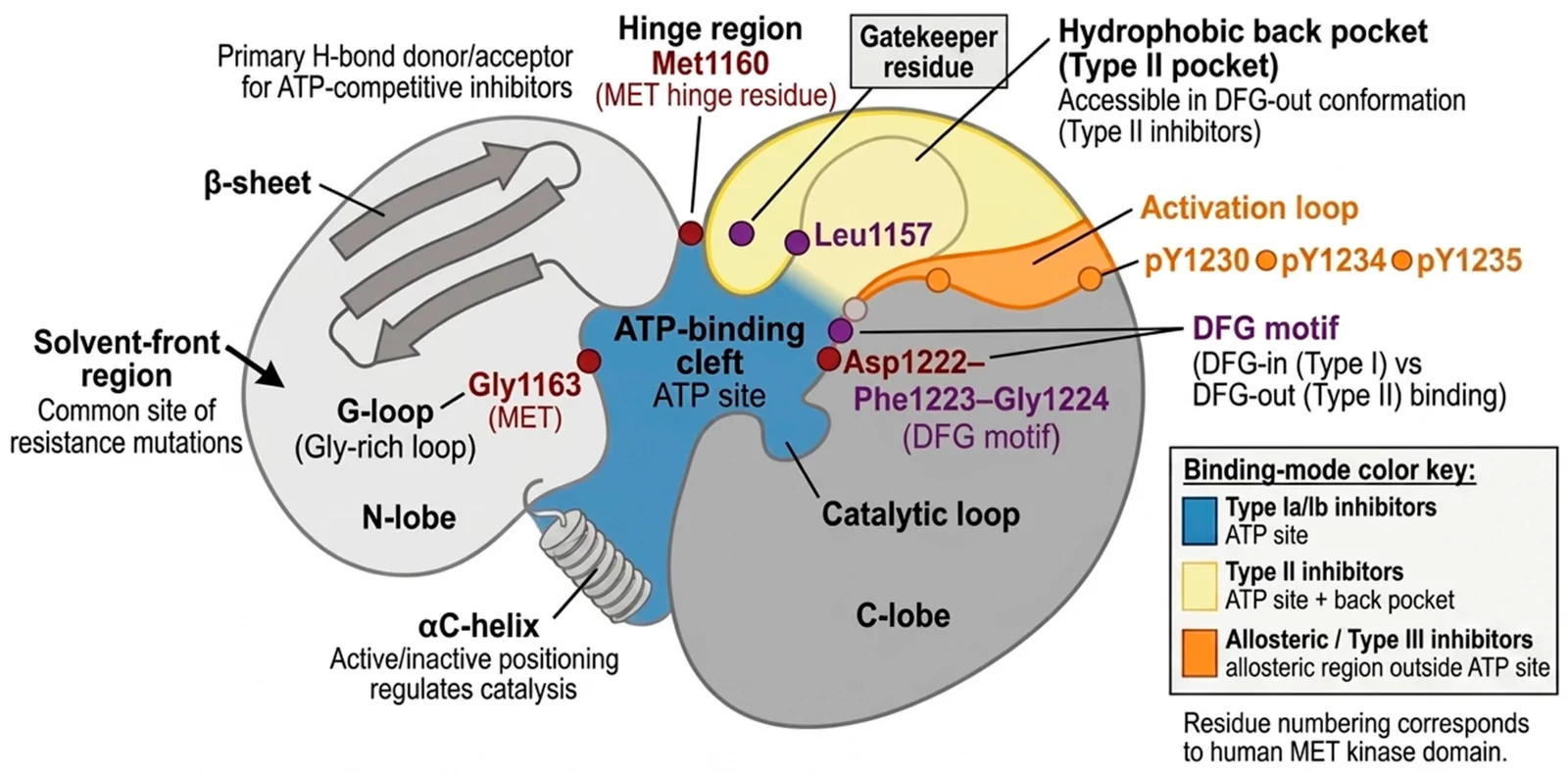
Kinase domain architecture and major kinase inhibitor binding modes. c-Met kinase domain architecture and principal inhibitor-binding regions. Schematic representation of the c-Met kinase domain showing the N-lobe, C-lobe, glycine-rich loop (G-loop), αC-helix, hinge region, ATP-binding cleft, catalytic loop, activation loop, DFG motif, gatekeeper residue, and hydrophobic back pocket. Representative c-Met residues frequently involved in inhibitor recognition are highlighted, including Leu1157 (gatekeeper residue), Met1160 (hinge residue), Gly1163 (solvent-front region), Asp1222-Phe1223-Gly1224 (DFG motif), and Tyr1230/Tyr1234/Tyr1235 within the activation loop. Type Ia/Ib inhibitors bind within the ATP-binding site in the active DFG-in conformation, whereas Type II inhibitors extend into the hydrophobic back pocket exposed in the DFG-out conformation. Allosteric inhibitors bind outside the ATP-binding cleft. Residue numbering corresponds to the human c-Met kinase domain.

Researchers have extensively targeted the catalytic kinase domain of c-Met to develop small-molecule inhibitors. Based on their binding mode within the kinase domain, c-MetT inhibitors are commonly classified as Type Ia, Type Ib, Type II, and Type III agents. Type I inhibitors bind the active DFG-in conformation of the kinase and occupy the ATP-binding site, whereas Type II inhibitors recognize the inactive DFG-out conformation and extend into a neighboring hydrophobic pocket. In contrast, Type III inhibitors act allosterically by binding regions outside the ATP-binding cleft. These distinct binding modes have profoundly shaped modern c-Met inhibitor design, enabling optimization of potency, selectivity, resistance profiles, and pharmacokinetic properties (Figure 1).

The availability of c-Met co-crystal structures has been central to inhibitor discovery, enabling the rational optimization of hinge interactions, hydrophobic pocket occupancy, coverage of resistance mutations, and multitarget pharmacophore design.

Type I inhibitors are classified into two subtypes, Type Ia and Type Ib, based on their interactions within the ATP-binding site. Type Ia inhibitors engage the solvent-front residue Gly1163 in addition to residues within the hinge and activation-loop regions, whereas Type Ib inhibitors avoid direct interaction with Gly1163 and penetrate more deeply into adjacent hydrophobic regions of the kinase domain. This distinction has played an important role in the evolution from early multitarget c-Met inhibitors to highly selective clinical agents.

### 2.1. Type I c-Met Inhibitors

Type I inhibitors bind the active DFG-in conformation of c-Met and remain the most clinically successful class of c-Met-directed therapeutics. By occupying the ATP-binding cleft and engaging key interactions with hinge-region residues, these inhibitors potently suppress c-Met kinase activity and have been central to validating c-Met as a therapeutic target in oncology. Structurally, these inhibitors bind to the U-shaped ATP-binding pocket of the kinase domain, typically interacting with the critical hinge residue Met1160 and forming key π–π stacking interactions with Tyr1230. Recent clinical data indicate that specific point mutations within the active site frequently lead to acquired resistance against type I inhibitors [20,21,22].

#### 2.1.1. Type Ia c-Met Inhibitors

**Figure 2:**
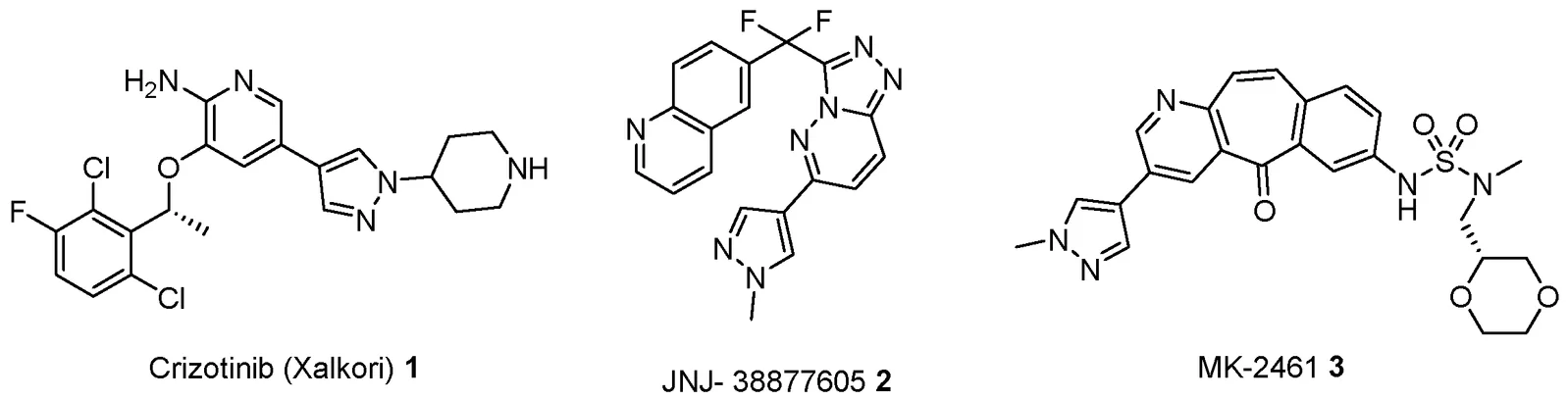
Clinically approved and investigational type Ia c-Met inhibitors.

Type Ia inhibitors represent the earliest generation of ATP-competitive c-Met inhibitors and established the feasibility of c-Met-targeted therapy (Figure 2). These compounds interact with the hinge region, Tyr1230 in the activation loop, and the solvent-front residue Gly1163. Although these interactions can enhance kinase affinity, they may also increase cross-reactivity with other kinases, leading to broader kinase-inhibition profiles [23,24,25,26].

Crizotinib (Xalkori^®^, **1**) is widely regarded as the prototype Type Ia inhibitor (Figure 2). Initially developed as a c-Met inhibitor, it was later found to be a potent inhibitor of ALK (Ak strain transforming, a tyrosine kinase B or PKB) and ROS1 (c-Ros oncogene 1) and became the first Food and Drug Administration (FDA)-approved (approval in 2011) ALK inhibitor for metastatic non-small-cell lung cancer (NSCLC). Crizotinib remains an important example of early c-Met inhibitor design because its broad kinase profile highlights both the therapeutic advantages and limitations of multitarget inhibition. Despite its clinical success, resistance frequently emerges through secondary kinase-domain mutations or activation of bypass signaling pathways [25,26,27,28,29,30].

JNJ-38877605 (**2**) demonstrated a potent biochemical inhibition of c-Met, with an IC_50_ of 4.7 nM (Figure 2) [31,32,33,34]. However, clinical development was discontinued after severe renal toxicity was observed in Phase I. Subsequent studies attributed this toxicity to precipitation of poorly soluble metabolites in the renal tubules rather than to direct c-Met inhibition. This case highlighted the importance of balancing kinase potency with favorable metabolic and physicochemical properties during lead optimization.

MK-2461 (**3**) is often classified as a non-classical Type I inhibitor because it preferentially binds to the active kinase conformation. In addition to c-Met, it inhibits the receptor d’origine nantais (RON) and VEGFR1, demonstrating the structural flexibility achievable in the design of ATP-competitive kinase inhibitors. Although not clinically advanced, MK-2461 represents an important bridge between early multitarget inhibitors and later generations of c-Met-directed agents [35,36,37].

#### 2.1.2. Type Ib c-Met Inhibitors

Type Ib inhibitors were developed to overcome the selectivity limitations of first-generation Type Ia inhibitors while maintaining potent ATP-competitive inhibition. Unlike Type Ia inhibitors, these molecules avoid direct interaction with Gly1163 and instead extend deeper into the hydrophobic regions of the ATP-binding site. This design strategy has yielded highly selective inhibitors with improved clinical performance [24,25,26].

**Figure 3:**
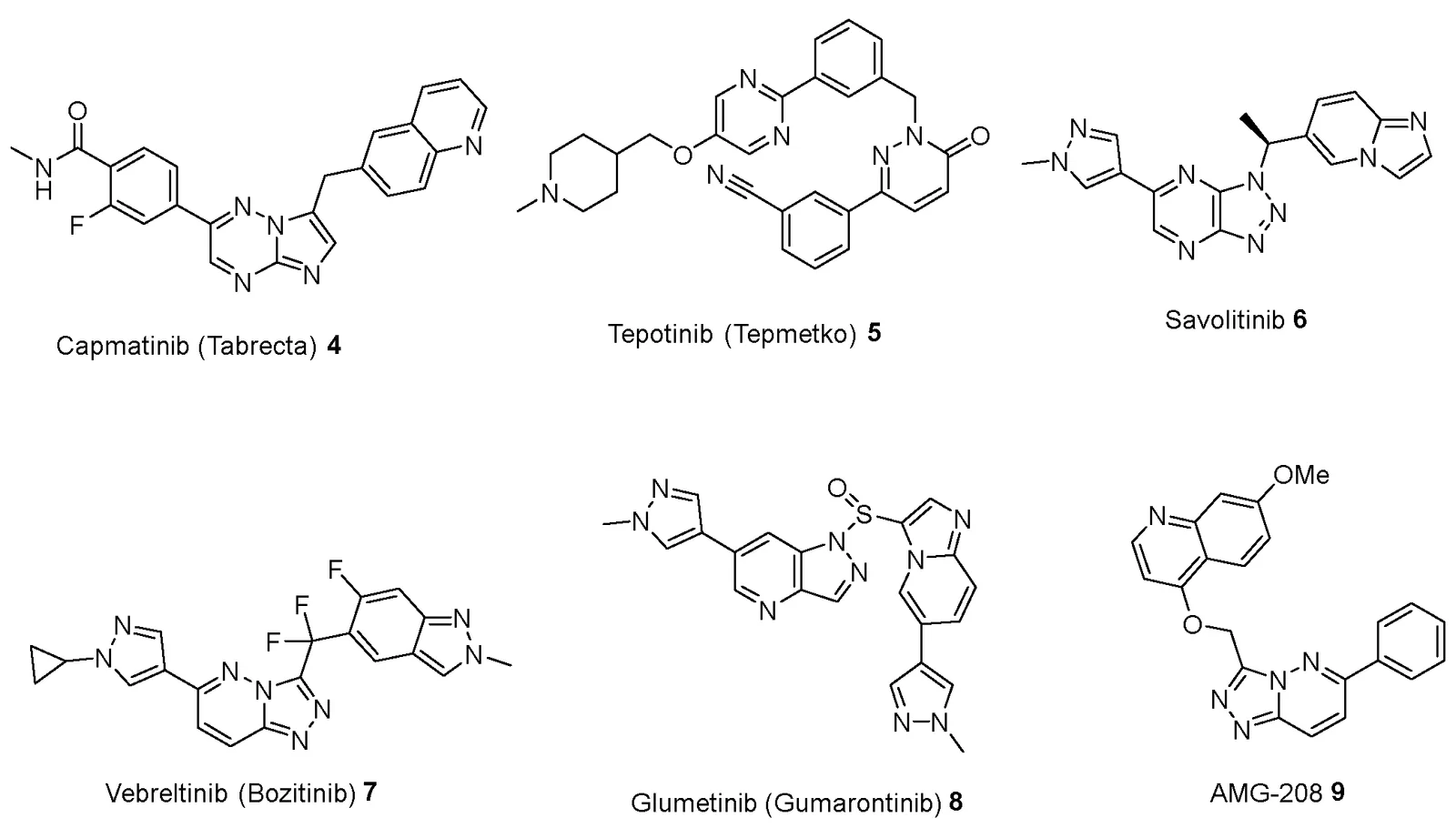
Clinically approved and investigational type Ib c-Met inhibitors.

Capmatinib (Tabrecta^®^, **4**), FDA-approved in 2020 [38,39,40,41], and tepotinib (Tepmetko^®^, **5**), which received FDA and European regulatory approvals in 2021 and 2022, respectively [42,43,44,45], represent the most significant clinical achievements in this class (Figure 3). Both agents received regulatory approval for the treatment of NSCLC harboring c-Met exon 14-skipping alterations and demonstrated that careful optimization of ATP-pocket interactions can substantially improve selectivity while maintaining potent c-Met inhibition.

The clinical landscape of Type Ib inhibitors has expanded with the development of savolitinib **6** [46,47,48,49,50], vebreltinib **7** [51,52], glumetinib **8** [53,54], and AMG-208 **9** [55,56,57]. Savolitinib has been approved in China for c-Met-driven NSCLC and is being evaluated in additional malignancies. Vebreltinib and glumetinib were also approved in China in 2023 for NSCLC harboring c-Met exon 14-skipping alterations. AMG-208 differs from the preceding compounds by combining c-Met inhibition with activity against RON, underscoring the continued interest in rational dual-target designs (Table 1).

### 2.2. Type II c-Met Inhibitors

**Figure 4:**
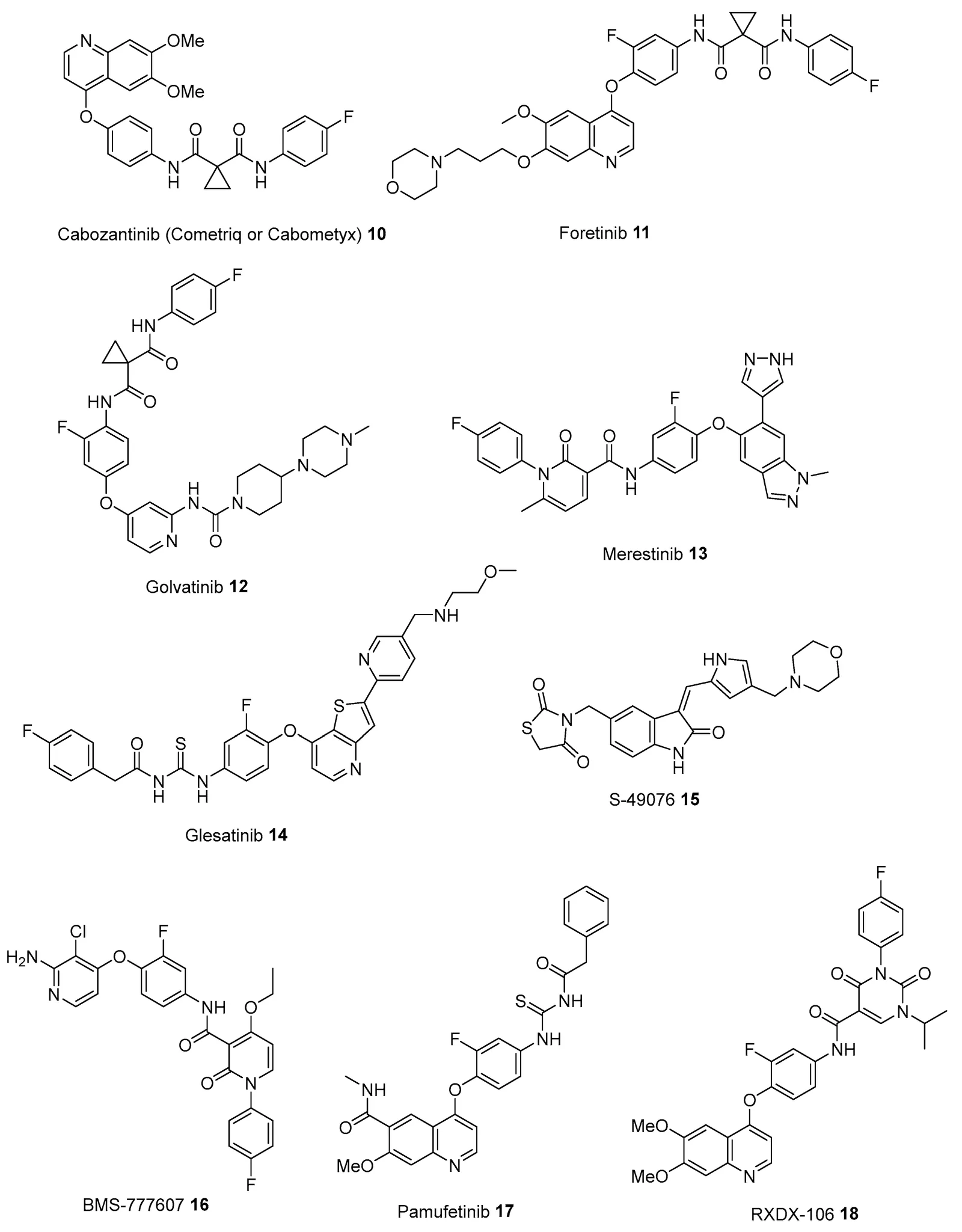
Clinically approved and investigational type II c-Met inhibitors.

Type II inhibitors constitute the second major class of ATP-competitive c-Met inhibitors (Figure 4, Table 2). Unlike Type I inhibitors, they bind the inactive DFG-out conformation of the kinase and extend into a hydrophobic pocket adjacent to the ATP-binding site. This binding mode broadens the interaction network available to the inhibitor and has enabled the development of compounds with broader kinase coverage and, in some cases, activity against resistance-associated c-Met variants.

Cabozantinib (Cabometyx^®^, **10**) is the most clinically successful Type II c-Met inhibitor and serves as the prototype for this class. It inhibits c-Met, VEGFR2, AXL, RET, FLT3 (Fms like tyrosine kinase 3), KIT, and ROS1, thereby targeting multiple pathways involved in tumor growth, angiogenesis, invasion, and metastasis. This broad target profile has supported its approval for renal cell carcinoma, hepatocellular, medullary thyroid carcinoma, and differentiated thyroid carcinoma (FDA approval in 2016, 2019, and 2021, respectively) [58,59].

Foretinib **11** is another representative Type II inhibitor targeting c-Met, VEGFR2, RON, AXL, and TIE2 [60,61,62,63,64]. Its quinoline-based architecture enables simultaneous occupation of the ATP-binding pocket and hydrophobic back pocket characteristic of DFG-out inhibitors. Although foretinib demonstrated promising preclinical activity, clinical development was limited by pharmacokinetic variability and toxicity associated with broad kinase inhibition.

Several additional investigational compounds have expanded the diversity of the Type II inhibitor class. Golvatinib **12** [65,66,67,68,69,70], merestinib **13** [71,72,73,74], glesatinib **14** [75,76,77,78], S-49076 **15** [79,80,81,82], BMS-777607 **16** [83,84,85,86,87,88,89], pamufetinib **17** [90,91,92,93,94,95,96,97], and RXDX-106 **18** [98,99,100] combine c-Met inhibition with activity against AXL, RON, VEGFR, TAM family kinases, or related targets. Collectively, these compounds reflect a continuing trend toward rational polypharmacology as a strategy for addressing pathway redundancy and resistance.

### 2.3. Type III c-Met Inhibitors

Type III inhibitors are the least explored class of c-Met-directed therapeutics. Unlike Type I and Type II inhibitors, these compounds do not compete directly with ATP but instead bind to allosteric sites outside the ATP-binding cleft. Because allosteric binding sites are generally less conserved across the kinome, Type III inhibition offers the potential for improved selectivity and activity against ATP-site resistance mutations.

**Figure 5:**
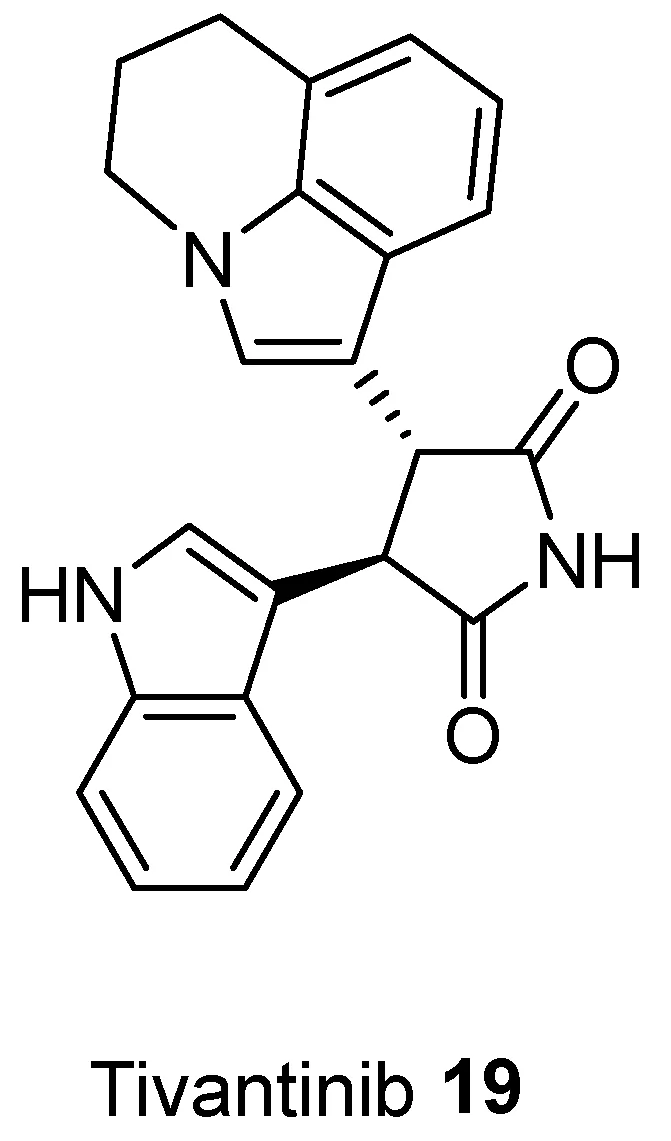
Tivantinib (**19**), historically classified as a Type III c-Met inhibitor.

Tivantinib (ARQ 197, **19**) was initially proposed as the first selective, non-ATP-competitive c-Met inhibitor and advanced to multiple Phase II and Phase III clinical studies in hepatocellular carcinoma, NSCLC, gastric cancer, colorectal cancer, and other malignancies (Figure 5) [101,102,103,104,105,106,107]. Early reports suggested that tivantinib stabilized an inactive c-Met conformation without directly competing with ATP, generating considerable interest in allosteric c-Met inhibition.

Subsequent mechanistic studies, however, demonstrated that tivantinib exerts much of its antiproliferative activity independently of c-Met inhibition. Instead, its primary mechanism appears to involve disrupting microtubule polymerization by directly interacting with tubulin, leading to mitotic arrest and apoptosis. The tivantinib story therefore serves as a prominent reminder that potent cellular activity does not necessarily validate the originally proposed molecular target and underscores the importance of rigorous target-validation studies in kinase inhibitor discovery.

### 2.4. Perspectives on c-Met Inhibitor Development

The evolution of c-Met inhibitors illustrates how structural insights into kinase binding modes have guided the transition from early multitarget Type Ia inhibitors to highly selective Type Ib agents and broader Type II inhibitors designed to address pathway redundancy and resistance. Although clinically approved therapies have firmly established c-Met as a druggable oncogenic driver, acquired resistance, mutation coverage, kinase selectivity, and durable pathway suppression remain important challenges. These limitations continue to drive the development of mutation-directed inhibitors, rational multitarget agents, allosteric modulators, covalent inhibitors, and targeted protein-degradation approaches, all of which are expected to play increasingly important roles in the next generation of c-Met-directed therapeutics.

## 3. Natural Products as Lead Scaffolds for c-Met Inhibitor Discovery

Natural products have long been important sources of biologically active molecules and continue to inspire modern drug discovery. Their structural diversity, stereochemical complexity, and broad pharmacological activities have contributed to the development of numerous approved therapeutics for oncology, infectious diseases, cardiovascular disorders, and inflammatory conditions. In medicinal chemistry, natural products often provide valuable lead structures for designing synthetic and semisynthetic analogs with improved potency, selectivity, and drug-like properties.

Several naturally occurring compounds have been reported to modulate HGF/c-Met signaling through distinct mechanisms, including inhibition of c-Met phosphorylation, suppression of downstream signaling pathways, interference with ligand-dependent receptor activation, and, in some cases, direct interaction with the kinase domain [108]. Although their potency is generally lower than that of clinically approved ATP-competitive c-Met inhibitors, natural products remain valuable sources of structural inspiration. Their ability to modulate multiple oncogenic pathways may also confer advantages in addressing pathway redundancy and adaptive resistance.

**Figure 6:**
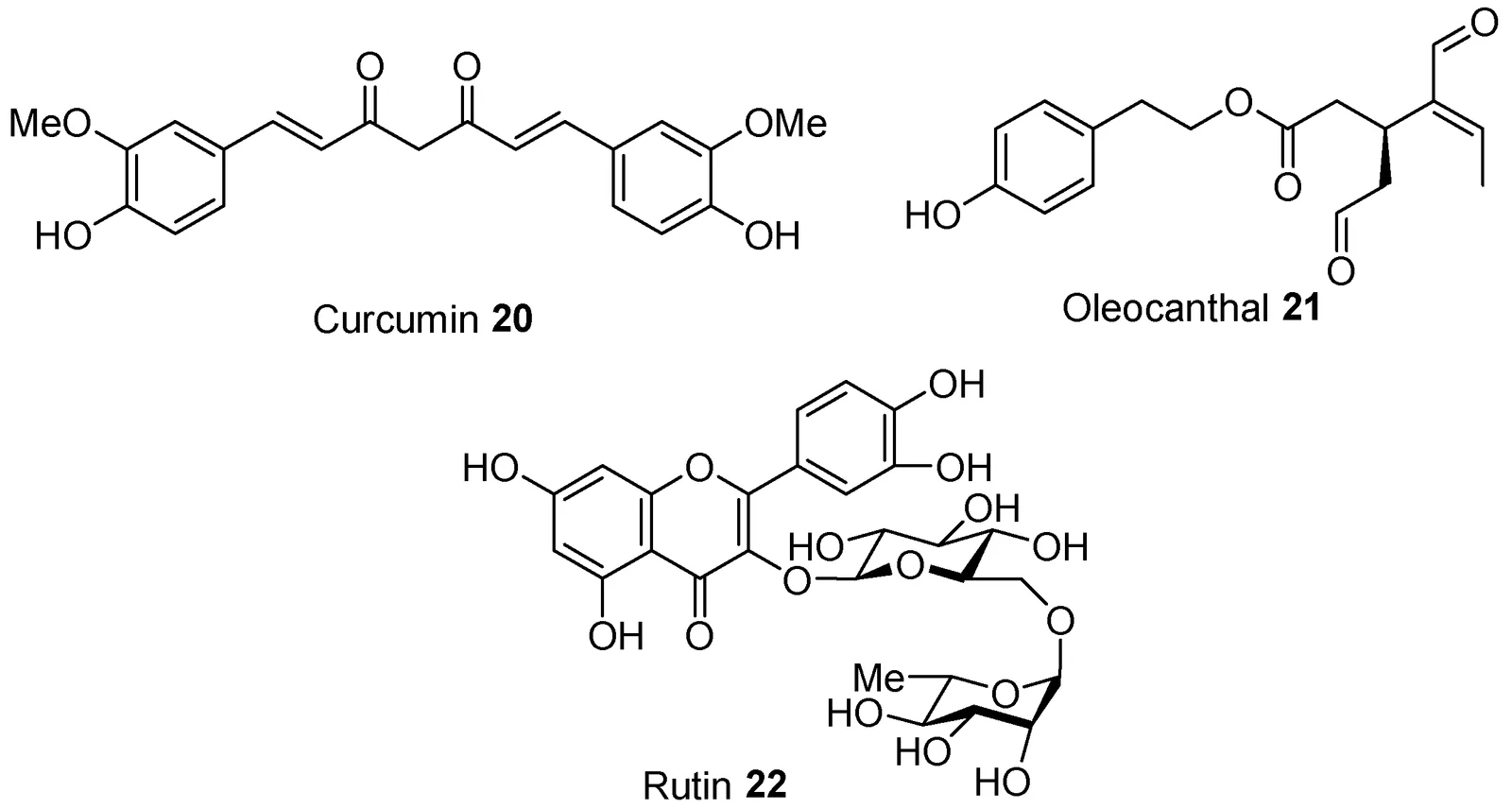
Curcumin **20**, oleocanthal **21**, and rutin **22**, natural compounds with c-Met inhibitory properties.

Among the most extensively studied natural c-Met modulators are curcumin **20**, oleocanthal **21**, and rutin **22** (Figure 6). Curcumin, a polyphenolic diarylheptanoid from *Curcuma longa*, suppresses HGF-induced c-Met phosphorylation and attenuates PI3K/AKT and MAPK signaling, thereby reducing proliferation, migration, invasion, and EMT in multiple cancer models [109,110,111].

Oleocanthal **21**, a secoiridoid phenolic compound in extra-virgin olive oil, has also shown anticancer activity by inhibiting c-Met phosphorylation and the downstream signaling pathways that drive invasion and metastasis. Beyond modulating c-Met signaling, oleocanthal exhibits anti-inflammatory and antioxidant properties, suggesting a broader polypharmacological mechanism of action [112,113,114].

Rutin **22**, a naturally occurring flavonoid glycoside, has likewise been reported to suppress c-Met-associated signaling and to exhibit antioxidant, anti-inflammatory, and antiproliferative activities. Although its direct kinase-inhibitory potency appears limited, rutin demonstrates the ability of naturally occurring polyphenols to regulate multiple signaling networks implicated in tumor progression [115,116].

Despite showing encouraging biological activity, none of these compounds has advanced to clinical development as a c-Met-targeted therapeutic. Limited potency, poor metabolic stability, low oral bioavailability, and insufficient target selectivity remain major challenges. Consequently, these molecules are best viewed as lead scaffolds to guide future medicinal chemistry optimization.

**Figure 7:**
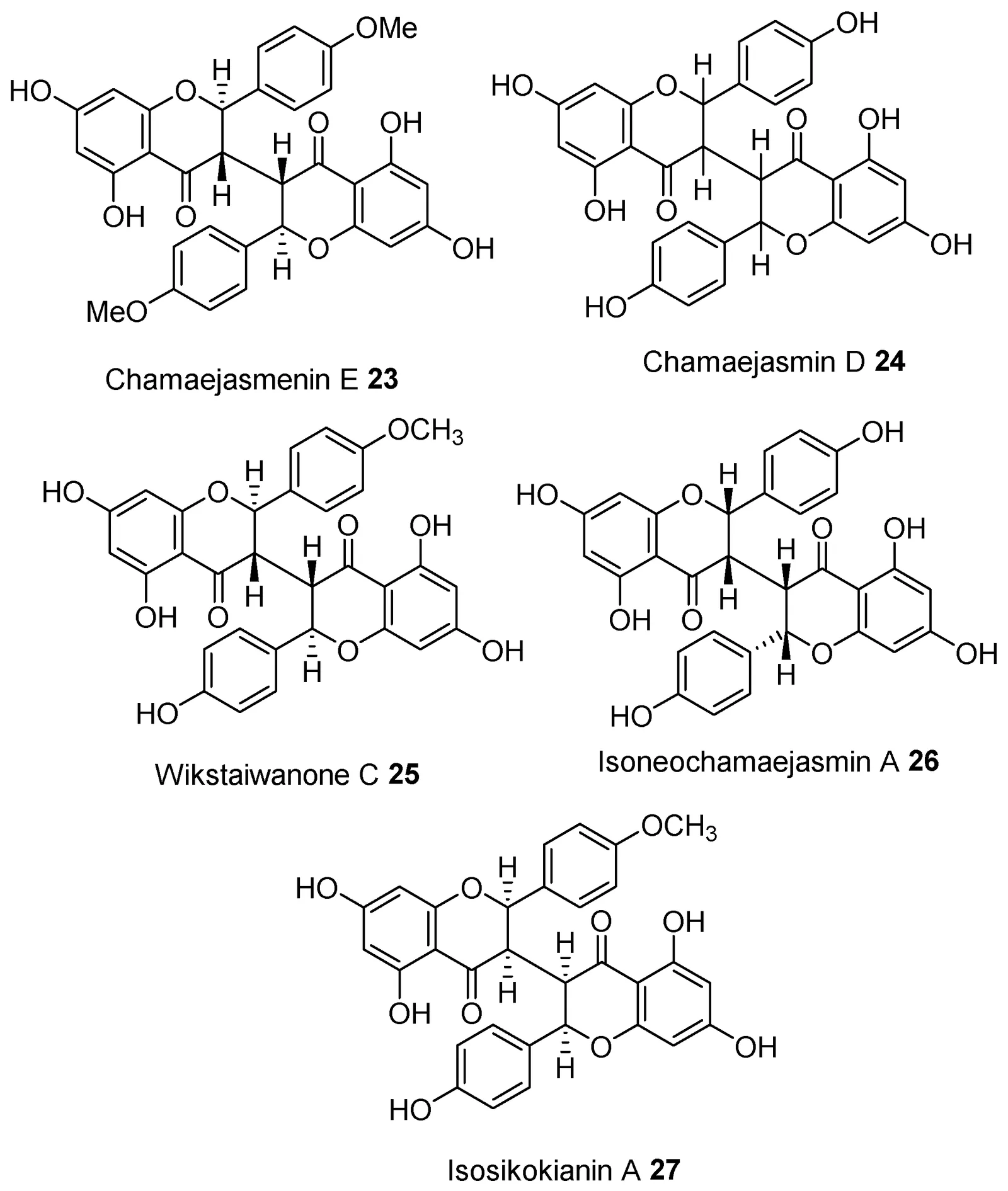
Natural biflavones, chamaejasmenin E **23**, chamaejasmin D **24**, wikstaiwanone C **25**, isoneochamaejasmin A **26**, and isosikokianin A **27**.

Flavonoids are among the most extensively studied classes of natural products and have attracted considerable attention as potential modulators of c-Met signaling. Among them, the biflavonoids chamaejasmenin E **23**, chamaejasmin D **24**, wikstaiwanone C **25**, isoneochamaejasmin A **26**, and isosikokianin A **27** were isolated from the roots of *Stellera chamaejasme* (Figure 7) [117].

Chamaejasmenin E **23** emerged as the most active member of the series, exhibiting IC_50_ values of 7.47 and 25.21 μM against Hep3B and SMMC-7721 hepatocellular carcinoma cells, respectively, and lower toxicity toward normal L-O2 hepatocytes (IC_50_ = 61.04 μM) [117]. In addition to inhibiting proliferation, compound **23** suppressed cancer-cell migration and induced caspase-dependent apoptosis. Mechanistic studies linked these effects to inhibition of HGF/c-Met signaling, and molecular docking of the c-Met kinase domain (PDB ID: 4R1V) provided a structural rationale for the proposed interaction [117]. However, direct biochemical inhibition of c-Met was not reported, so the compound should be regarded as a c-Met-pathway modulator rather than a confirmed c-Met kinase inhibitor.

**Figure 8:**
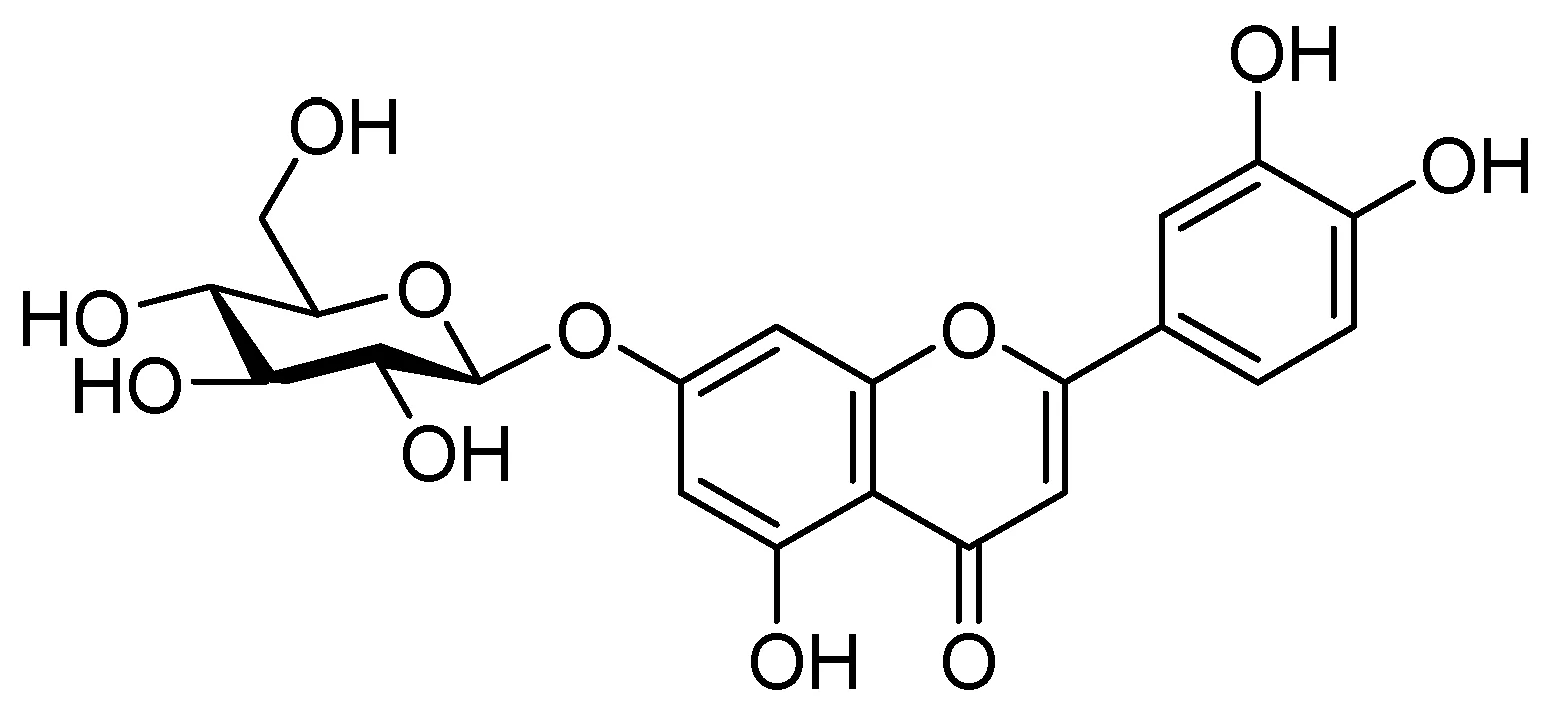
Natural flavonoid glycoside cynaroside **28**.

Another notable natural product is cynaroside **28** (luteolin-7-O-glucoside), a flavonoid glycoside widely distributed in medicinal plants (Figure 8). Cynaroside **28** inhibited the proliferation of HGC27, MKN-45, and SGC7901 gastric cancer cells while showing limited toxicity toward normal cells [118]. Treatment induced S-phase arrest, promoted apoptosis, and suppressed cancer cell migration and invasion. Mechanistic studies demonstrated reduced phosphorylation of c-Met and of downstream components of the AKT/mTOR pathway, including AKT, mTOR, and p70S6K [118].

Importantly, cynaroside also showed in vivo activity in NOD/SCID xenograft models established with MKN-45 and SGC7901 gastric cancer cells, with treatment significantly reducing tumor volume and weight without obvious systemic toxicity [118]. Although these results support further investigation, direct biochemical validation of c-Met inhibition remains lacking, and the observed effects may reflect modulation of multiple signaling pathways.

Natural products have played an important role in expanding the chemical space explored for c-Met-targeted drug discovery. Although most reported compounds act as pathway modulators rather than potent direct c-Met inhibitors, they highlight structural features, including polyphenolic frameworks, biflavonoid architectures, and glycosylated flavonoids, that may serve as useful starting points for future lead optimization. Their greatest value lies not as direct drug candidates but as sources of novel pharmacophores that can be incorporated into synthetic and semisynthetic scaffolds to improve potency, selectivity, and pharmacokinetic properties.

## 4. Synthetic Heterocyclic Scaffolds as c-Met Inhibitors

The discovery of small-molecule c-Met inhibitors has largely been driven by advances in medicinal chemistry, structural biology, and structure-based drug design. Although natural products have provided valuable lead compounds, synthetic chemistry has enabled systematic optimization of potency, selectivity, pharmacokinetic properties, metabolic stability, and resistance profiles. As a result, heterocyclic compounds have emerged as the dominant class of c-Met inhibitors and form the basis of both clinically approved drugs and numerous investigational candidates.

Heterocyclic scaffolds are particularly well-suited for kinase inhibitor design because they can mimic the adenine moiety of ATP and establish key interactions within the kinase hinge region. Incorporating nitrogen-, oxygen-, and sulfur-containing heterocycles allows for fine control of molecular geometry, electronic properties, lipophilicity, and target recognition. Strategic modification of these scaffolds has enabled the development of inhibitors with improved kinase selectivity, favorable drug-like properties, and activity against resistance-associated c-Met alterations.

Over the past decade, extensive medicinal chemistry efforts have yielded a broad range of c-Met-directed heterocyclic scaffolds, including indoles, pyridines, quinolines, pyrazoles, imidazoles, pyrimidines, quinazolines, quinoxalines, thiazoles, thienopyridines, pyrrolo[2,3-*d*]pyrimidines, pyrazolo[3,4-*b*]pyridines, triazolotriazines, benzothiazoles, and related fused-ring systems. These scaffolds have produced compounds ranging from multitarget kinase inhibitors to highly selective c-Met-directed agents, with activities spanning the low-nanomolar to subnanomolar range [119,120,121,122].

The following sections are organized around the core heterocyclic scaffold used in inhibitor design. This review emphasizes design rationale, synthetic strategy, biological activity, target-validation evidence, and key structural features associated with c-Met inhibition. This scaffold-based organization provides a framework for understanding the evolution of c-Met inhibitor design and highlights the medicinal chemistry principles that continue to guide the development of next-generation selective and multitarget anticancer agents.

### 4.1. Indole-Based c-Met Inhibitors

Indole and indolin-2-one are widely used heterocyclic scaffolds in kinase inhibitor discovery for their favorable electronic properties, conformational rigidity, and capacity to engage in hydrogen-bonding, hydrophobic, and π-π interactions within ATP-binding sites. Their structural versatility and frequent occurrence in anticancer agents have made them attractive platforms for developing c-Met-directed inhibitors.

**Scheme 1:**
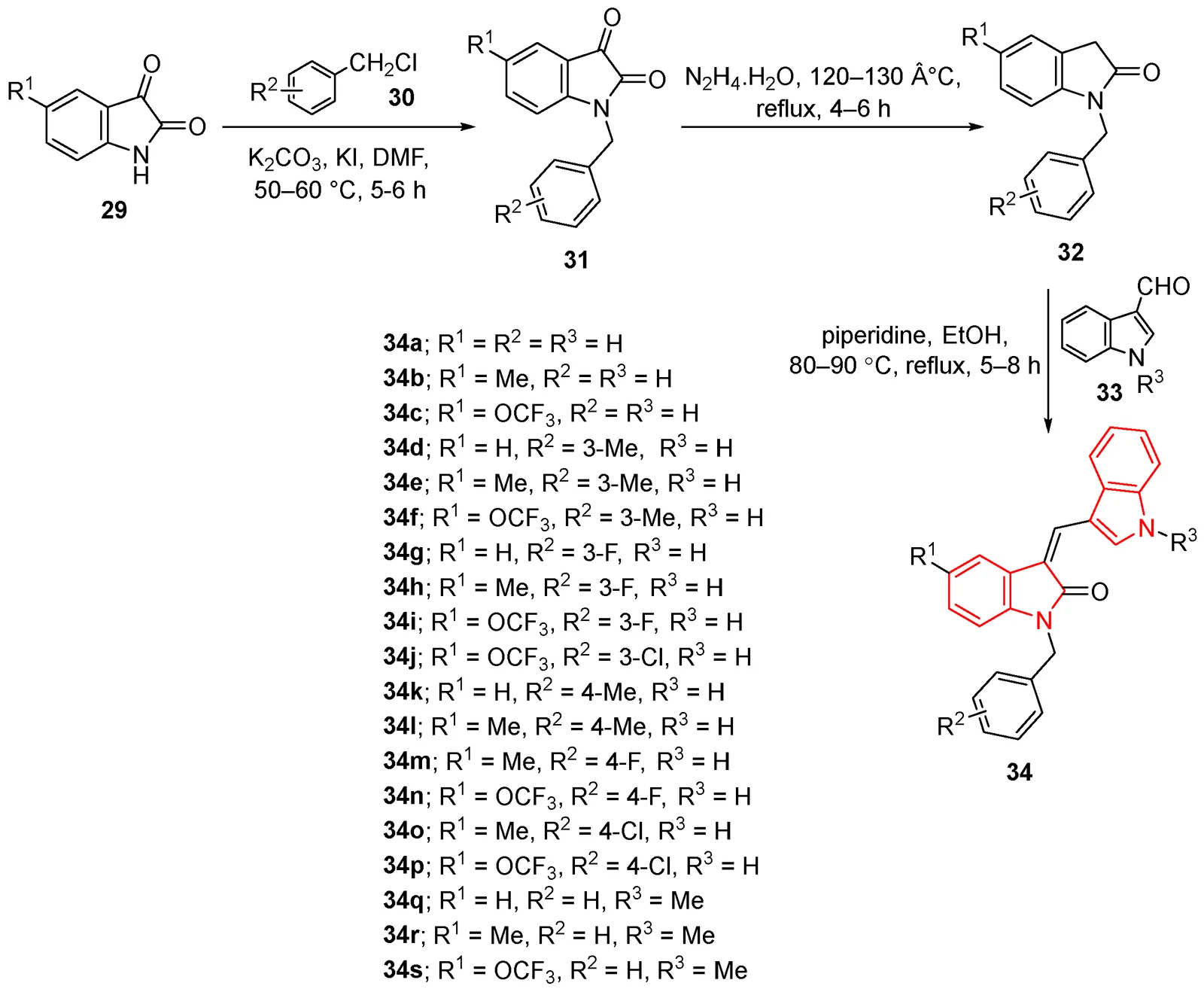
Synthetic route toward 1-benzyl-2-indolinones **34**.

Cihan-Üstündağ and colleagues reported a series of 1-benzyl-2-indolinone derivatives **34**, prepared by N-alkylation of substituted isatins **29**, followed by reduction and condensation with substituted indole-3-carbaldehydes **33** to produce the target compounds **34** (Scheme 1) [123].

Several derivatives showed antiproliferative activity against breast, lung, renal, colorectal, pancreatic, and other cancer cell lines. Compound **34h** (R^1^ = Me, R^2^ = 3-F, R^3^ = H) was the most active analog, with IC_50_ values ranging from 0.54 to 8.76 μM across the tested cell panel, and it outperformed sunitinib in several models. Compound **34s** also showed notable activity, particularly against MCF7 and MIA PaCa-2 cells. Both compounds exhibited relatively low toxicity toward the evaluated nonmalignant cells. Structure–activity relationship (SAR) analysis showed that the methyl group at the 5-position of the indolinone heterocycle enhances antiproliferative activity.

Docking studies of the c-Met kinase domain (PDB ID: 2RFS) suggested favorable accommodation of **34h** and **34s** within the ATP-binding pocket [123]. However, because the study relied primarily on cellular activity and molecular modeling, the extent to which direct c-Met inhibition contributes to the observed antiproliferative effects remains uncertain. Biochemical kinase assays and target-engagement studies are required to establish these compounds as validated c-Met inhibitors.

**Scheme 2:**
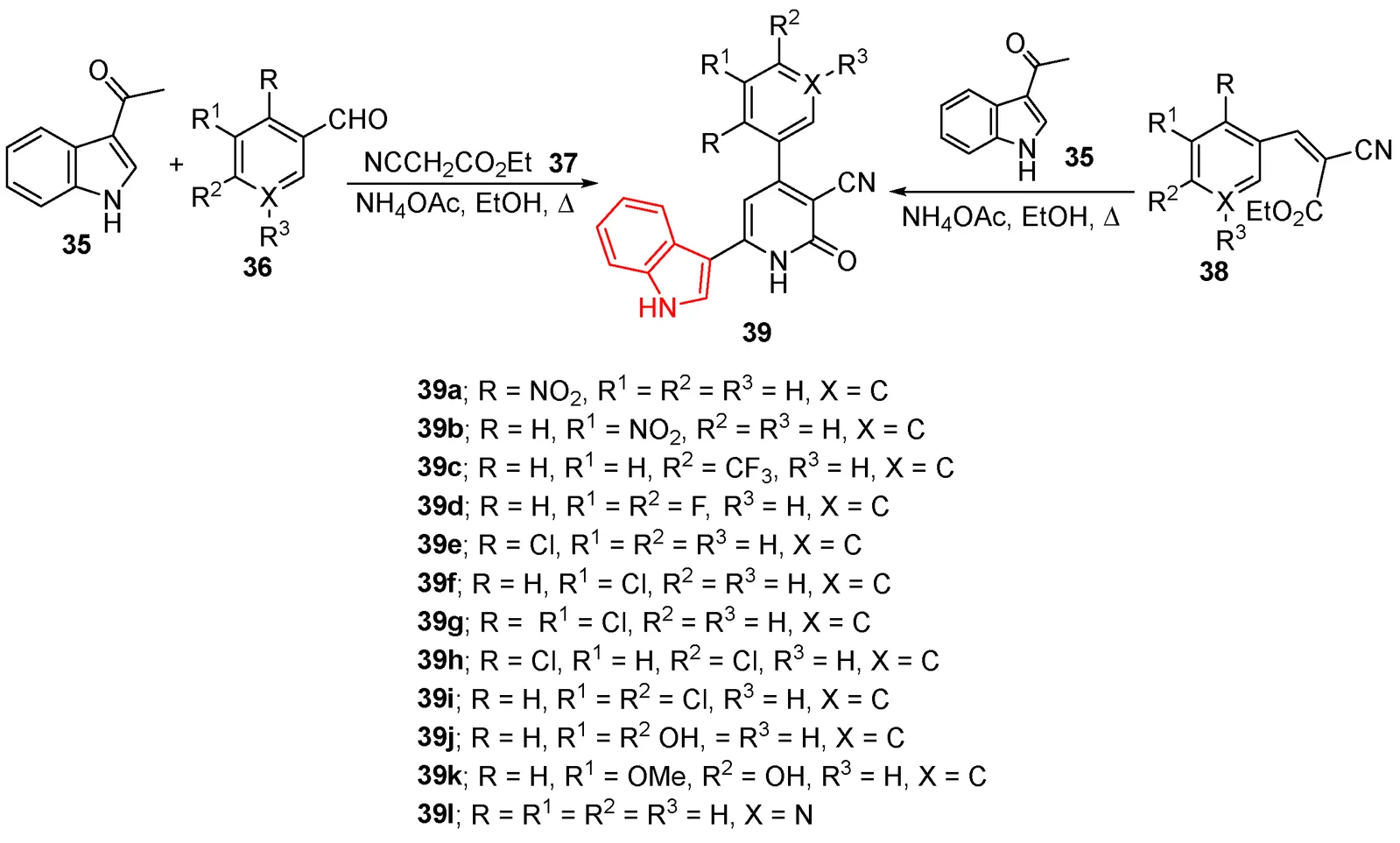
Synthetic route toward 6-(3-indolyl)-1*H*-pyrid-2-one-3-carbonitriles **39**.

To further diversify indole-containing kinase inhibitors, a series of 6-(3-indolyl)-1*H*-pyrid-2-one-3-carbonitriles **39** was synthesized via a multicomponent reaction of 3-acetylindole **35**, aromatic aldehydes **36**, and ethyl cyanoacetate **37** (Scheme 2) [124].

Among the synthesized compounds, **39h** exhibited the most favorable biological profile, showing strong growth inhibition against SR leukemia, HOP-92 lung, RXF 393 renal, and HCT116 colorectal cancer cells, while showing limited toxicity toward several nonmalignant cell lines.

Unlike many studies discussed in this review, this study provided direct biochemical evidence. Compound **39h** inhibited c-Met, EGFR, B-Raf, CDK4, and CDK6, with IC_50_ values of 205, 281, 112, 95, and 184 nM, respectively. This activity profile identifies **39h** as a multitarget kinase inhibitor rather than a selective c-Met inhibitor. Flow-cytometric analysis demonstrated G_1_/S-phase arrest, consistent with the observed inhibition of CDK4/6.

Molecular docking and molecular dynamics simulations indicated stable binding within the ATP-binding sites of the targeted kinases and provided a structural rationale for the experimentally observed kinase profile [124]. Because direct enzyme inhibition was demonstrated, **39h** can be classified as an experimentally validated multitarget inhibitor, with c-Met as one of its molecular targets.

**Scheme 3:**
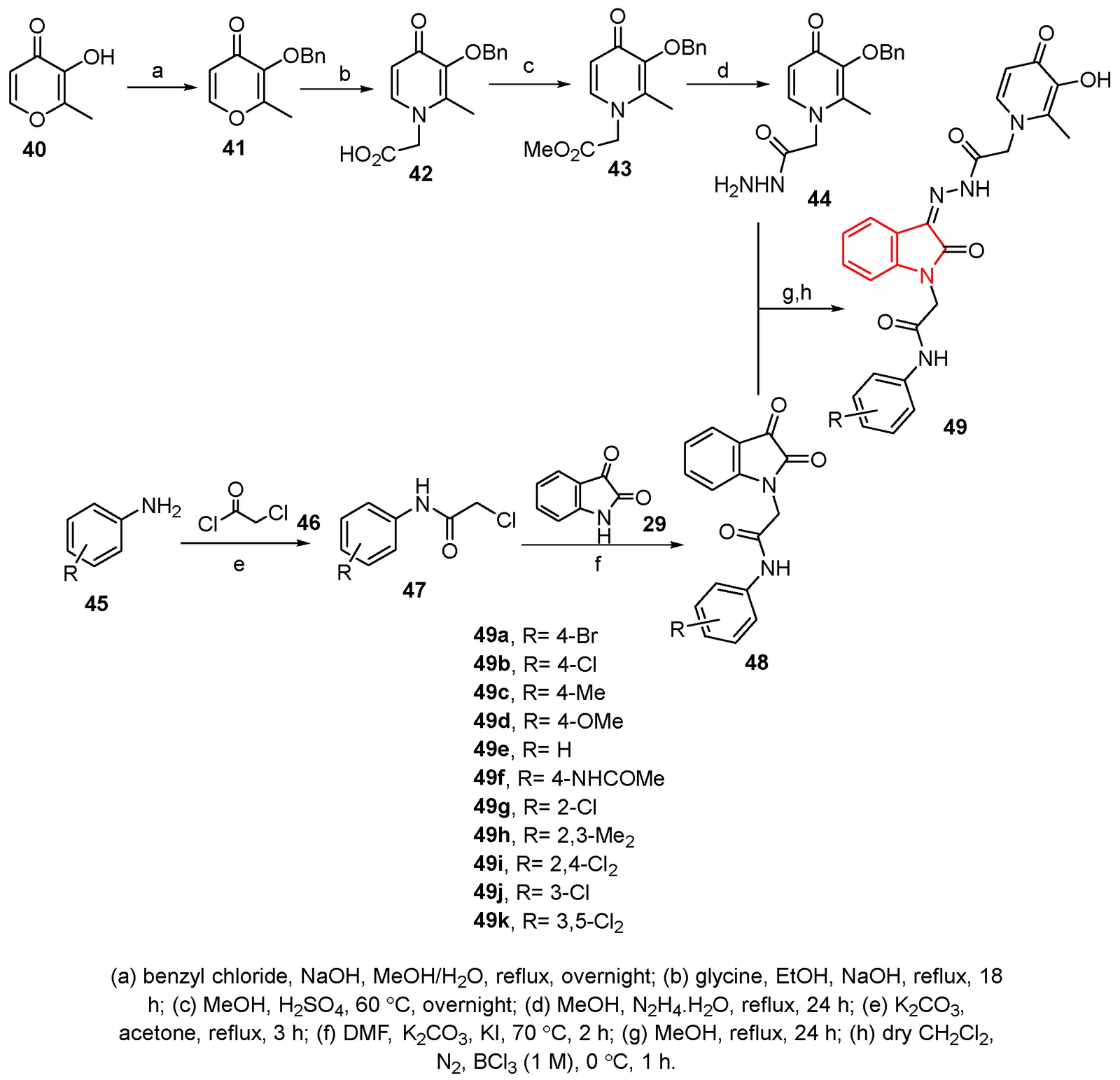
Synthetic route toward hydrazone-linked indolin-2-one derivatives **49** containing a 3-hydroxy-4-pyridinone pharmacophore.

A distinct molecular conjugation strategy combined an indolin-2-one scaffold with a 3-hydroxy-4-pyridinone pharmacophore via a hydrazone linker. Shirvani and co-workers prepared a series of derivatives **49** through a multistep sequence that involved constructing the pyridinone-derived acetohydrazide **44**, preparing isatin-derived intermediates **48**, and finally condensing followed by debenzylation to afford the target compounds (Scheme 3) [125].

Biological evaluation identified **49k**, bearing a 3,5-dichlorophenyl substituent, as the most active derivative. The favorable activity associated with this substitution pattern suggests that hydrophobic and electron-withdrawing aromatic groups contribute positively to the biological profile, whereas the hydrazone linker and pyridinone fragment provide additional hydrogen-bonding functionality.

Docking studies using the c-Met structure (PDB ID: 3LQ8) predicted favorable accommodation of **49k** within the ATP-binding site and suggested c-Met as a possible target [125]. However, neither direct c-Met inhibition nor cellular target engagement experiments were reported. Therefore, **49k** should be considered a putative c-Met-directed compound rather than a confirmed c-Met inhibitor.

The indole scaffold remains a versatile platform for c-Met-directed drug discovery, supporting both validated multikinase inhibitors and structurally diverse compounds with proposed c-Met-associated activity.

### 4.2. Pyridine-Based c-Met Inhibitors

Pyridine is one of the most widely used heterocyclic scaffolds in kinase inhibitor design and is frequently incorporated into ATP-competitive c-Met inhibitors. The pyridine nitrogen can serve as a hinge-binding hydrogen-bond acceptor, while the aromatic ring provides a versatile platform for scaffold hopping, linker attachment, and peripheral substitution. These features have made pyridine-based architectures valuable for the development of both selective c-Met inhibitors and rationally designed multitarget agents.

**Scheme 4:**
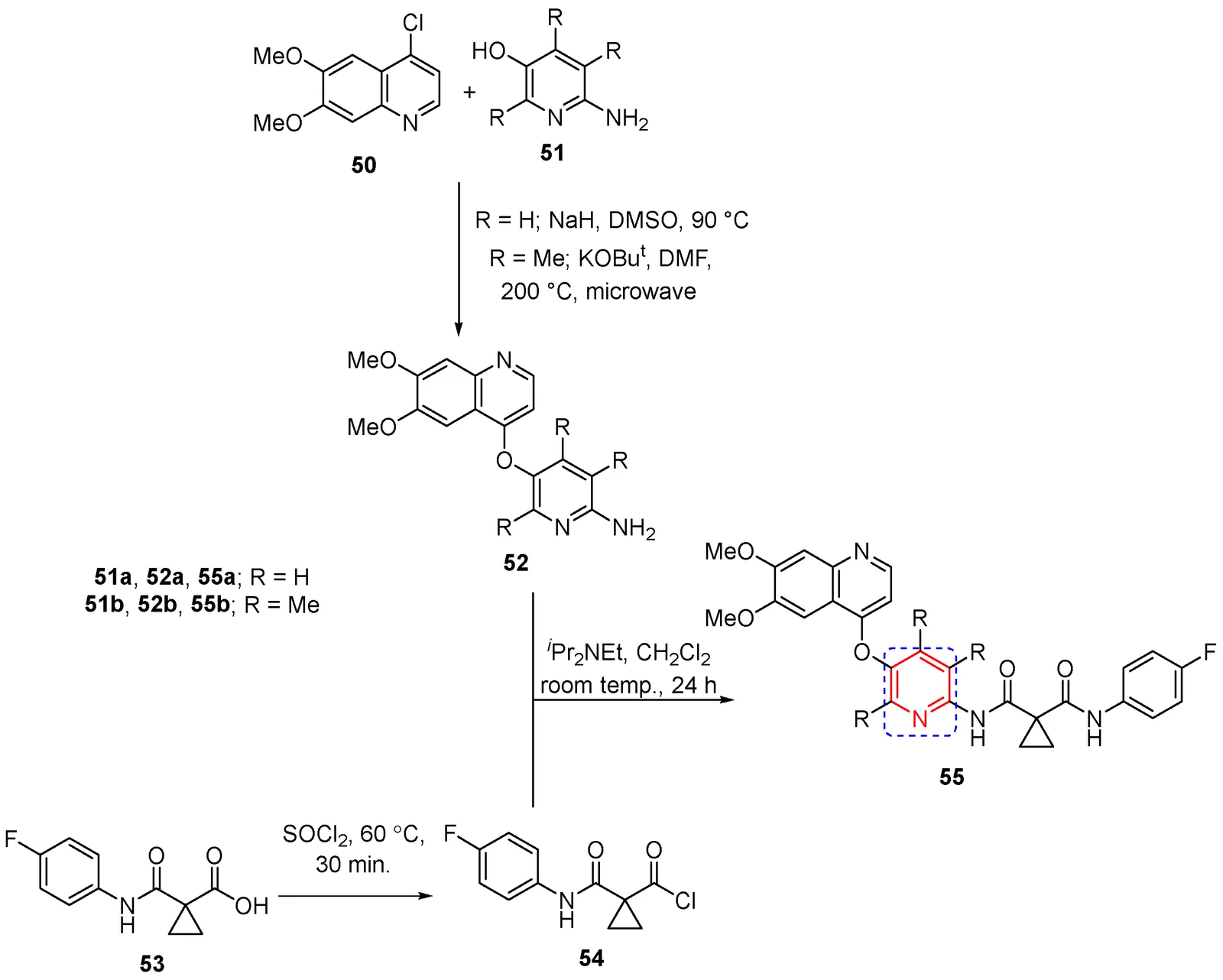
Synthetic route toward pyridine-containing cabozantinib bioisosteres **55**.

Inspired by the clinical success of cabozantinib **10**, a scaffold-hopping strategy was explored in which the central phenyl ring was replaced with a pyridine nucleus, yielding a series of pyridine-containing cabozantinib analogs **55** (Scheme 4) [126]. The target compounds were prepared by coupling substituted 2-amino-5-hydroxypyridines with 4-chloro-6,7-dimethoxyquinoline, followed by amide formation with a cyclopropanecarboxamide-containing side chain.

Among the synthesized analogs, **55a** emerged as the lead compound. It inhibited c-Met with an IC_50_ of 4.9 nM, compared with 5.4 nM for cabozantinib, indicating that replacing the central phenyl ring preserved potent kinase inhibition [126]. More notably, **55a** showed improved cellular activity against Hep3B, Huh7, A549, and H1299 cancer cells, suggesting that the pyridine replacement favorably influenced cellular performance despite only modest changes in biochemical potency.

Mechanistic studies showed greater induction of apoptosis and stronger antiangiogenic effects than cabozantinib. Docking studies using the c-Met kinase structure (PDB ID: 5DG5) indicated that **55a** retained cabozantinib’s characteristic binding orientation, including a hydrogen-bonding interaction between the amide NH and Asp1222 [126]. Collectively, these findings demonstrate that pyridine can serve as an effective phenyl bioisostere while preserving key c-Met-recognition elements.

**Scheme 5:**
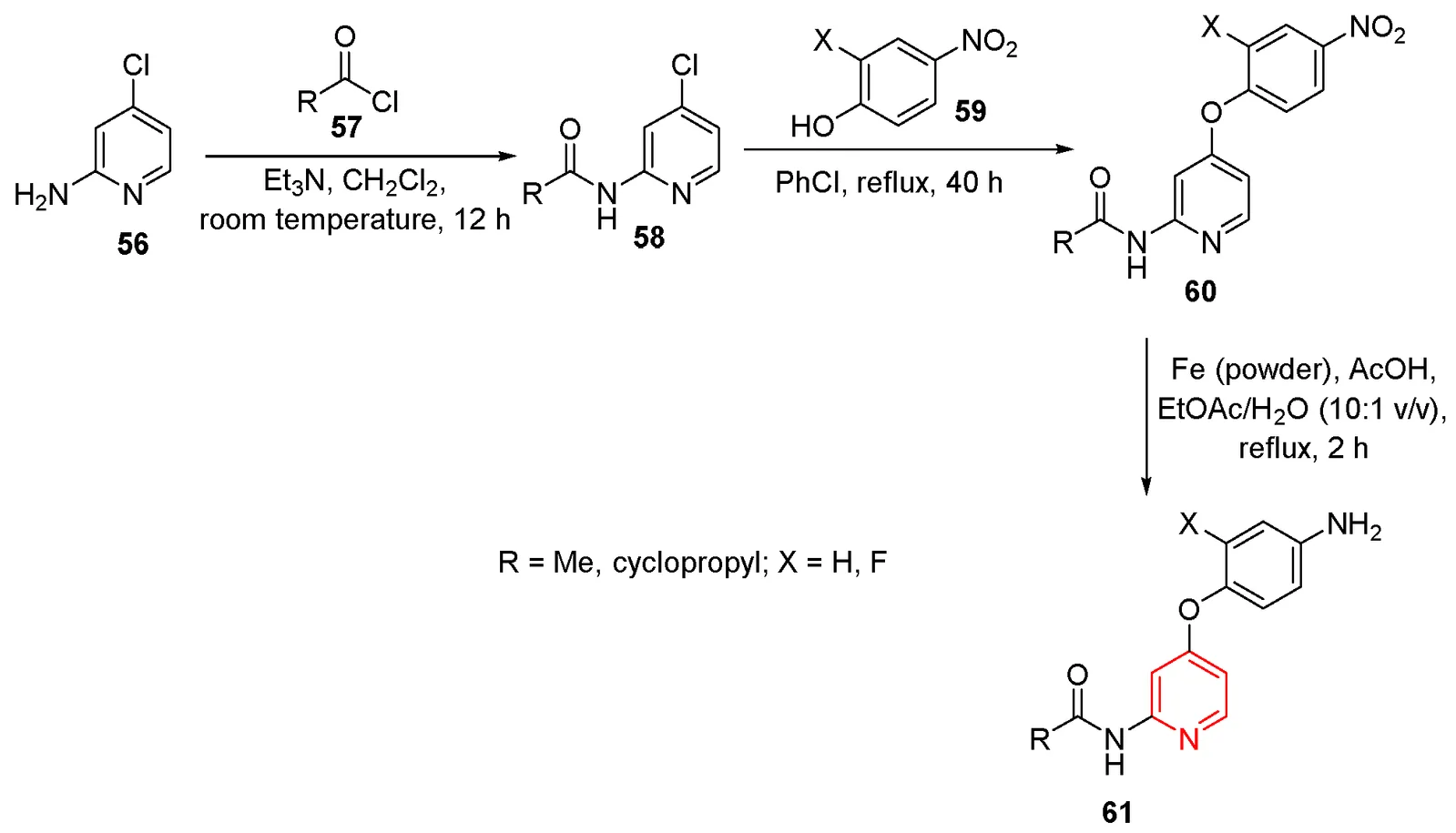
Synthetic route toward 4-(4-aminophenoxy)pyridine intermediates.

**Scheme 6:**
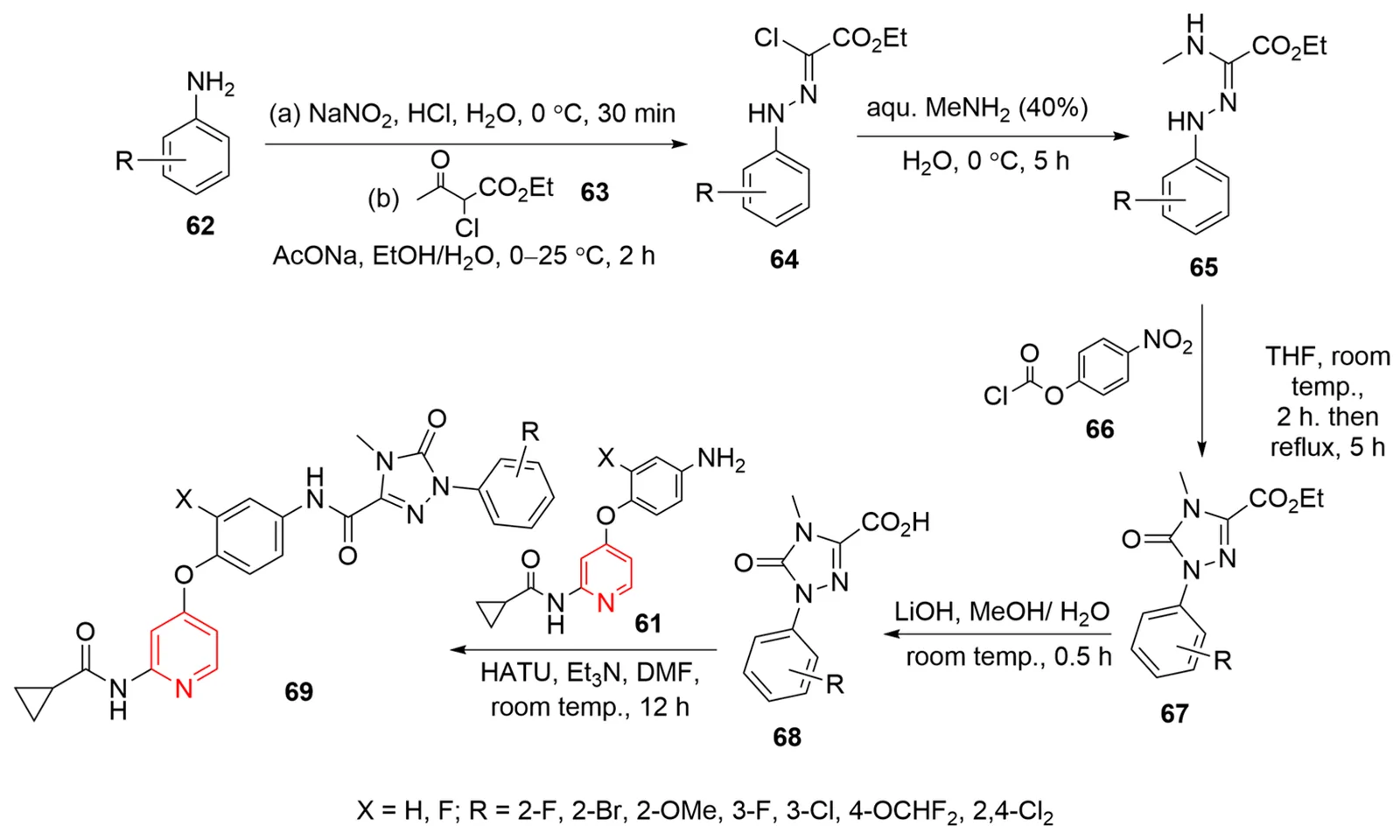
Synthetic route toward 4-phenoxypyridine-linked 1,2,4-triazole-3-carboxamides **69**.

**Scheme 7:**
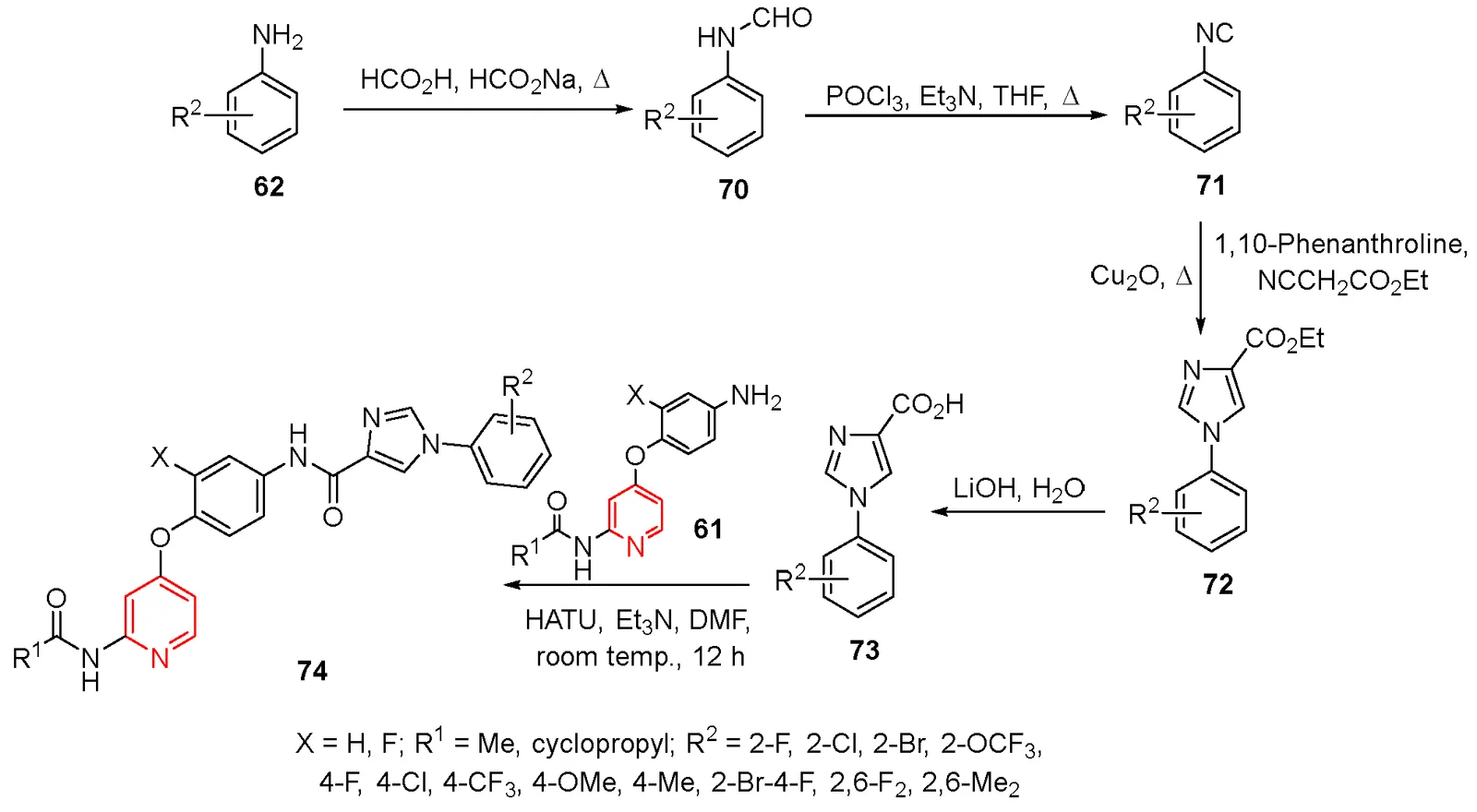
Synthetic route toward 4-phenoxypyridine-linked imidazole-4-carboxamides **74**.

Building on the pharmacophoric features common to several Type II c-Met inhibitors, a series of 4-phenoxypyridine derivatives was designed that incorporate either the 1,2,4-triazole-3-carboxamide **69** or the imidazole-4-carboxamide **74** motifs [23]. The design retained a pyridine-based hinge-binding element, a 4-phenoxy linker, and a terminal hydrogen-bonding region characteristic of many Type II inhibitors (Scheme 5, Scheme 6 and Scheme 7).

Biological evaluation showed that the imidazole-4-carboxamide series generally exhibited superior activity. The lead compound from series **74**, featuring a cyclopropyl substituent and fluorinated aromatic groups, inhibited c-Met with an IC_50_ of 12 nM. Although less potent than foretinib (IC_50_ = 2.1 nM), it exhibited appreciable antiproliferative activity against MKN-45, A549, and H460 cells, as well as inhibition of colony formation and cancer-cell migration [23].

Docking studies suggested a binding mode consistent with Type II c-Met inhibition. The pyridine nitrogen and the cyclopropanecarboxamide NH interacted with Met1160, while the imidazole-4-carboxamide moiety formed a hydrogen bond with Asp1222. Additional halogen-bonding and hydrophobic interactions further stabilized the complex within the ATP-binding pocket [23]. These results underscore the importance of pairing hinge-binding pyridine motifs with appropriately positioned hydrogen-bonding pharmacophores to achieve potent c-Met inhibition.

**Scheme 8:**
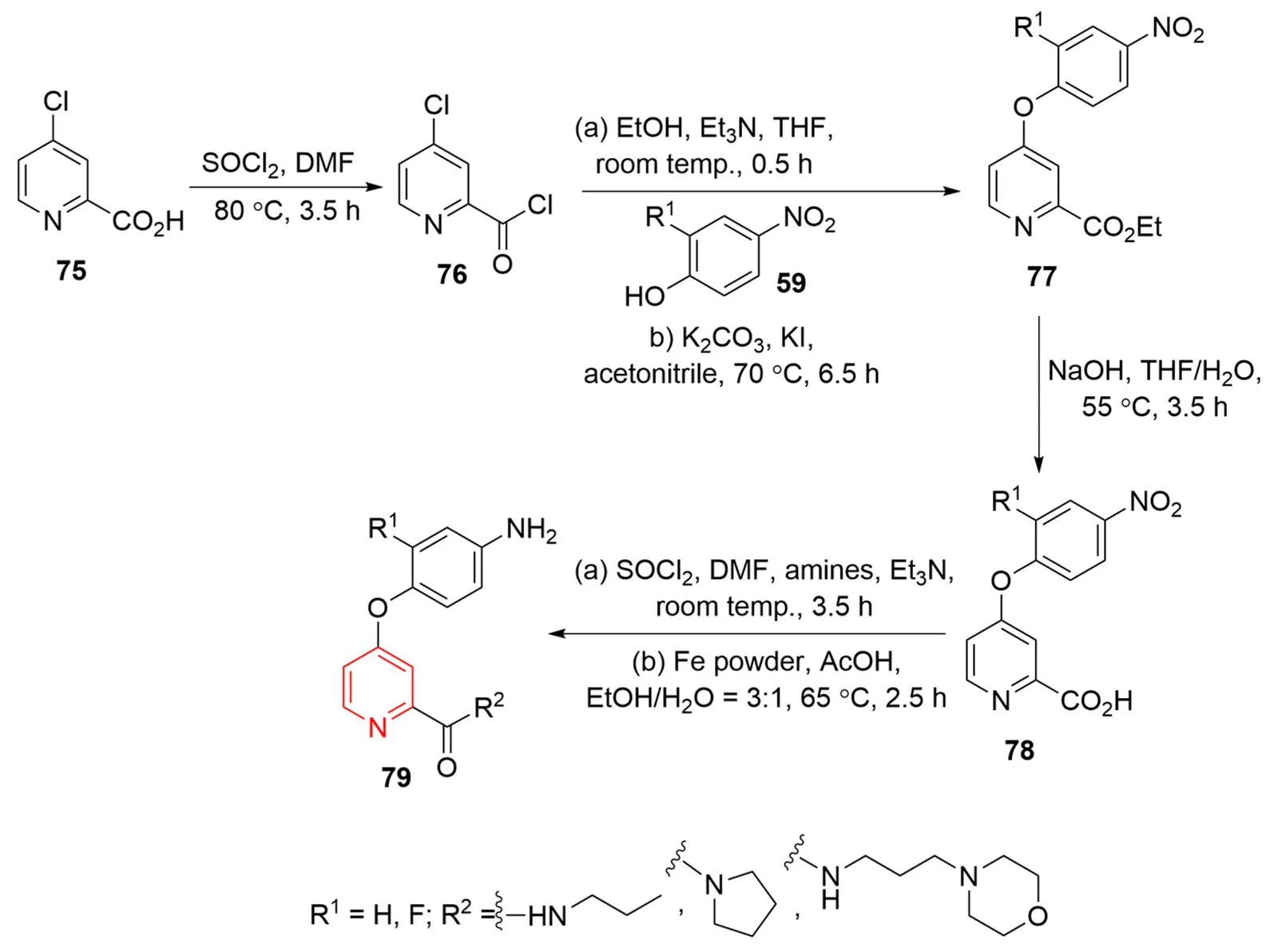
Synthetic route toward 4-(4-aminophenoxy)picolinamide intermediates **79**.

**Scheme 9:**
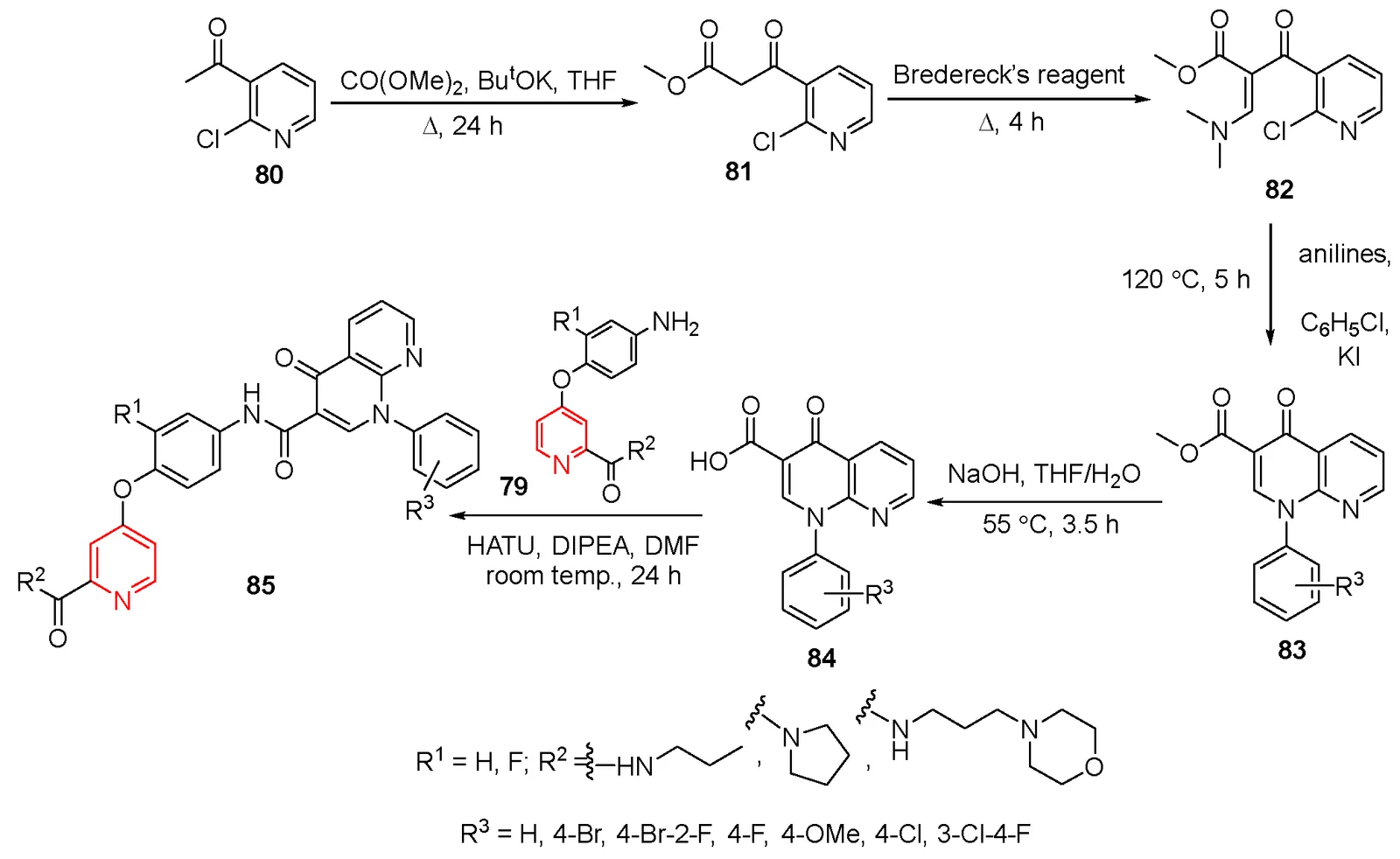
Synthetic route toward 4-(4-aminophenoxy)picolinamides **85**.

Building on the same Type II design philosophy, a series of 4-(4-aminophenoxy)picolinamides **85** was developed that incorporate a fluorinated phenyl linker and a 1,8-naphthyridinone-derived terminal pharmacophore (Scheme 8 and Scheme 9) [127]. The design retained key structural features of established Type II inhibitors while introducing side chains intended to improve physicochemical and cellular properties.

The lead compound, bearing a 3-morpholinopropylamino side chain and a 3-chloro-4-fluorophenyl terminal group, showed potent antiproliferative activity, yielding IC_50_ values of 0.26, 0.82, and 2.35 μM against A549, HeLa, and MCF7 cells, respectively. These values were superior to those of cabozantinib under the same experimental conditions [127].

Biochemical evaluation confirmed direct c-Met inhibition, with the most potent compound showing an IC_50_ of 46.5 compared to staurosporine at 80.4 nM. The lead compound also induced apoptosis and G_0_/G_1_-phase arrest in A549 cells. Docking and molecular dynamics analyses supported interactions with Met1160, Asp1222, Lys1110, and Phe1223, indicating that activity arose from coordinated engagement of the hinge region, catalytic region, and hydrophobic back pocket [127].

**Scheme 10:**
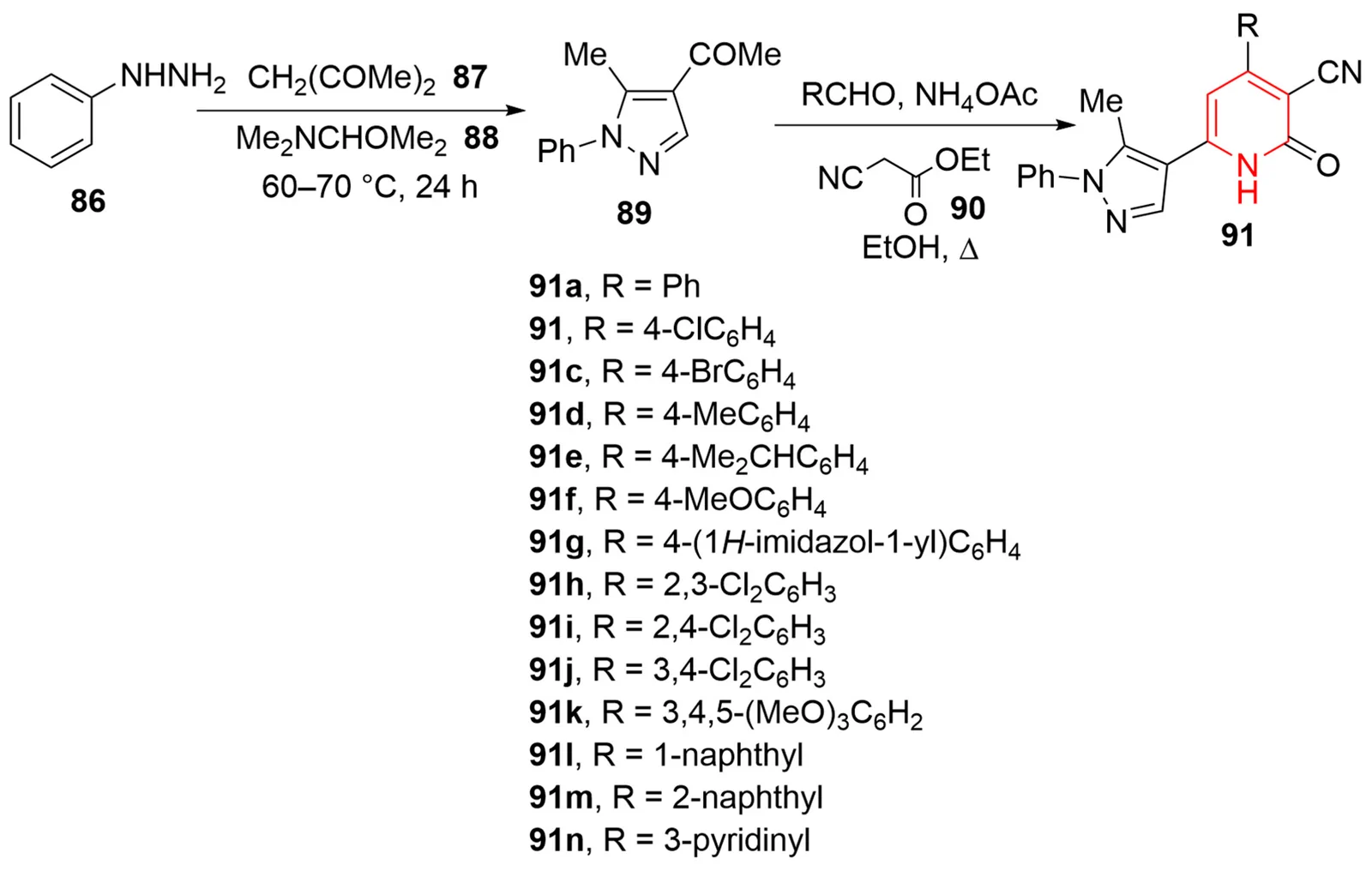
Synthetic route toward pyrazole-containing nicotinonitrile derivatives **91**.

The pyridine scaffold has also been incorporated into multitarget kinase inhibitors. In this context, nicotinonitrile derivatives **91**, which contain a pyrazole pharmacophore, were developed as dual c-Met/Pim-1 inhibitors (Scheme 10) [128].

Among the synthesized compounds, **91d** and **91h** showed the most balanced dual-target activity. Compound **91d** inhibited c-Met and Pim-1 with IC_50_ values of 151 and 110 nM, respectively, whereas **91h** showed corresponding values of 190 and 123 nM [128]. Both compounds inhibited proliferation in PC-3, HCT116, and MDA-MB-231 cancer cells, although **91h** exhibited greater cellular activity despite slightly weaker biochemical potency. This observation illustrates the frequent disconnect between enzyme inhibition and cellular efficacy.

Mechanistic studies revealed induction of apoptosis, modulation of Bax and Bcl-2 expression, activation of caspase-3, and S-phase arrest. Docking and molecular dynamics studies supported productive interactions with the c-Met and Pim-1 kinase domains [128]. Because direct biochemical inhibition of both targets was demonstrated, **91d** and **91h** can be regarded as experimentally validated dual c-Met/Pim-1 inhibitors.

Several recurring design features were linked to improved activity, including fluorinated aromatic substituents, 4-phenoxy linkers, cyclopropanecarboxamide motifs, and extended hydrogen-bonding pharmacophores such as imidazole carboxamides and naphthyridinones. Importantly, this scaffold class also illustrates the progression from selective c-Met inhibitors (**55**, **74**, **85**) to rationally designed multitarget agents (**91**), a theme that recurs throughout modern c-Met-directed drug discovery.

### 4.3. Quinoline-Based c-Met Inhibitors

Quinoline is a privileged nitrogen-containing fused heterocycle widely used in the design of receptor tyrosine kinase inhibitors. Its rigid, planar aromatic framework promotes favorable hydrophobic and π-π interactions within kinase-binding pockets, while the ring nitrogen can participate in hinge-region recognition by accepting hydrogen bonds. Owing to these properties, quinoline-based scaffolds have been widely employed in the development of Type II c-Met inhibitors and remain important platforms for designing both selective c-Met inhibitors and rationally engineered multitarget anticancer agents.

**Scheme 11:**
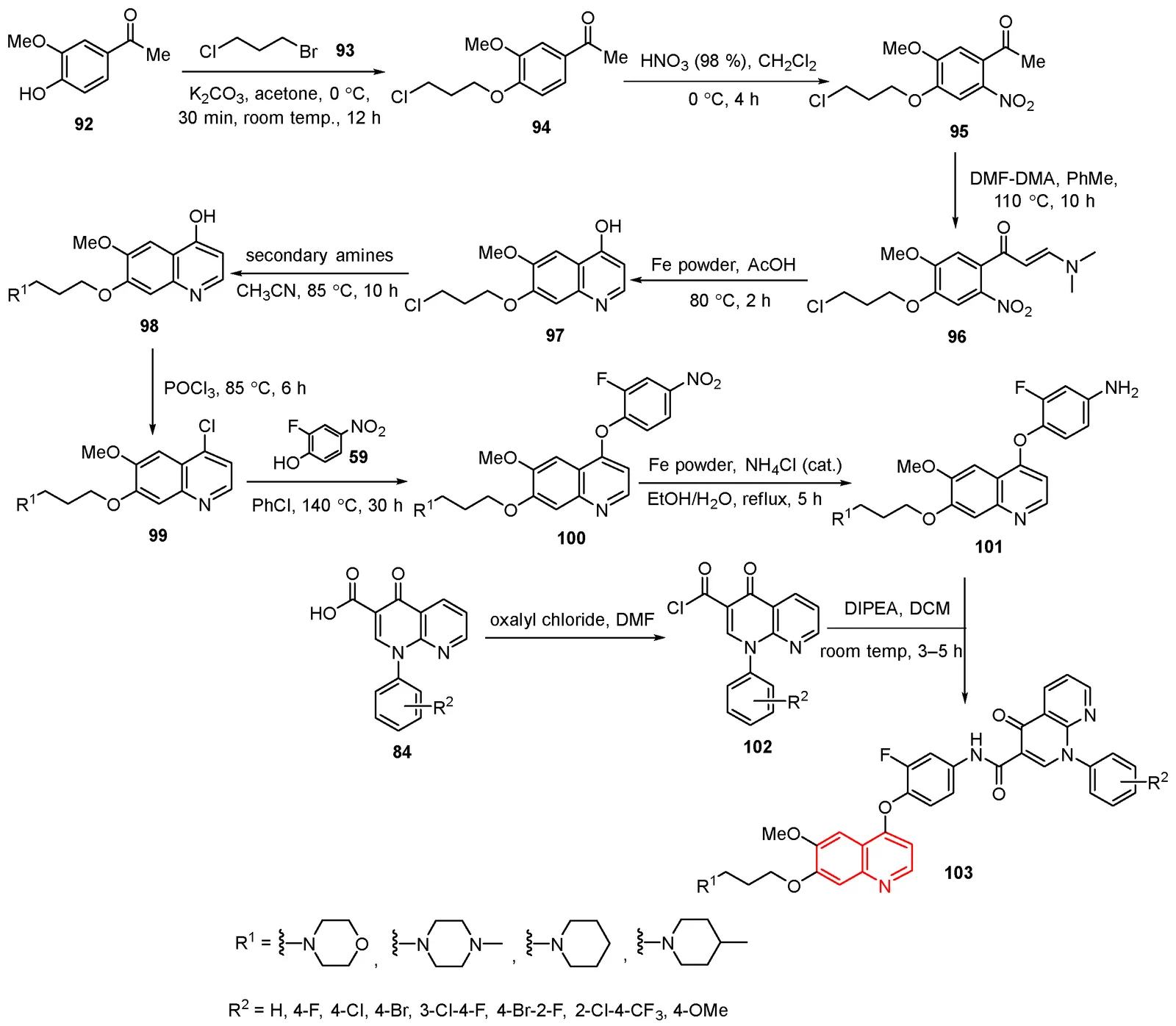
Synthetic route toward 4-phenoxyquinoline-1,8-naphthyridine hybrids **103**.

Guided by the pharmacophoric architecture of established Type II c-Met inhibitors, a series of 4-phenoxyquinoline derivatives was designed by incorporating a 1,8-naphthyridine pharmacophore **103** [20]. The design preserved three key features of successful Type II inhibitors: a heterocyclic hinge-binding motif, a 4-phenoxy linker capable of spanning the ATP-binding site and the hydrophobic back pocket, and an amide-containing hydrogen-bonding region. The target compounds were synthesized by constructing a functionalized quinoline core, installing the phenoxy linker, and coupling it with a substituted 1,8-naphthyridine fragment (Scheme 11) [20].

Several members of the series showed potent antiproliferative activity against HeLa, A549, and MCF7 cells. The lead compound, bearing a 4-methylpiperazinyl substituent and a 4-fluorophenyl terminal group, exhibited IC_50_ values of 0.21, 0.39, and 0.33 μM, respectively, and outperformed foretinib in the corresponding cellular assays. SAR analysis revealed that the morpholinyl and 4-methylpiperazinyl moieties are superior to piperidinyl or 4-methylpiperidinyl groups. This preference is likely due to enhanced hydrophilicity, which optimizes antiproliferative efficacy [20].

Mechanistic studies demonstrated concentration-dependent induction of apoptosis and G_1_-phase cell-cycle arrest. Importantly, direct kinase profiling revealed potent multitarget inhibition of c-Met, MEK1, and FLT3, with IC_50_ values of 11.77, 10.71, and 22.36 nM, respectively. This profile suggests that the observed antiproliferative activity likely results from simultaneous disruption of multiple oncogenic signaling networks rather than from selective c-Met inhibition alone.

Docking studies using the c-Met kinase structure (PDB ID: 3LQ8) predicted a binding mode closely resembling foretinib’s. The quinoline nucleus occupied the ATP-binding region, while the extended phenoxy-linked pharmacophore projected into the hydrophobic back pocket characteristic of Type II inhibitors. Additional modeling against MEK1 and FLT3 supported the experimentally observed multikinase profile [20].

A complementary strategy explored partially saturated quinoline analogs by synthesizing 1,4,5,6,7,8-hexahydroquinoline-3-carbonitrile derivatives **109** [129]. In contrast to the elongated architecture of the 4-phenoxyquinoline series, these compounds employed a more compact scaffold incorporating a nitrile group, a hydrophobic aromatic region, and strategically positioned substituents to modulate polarity and target interactions.

**Scheme 12:**
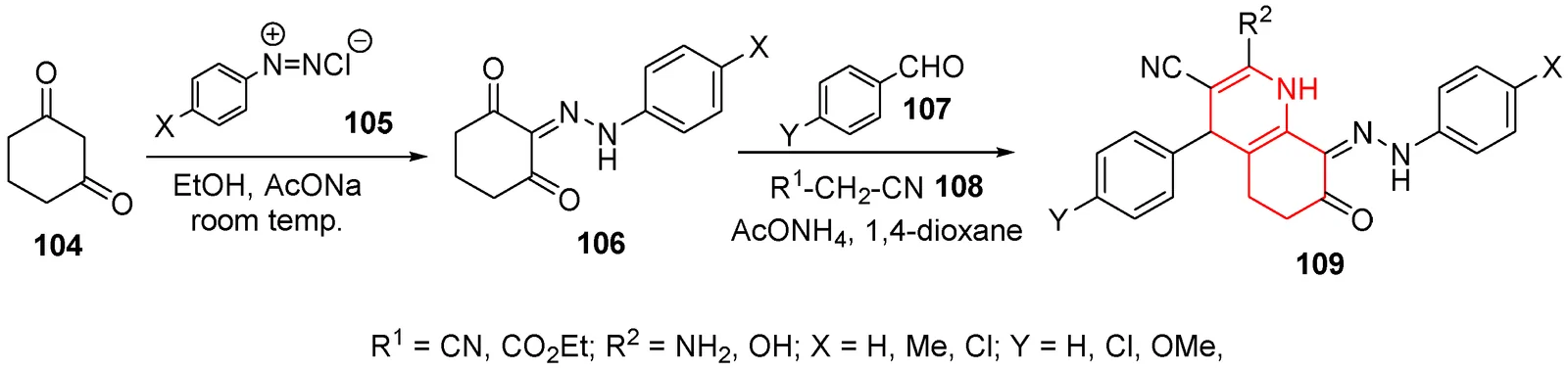
Synthetic route toward 1,4,5,6,7,8-hexahydroquinoline-3-carbonitriles **109**.

The target compounds were prepared via a multicomponent cyclocondensation sequence that used arylhydrazone intermediates, substituted aromatic aldehydes, and activated nitrile-containing building blocks (Scheme 12) [129].

The most active derivative, bearing a dichlorophenyl group and a hydroxyl substituent, showed broad antiproliferative activity across a panel of cancer cell lines, including A549, H460, HT-29, MKN-45, U87MG, and SMMC-7721, with IC_50_ values ranging from 0.23 to 0.42 μM [129]. Although foretinib remained more potent in some models, the lead hexahydroquinoline analog matched or exceeded its activity in others, indicating a broad and balanced antiproliferative profile.

Biochemical evaluation confirmed direct c-Met inhibition, with the lead compound showing an IC_50_ of 2.40 nM, compared with 1.16 nM for foretinib [129]. The relatively small difference in enzyme potency contrasted with larger variations in cellular activity, highlighting the importance of factors beyond target affinity, such as cellular uptake, target engagement, and pharmacokinetic behavior.

Preliminary SAR analysis identified the dichloro substitution pattern as a key contributor to activity, likely by enhancing hydrophobic interactions within the kinase binding site. The hydroxyl substituent may further contribute through favorable hydrogen-bonding interactions and improved polarity balance. However, broader analog exploration and kinase-selectivity profiling will be necessary to establish the generality of these observations and to determine whether the scaffold exhibits selectivity for c-Met or an unrecognized multikinase profile.

Quinoline-based inhibitors demonstrate the scaffold’s versatility for both direct c-Met inhibition and rational multitarget kinase design. Incorporating extended hydrogen-bonding motifs, fluorinated aromatic groups, and polar cyclic amines consistently enhanced biological activity and enabled effective exploitation of the Type II c-Met inhibitor pharmacophore.

### 4.4. Pyrazole-Based c-Met Inhibitors

Pyrazole is a versatile five-membered nitrogen-containing heterocycle widely used in medicinal chemistry for its favorable electronic properties, synthetic accessibility, and capacity to engage in hydrogen-bonding and aromatic interactions within kinase ATP-binding sites. The scaffold readily accommodates structural diversification through substitution at both nitrogen and carbon positions, making it particularly attractive for molecular hybridization strategies to generate multitarget anticancer agents. Although pyrazole derivatives have been less extensively explored as c-Met inhibitors than pyridine-, quinoline-, or pyrimidine-based scaffolds, several representatives have demonstrated promising c-Met-associated antitumor activity.

**Scheme 13:**
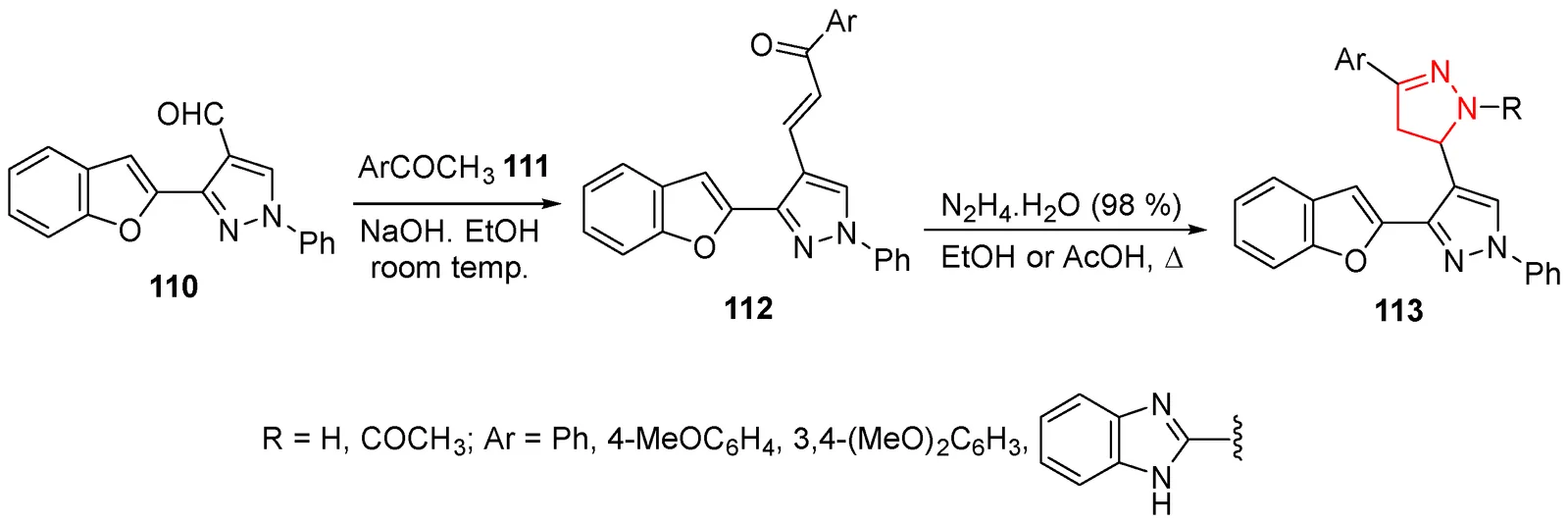
Synthetic pathway for benzofuran–pyrazole hybrids **113**.

A series of benzofuran–pyrazole hybrids **113** was generated by molecular hybridization of benzofuran, pyrazole, and terminal aromatic or heteroaromatic pharmacophores [130]. The synthetic route involved preparation of 3-(benzofuran-2-yl)-1-phenyl-1*H*-pyrazole-4-carbaldehyde **110**, followed by Claisen–Schmidt condensation with substituted acetophenones or 2-acetylbenzimidazole **111** to afford α,β-unsaturated ketones **112**. Subsequent cyclization with hydrazine hydrate provided the target pyrazoline-containing hybrids **113** (Scheme 13) [130].

Initial biological evaluation using the NCI-60 cancer cell line screening panel identified several active derivatives. The most promising compound, which incorporates a benzimidazole moiety, showed a GI_50_ of 1.84 μM against UACC-62 melanoma cells. Enzymatic profiling revealed broad multikinase inhibitory activity against mutant B-Raf^V600E^, c-Met, Pim-1, EGFR, and VEGFR-2. The strongest inhibition was observed against B-Raf^V600E^, c-Met, and Pim-1, indicating that the antiproliferative activity likely results from coordinated modulation of multiple oncogenic pathways rather than selective c-Met inhibition alone [130].

Flow cytometric analysis demonstrated G_0_/G_1_-phase arrest in MCF7 breast cancer cells, while molecular docking studies indicated favorable accommodation of the lead compound within the ATP-binding pockets of c-Met and other experimentally validated kinase targets. The benzimidazole-containing analog consistently outperformed other members of the series, suggesting that the expanded aromatic surface and additional hydrogen-bonding capability of this bicyclic heterocycle contributed to improved kinase recognition and cellular activity.

**Scheme 14:**
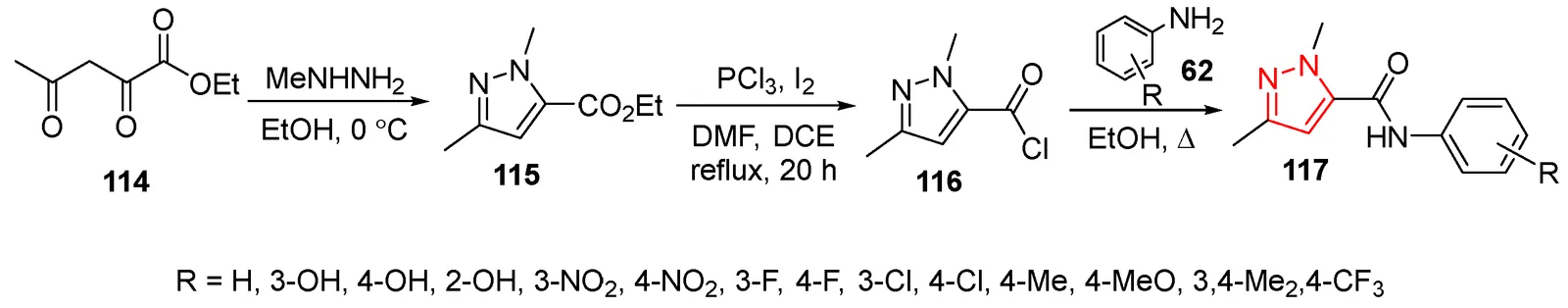
Synthetic pathway for 1H-pyrazole-5-carboxamides **117**.

A second application of the pyrazole scaffold was reported by Prathyusha and Deepti, who synthesized a series of 1*H*-pyrazole-5-carboxamides **117** and evaluated their antiproliferative activity against lung, colorectal, prostate, cervical, breast, and melanoma cancer models [131]. The target molecules were prepared by constructing a pyrazole ester core, converting it to the corresponding acid chloride **116**, and coupling it with substituted aromatic amines (Scheme 14).

Among the synthesized compounds, the 3-fluorophenyl-substituted derivative showed the most favorable biological profile. It inhibited A549, HT-29, DU145, SiHa, MCF7, and B16F10 cells, with IC_50_ values ranging from 5.87 to 23.11 μM. Although these activities were modest compared with doxorubicin, the compound showed lower toxicity toward nonmalignant fibroblasts, suggesting a potentially favorable preliminary safety profile [131].

Mechanistic assignment in this study was less definitive than in the benzofuran–pyrazole series. Docking studies using c-Met and JAK1 structures suggested favorable accommodation within the ATP-binding sites of both kinases, leading to the proposal of dual c-Met/JAK1 activity. However, no direct kinase inhibition, biophysical binding measurements, or cellular target engagement studies were reported [131]. Consequently, these compounds should be regarded as putative c-Met/JAK1-directed agents rather than validated kinase inhibitors.

Within the limited SAR, the 3-fluorophenyl substituent was associated with the most favorable antiproliferative activity, suggesting that fluorine substitution may beneficially influence electronic properties, hydrophobic interactions, and molecular recognition. Additional analog expansion and direct target-validation studies will be required to establish the precise role of the pyrazole scaffold in c-Met inhibition.

Overall, pyrazole-based compounds underscore the growing importance of rational polypharmacology in c-Met-directed drug discovery. Although few highly selective c-Met inhibitors based on this scaffold have been reported, pyrazole-containing architectures remain a versatile platform for developing multitarget anticancer agents that can address pathway redundancy and therapeutic resistance.

### 4.5. Imidazole-Based c-Met Inhibitors

Imidazole is a privileged five-membered nitrogen-containing heterocycle widely used in medicinal chemistry for its aromaticity, favorable hydrogen-bonding properties, and synthetic versatility. Depending on its substitution pattern and tautomeric state, the imidazole ring can act as both a hydrogen-bond donor and acceptor, supporting productive interactions within kinase ATP-binding sites. Although relatively few imidazole-containing compounds have been developed as selective c-Met inhibitors, imidazole-based molecular hybrids have shown promise as multitarget anticancer agents that simultaneously modulate c-Met and complementary oncogenic pathways.

**Scheme 15:**
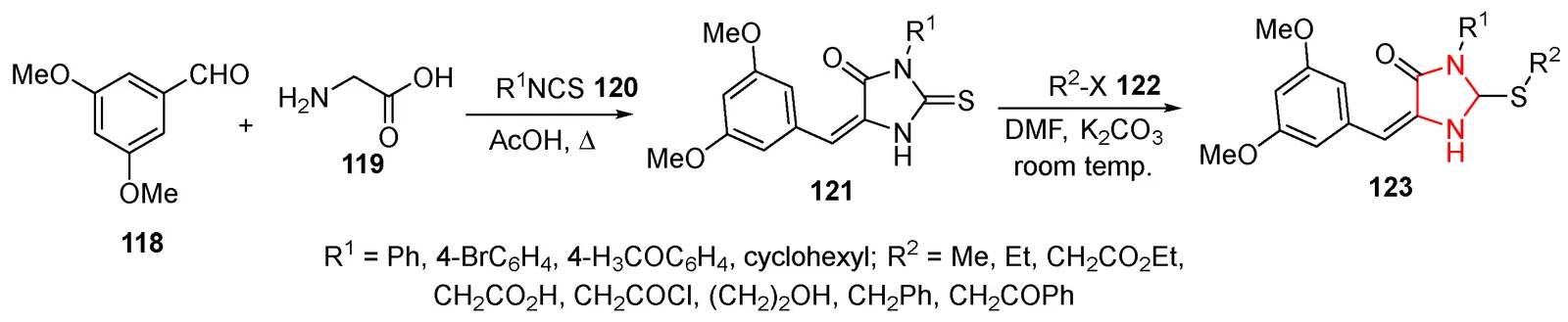
Synthetic route toward 2-(alkylthio)-3-aryl-5-(3,4-dimethoxybenzylidene)-3,5-dihydro-4*H*-imidazol-4-ones **123**.

A series of 2-(alkylthio)-3-aryl-5-(3,4-dimethoxybenzylidene)-3,5-dihydro-4*H*-imidazol-4-ones **123** was reported as potential anticancer agents for colorectal cancer treatment [132]. The target compounds were synthesized via a one-pot multicomponent reaction of alkyl or aryl isothiocyanates, glycine, and 3,4-dimethoxybenzaldehyde to afford 2-thioxoimidazolidin-4-one intermediates **121**, which were then S-alkylated with appropriate alkylating agents **122** to furnish the final imidazol-4-one derivatives **123** (Scheme 15).

Biological evaluation against HT-29 colorectal cancer cells identified the derivative with R^1^ = Ph and R^2^ = CH_2_COPh as the most active compound in the series. The lead compound exhibited an IC_50_ of 2.197 μg/mL, compared with 3.992 μg/mL for doxorubicin under the reported assay conditions [132]. Although this comparison suggests strong antiproliferative activity, direct potency comparisons should be interpreted cautiously because the results were reported as mass concentrations rather than molar concentrations.

Importantly, enzymatic profiling demonstrated that the lead compound was a multikinase inhibitor. The compound inhibited VEGFR-2, c-Met, and Pim-1 with IC_50_ values of 0.081, 0.160, and 0.291 μg/mL, respectively [132]. Relative to the reference inhibitors used in the study, the compound showed greater activity against VEGFR-2 and Pim-1 but lower potency against c-Met than foretinib. These results establish direct inhibition of c-Met and demonstrate a broader kinase-inhibition profile.

The combination of c-Met, VEGFR-2, and Pim-1 inhibition may provide complementary anticancer effects by simultaneously targeting tumor-cell proliferation, angiogenesis, survival signaling, and resistance pathways. Consistent with this multitarget profile, the lead compound induced G_0_/G_1_ arrest and apoptosis in HT-29 cells, with increased expression of Bax, caspase-3, and caspase-9 and reduced Bcl-2 levels [132]. However, the available data do not establish which kinase contributes most strongly to the observed cellular response.

Docking studies using the crystal structures of c-Met, VEGFR-2, and Pim-1 (PDB IDs: 6SDC, 3WZE, and 3A99, respectively) predicted favorable accommodation of the lead compound within the ATP-binding sites of all three kinases. Molecular dynamics simulations further supported the stability of the proposed protein–ligand complexes [132]. Because direct biochemical inhibition data were available, these computational studies should be viewed as structural rationalizations of experimentally observed activity rather than primary evidence of target engagement.

Overall, imidazole-based compounds remain an underexplored class of c-Met-associated inhibitors, yet their structural flexibility and compatibility with multitarget inhibitor design make them attractive candidates for further development.

### 4.6. 1,3-Thiazole-Based c-Met Inhibitors

1,3-Thiazole is a privileged sulfur- and nitrogen-containing five-membered heterocycle widely used in medicinal chemistry for its favorable electronic properties, synthetic accessibility, and ability to engage in hydrogen-bonding, hydrophobic, and aromatic interactions within protein-binding sites. In kinase inhibitor design, the thiazole nitrogen can serve as a hydrogen-bond acceptor in the hinge region, while the aromatic ring contributes to hydrophobic and π-stacking interactions. These properties have made the 1,3-thiazole scaffold an attractive platform for developing ATP-competitive c-Met inhibitors.

**Figure 9:**
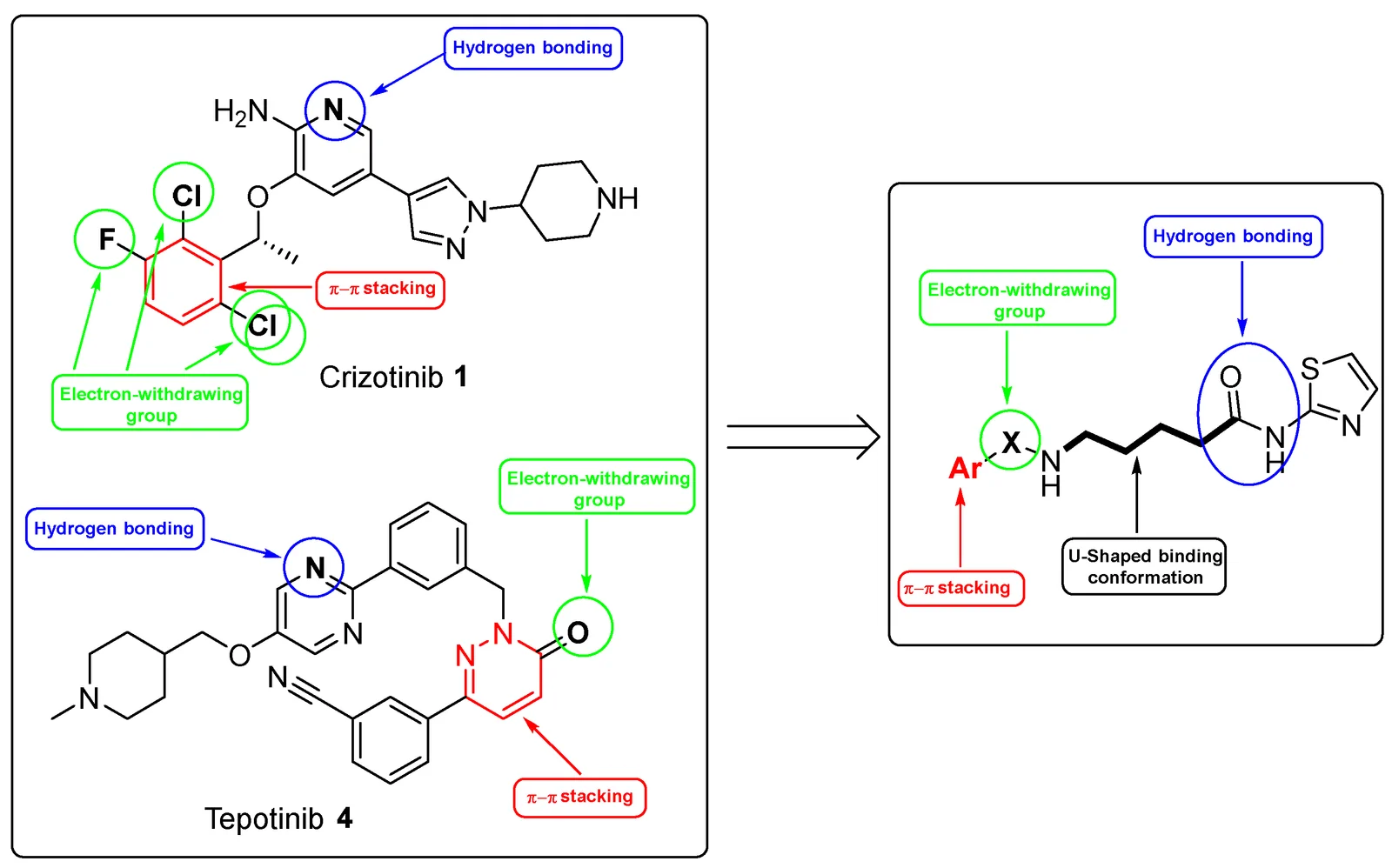
Structure-based design strategy for 1,3-thiazole derivatives **131** based on the c-Met-binding features of crizotinib **1** and tepotinib **4**.

Wang and co-workers used a structure-based design strategy to develop a series of 1,3-thiazole derivatives **131** as potential c-Met inhibitors [133]. The design was guided by analysis of the binding modes of crizotinib and tepotinib in the c-Met kinase domain, which identified three key features associated with potent inhibition: (i) a heterocyclic nitrogen capable of interacting with the hinge-region residue Met1160, (ii) an aromatic region positioned to interact with Tyr1230, and (iii) a U-shaped, folded conformation characteristic of Type I c-Met inhibitors. The 1,3-thiazole nucleus was selected as the hinge-binding pharmacophore, and a flexible hexanamide linker connected the thiazole core to variable terminal aromatic amide or sulfonamide groups (Figure 9).

**Scheme 16:**
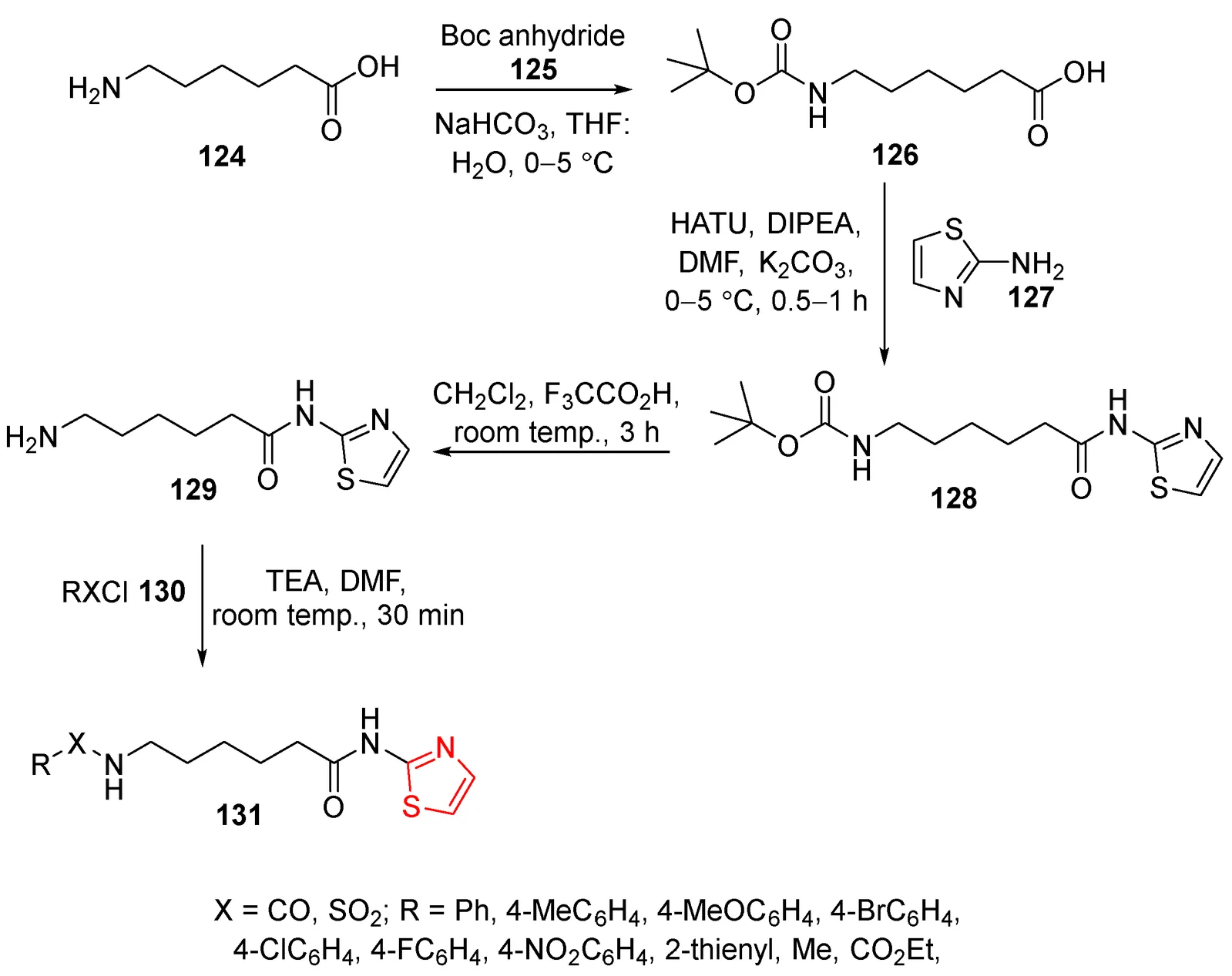
Synthetic route toward 1,3-thiazoles **131**.

The target compounds were synthesized from 6-aminohexanoic acid **124** via sequential Boc protection, coupling with 2-amino-1,3-thiazole **127**, deprotection, and a final reaction with substituted acyl or sulfonyl chlorides **130** to afford the desired amide- and sulfonamide-containing derivatives **131** (Scheme 16) [133]. This modular route enabled efficient late-stage diversification of the terminal aromatic region while preserving the thiazole-containing hinge-binding element and the flexible linker.

Biochemical evaluation identified two *p*-tolyl-substituted derivatives as the most potent in the series. The *p*-tolylsulfonamide analog inhibited c-Met with an IC_50_ of 0.38 nM, whereas the corresponding *p*-tolylamide derivative had an IC_50_ of 0.40 nM. Under the same assay conditions, crizotinib inhibited c-Met with an IC_50_ of 3.4 nM. These results demonstrate that the optimized thiazole derivatives achieved subnanomolar c-Met inhibition and were approximately ninefold more potent than crizotinib in the reported biochemical assay [133].

Despite their exceptional enzymatic potency, the compounds showed only moderate antiproliferative activity in cellular assays. The *p*-tolylamide derivative inhibited HT-29 colorectal and MDA-MB-231 triple-negative breast cancer cells with IC_50_ values of 7.89 and 5.78 μM, respectively, whereas the corresponding sulfonamide analog produced IC_50_ values of 7.25 and 27.25 μM. In comparison, crizotinib yielded IC_50_ values of 3.23 and 4.67 μM under the same conditions [133].

The pronounced discrepancy between biochemical and cellular potency suggests that factors beyond target affinity substantially contribute to biological performance. Differences in membrane permeability, intracellular exposure, target engagement, metabolic stability, efflux susceptibility, and tumor dependence on c-Met signaling may all influence the observed cellular activity. Interestingly, the amide derivative displayed a more balanced cellular profile than the corresponding sulfonamide, despite nearly identical enzyme potency, indicating that linker chemistry can affect cellular behavior independently of c-Met affinity.

Molecular docking studies using the c-Met-crizotinib co-crystal structure (PDB ID: 2WGJ) supported the intended design strategy. The thiazole nitrogen was positioned to replicate the hinge interaction with Met1160, while the aromatic terminal region was oriented toward Tyr1230. The flexible six-carbon linker allowed the compounds to adopt the U-shaped, folded conformation characteristic of Type I c-Met inhibitors [133].

Because direct biochemical inhibition was demonstrated and the predicted binding mode aligns with established Type I pharmacophores, compound **131** is an experimentally validated ATP-competitive c-Met inhibitor. However, the substantial enzyme-to-cell potency gap suggests that future optimization should focus not only on further improving target affinity but also on properties that govern cellular exposure, target engagement, and overall pharmacological performance.

These results demonstrate that the 1,3-thiazole scaffold can support potent ATP-competitive inhibition of c-Met, although optimizing cellular exposure and target engagement remains necessary to translate exceptional enzyme potency into robust cellular activity.

### 4.7. Pyrimidine-Based c-Met Inhibitors

Pyrimidines are among the most widely used nitrogen-containing heterocycles in kinase inhibitor discovery because of their favorable electronic properties, synthetic versatility, and ability to form productive hydrogen-bonding interactions with kinase hinge-region residues. As a diazine heterocycle structurally related to the purine nucleus of ATP, pyrimidine serves as an effective ATP-mimetic scaffold. Its two ring nitrogens provide well-defined hydrogen-bond-acceptor sites, while multiple substitution positions allow for precise tuning of molecular geometry, lipophilicity, polarity, and kinase selectivity. Consequently, pyrimidine-containing architectures have become valuable platforms for scaffold hopping and for developing ATP-competitive c-Met inhibitors with improved potency and drug-like properties.

**Figure 10:**
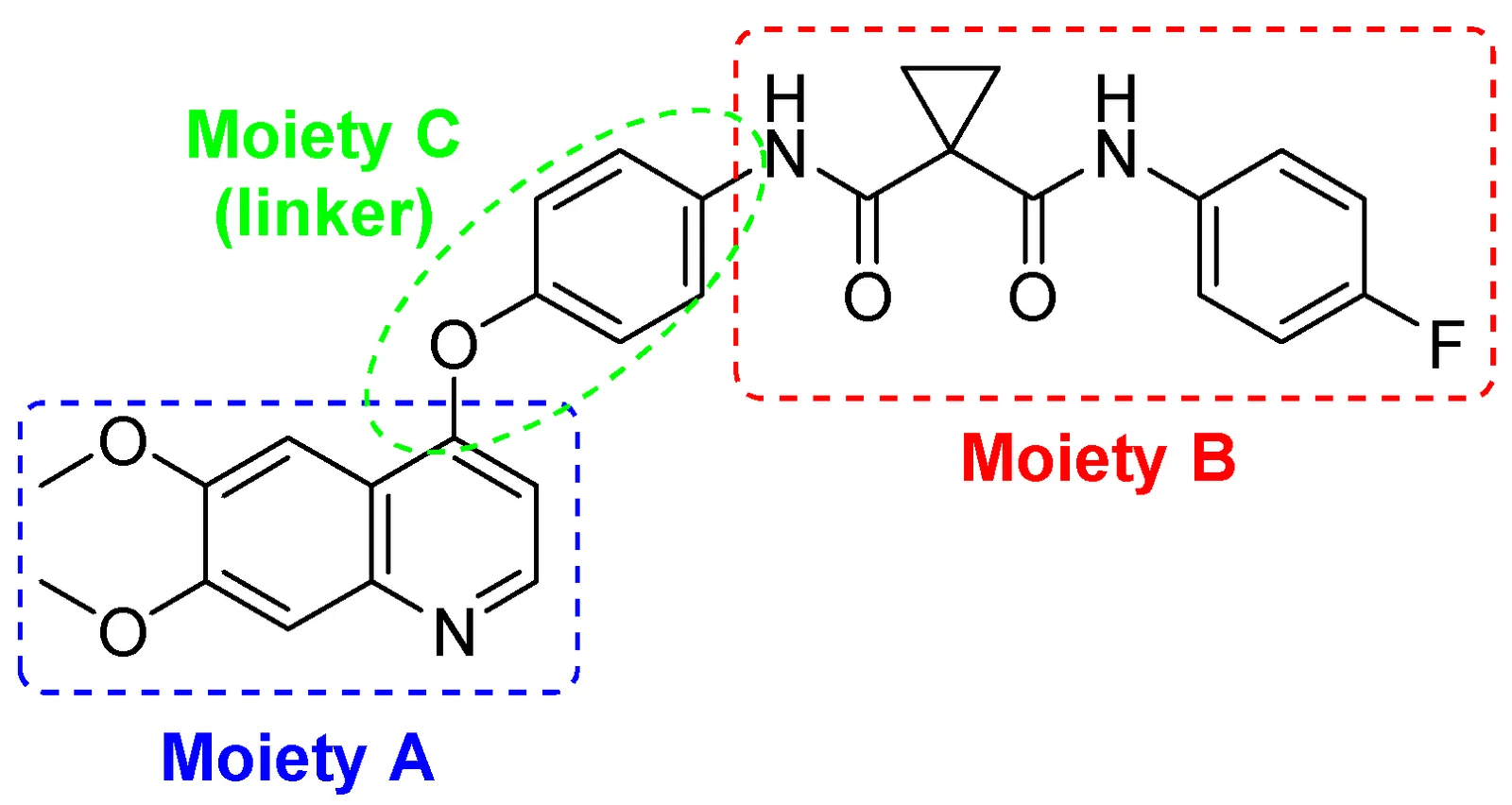
Pharmacophoric features of cabozantinib utilized for the design of N-phenylpyrimidin-2-amines **137**.

Inspired by the binding architecture and clinical success of cabozantinib **10**, Liu and co-workers used a pharmacophore-guided scaffold-hopping strategy (Figure 10) to design a series of N-phenylpyrimidin-2-amines **137** as potential c-Met inhibitors [134]. The design retained key pharmacophoric elements associated with Type II c-Met inhibition, including a hinge-binding heterocycle, a central hydrogen-bonding region, and a hydrophobic aromatic domain that can occupy the back pocket exposed in the inactive DFG-out kinase conformation. The quinoline-based hinge-binding region of cabozantinib was replaced with an N-phenylpyrimidin-2-amine scaffold, while preserving the overall spatial arrangement of the remaining pharmacophoric features.

**Scheme 17:**
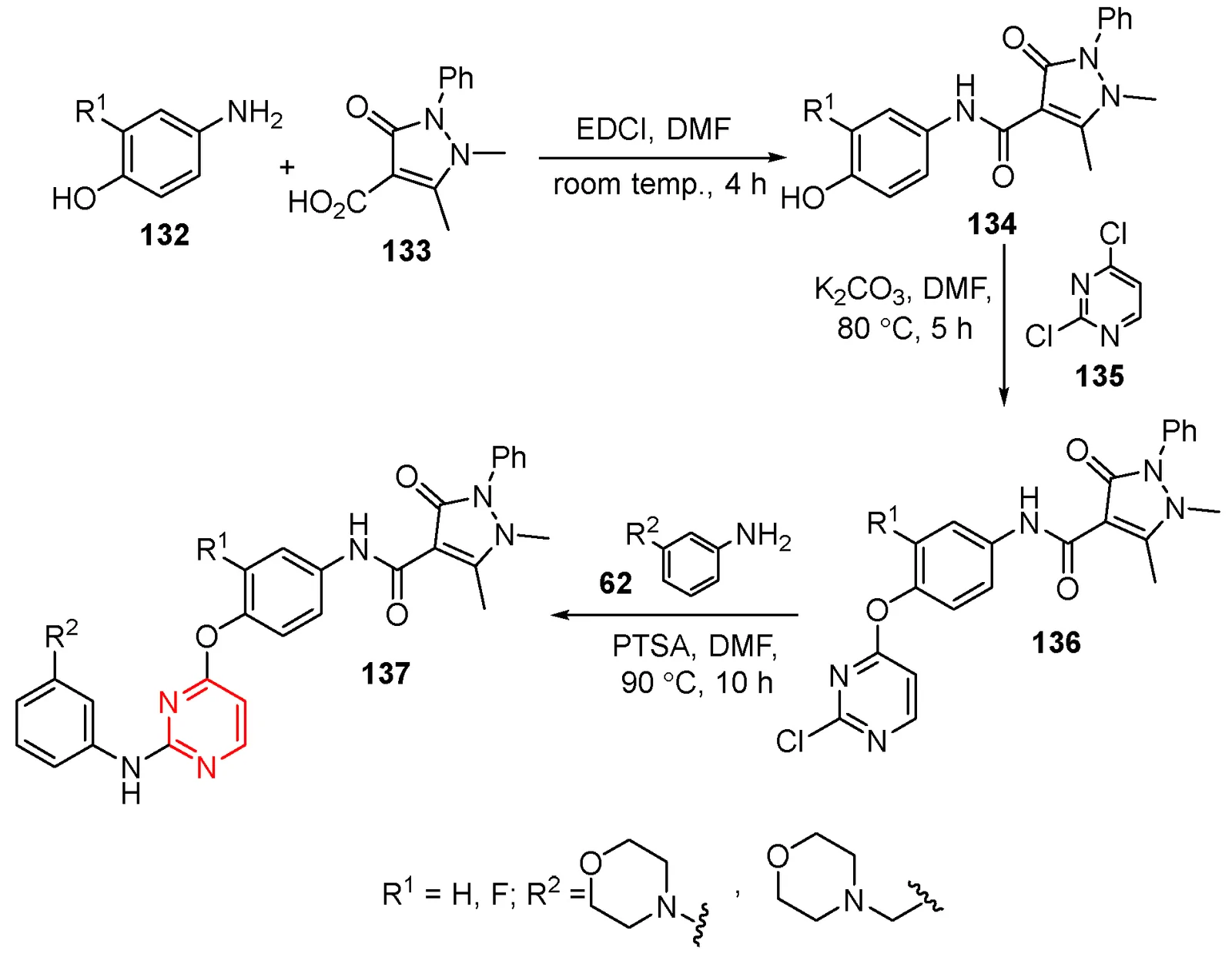
Synthetic route towards *N*-phenylpyrimidin-2-amines **137**.

The target compounds **137** were synthesized via a modular route that involved EDCI-mediated coupling of substituted 4-aminophenols **132** with pyrazole-4-carboxylic acid derivatives **133**, followed by nucleophilic substitution with 2,4-dichloropyrimidine **135** and final amination with substituted anilines **62** (Scheme 17) [134]. This strategy enabled independent modification of the aminophenoxy region, the pyrazole carboxamide motif, the pyrimidine core, and the terminal aniline substituent.

Among the synthesized compounds, the derivative with R^1^ = H and R^2^ = 4-morpholinyl emerged as the lead analog. Biochemical evaluation showed potent direct c-Met inhibition, with an IC_50_ of 15 nM, compared with 34 nM for cabozantinib under the reported assay conditions [134]. These results support the scaffold-hopping design strategy, demonstrating that replacing the quinoline hinge-binding region with a pyrimidine scaffold preserved and modestly improved c-Met inhibitory activity.

The lead compound also showed consistently strong antiproliferative activity across multiple cancer cell models. IC_50_ values of 1.01, 1.34, 1.34, 0.53, and 1.37 μM were reported against Panc-1, HepG2, PC-3, Caki-1, and HCT116 cells, respectively, compared with 5.13, 3.05, 3.68, 1.66, and 3.78 μM for cabozantinib under the same experimental conditions [134]. The compound therefore demonstrated improved cellular activity across all evaluated models, with the greatest enhancement observed in Panc-1 cells and the strongest absolute potency against Caki-1 renal cancer cells.

Importantly, the improved cellular activity was accompanied by favorable reported pharmacokinetic properties. Acute toxicity studies in mice showed no significant mortality at doses of 600–1200 mg/kg [134]. Although these findings suggest low acute toxicity under the reported conditions, they should not be interpreted as evidence of comprehensive safety, as chronic toxicity, organ-specific toxicity, and therapeutic-exposure margins were not evaluated.

Overall, the study demonstrates that pyrimidine can successfully replace quinoline as a hinge-binding scaffold while preserving the pharmacophoric features required for potent Type II c-Met inhibition. The lead compound’s favorable biochemical and cellular activities support further investigation of N-phenylpyrimidin-2-amines as promising c-Met-directed anticancer agents.

### 4.8. Quinazoline-Based c-Met Inhibitors

Quinazoline and quinazolinone are privileged bicyclic nitrogen-containing heterocycles that have played central roles in the development of receptor tyrosine kinase inhibitors. Numerous clinically important anticancer drugs, particularly EGFR inhibitors, contain a quinazoline nucleus that serves as an effective hinge-binding pharmacophore via hydrogen bonds with conserved kinase residues. The rigid fused aromatic framework also supports favorable hydrophobic and π-mediated interactions while accommodating extensive structural diversification.

**Figure 11:**
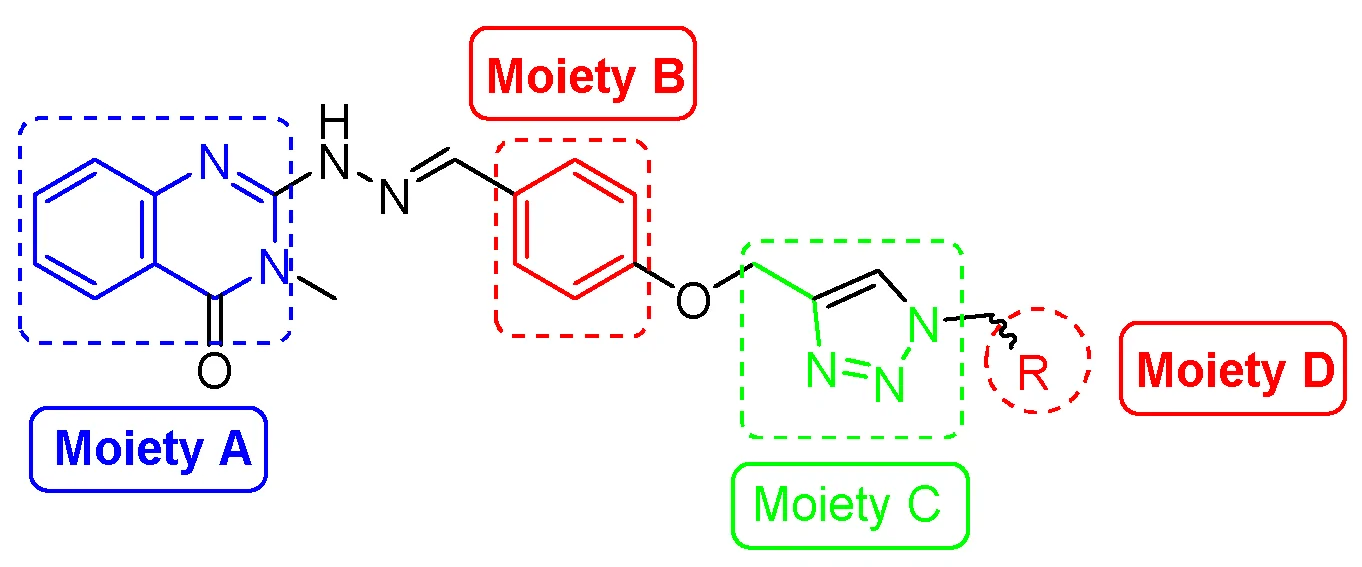
Pharmacophore-guided design strategy of quinazolinone hydrazones **147**.

Inspired by the pharmacophoric features of the multikinase inhibitors cabozantinib **10** and BMS-777607 **16**, a series of quinazolinone hydrazones **147** was designed using a molecular hybridization strategy (Figure 11) [135]. The design integrated a quinazolinone core as the heterocyclic recognition element, hydrophobic aromatic substituents, and a hydrazone linker intended to reproduce key features associated with kinase inhibition while enabling systematic variation of the terminal aromatic region.

**Scheme 18:**
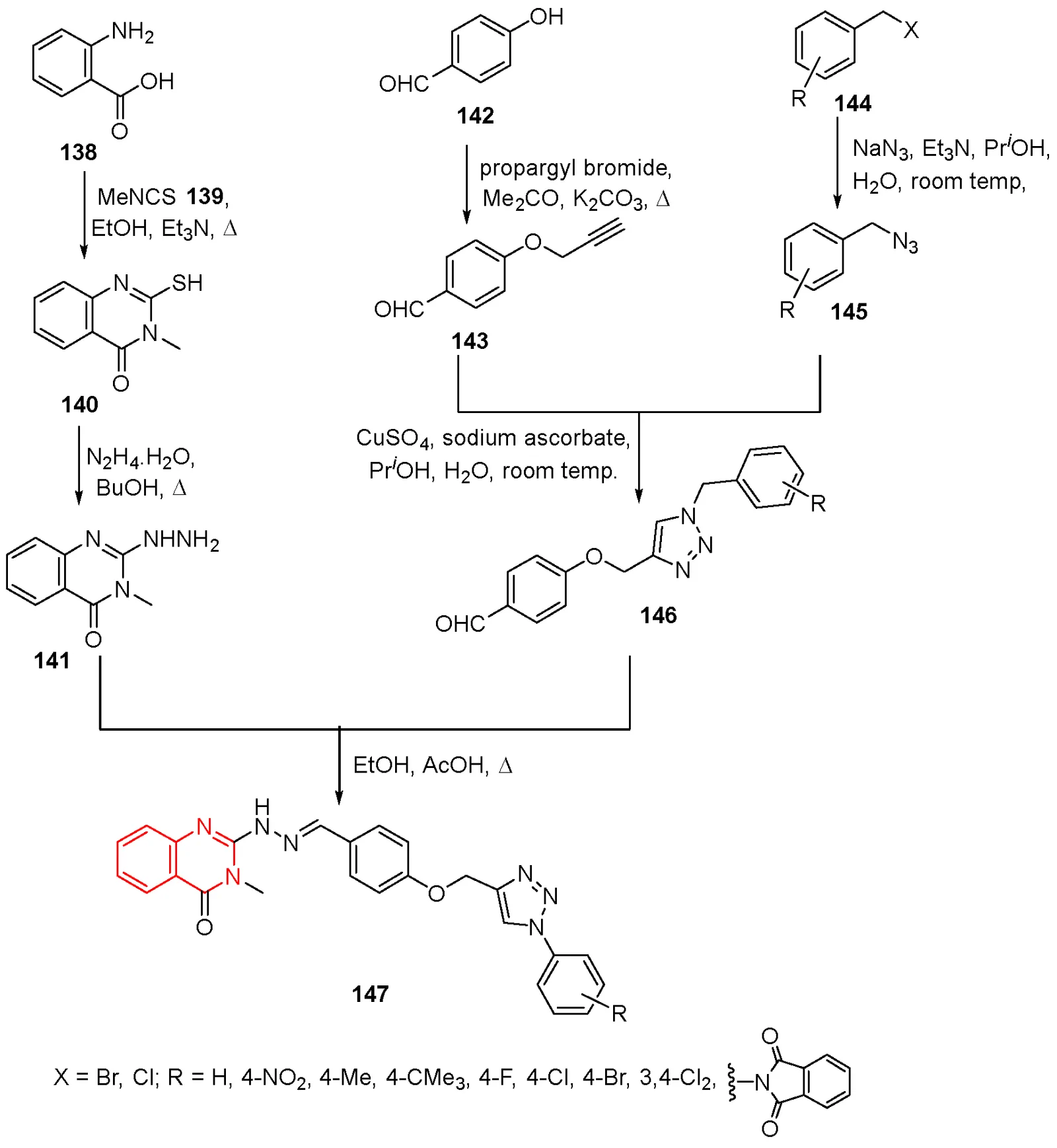
Synthetic route towards quinazolinone hydrazones **147**.

The target compounds **147** were synthesized from anthranilic acid **138** by constructing a hydrazine-containing quinazolinone intermediate **141**, followed by condensation with substituted benzaldehydes **146**, affording the final quinazolinone hydrazones (Scheme 18) [135]. The final condensation step provided a convenient route to diversify the terminal aromatic substituent while maintaining the quinazolinone scaffold and the hydrazone linker.

The synthesized compounds were evaluated against EBC-1 and A549 lung cancer cells, HT-29 colorectal cancer cells, and U-87MG glioblastoma cells. Among the series, the 4-bromophenyl derivative was the most active, with IC_50_ values of 8.6, 64.9, 65.2, and 24.6 μM against EBC-1, A549, HT-29, and U-87MG cells, respectively [135]. Although it was the lead compound in the series, its activity was substantially weaker than that of cabozantinib under the same experimental conditions, indicating only modest antiproliferative potency.

Biochemical studies demonstrated concentration-dependent inhibition of c-Met, with 37.1%, 53.0%, and 66.3% inhibition at 10, 25, and 50 μM, respectively [135]. These results support measurable c-Met-modulating activity but do not indicate high-affinity inhibition of c-Met. Because a full concentration–response curve and a biochemical IC_50_ were not reported, the compound’s true potency against c-Met cannot be accurately compared with that of established c-Met inhibitors.

Kinase profiling further revealed activity against VEGFR1/FLT1, VEGFR3/FLT4, ALK, AXL, and FGFR1, suggesting that the lead compound’s biological activity stems from a multitarget mechanism rather than selective c-Met inhibition [135]. Such a profile may be advantageous for addressing pathway redundancy but also underscores the need for improved characterization of kinase selectivity.

Cellular studies demonstrated apoptotic effects in EBC-1 cells, as evidenced by Hoechst 33258 staining and associated nuclear morphological changes [135]. The greater sensitivity of EBC-1 cells compared with other cancer models suggests dependence on the targeted signaling pathways. However, the available data do not establish whether apoptosis results primarily from c-Met modulation or from simultaneous inhibition of multiple kinases.

Molecular docking with the c-Met kinase structure (PDB ID: 3LQ8) predicted favorable accommodation of the lead compound in the ATP-binding region and provided a structural rationale for the observed c-Met-associated activity [135]. However, the docking results should be considered supportive rather than confirmatory, as direct target engagement and detailed kinetic studies were not reported.

Overall, the quinazolinone hydrazones **147** constitute a multitarget kinase-inhibitor scaffold with measurable c-Met-associated activity. Although the series did not match the potency of established c-Met inhibitors, it demonstrates that quinazolinone-based molecular hybridization can yield compounds that simultaneously modulate multiple receptor tyrosine kinases and may serve as a useful starting point for further optimization.

### 4.9. Quinoxaline-Based c-Met Inhibitors

Quinoxaline is a bicyclic nitrogen-containing heterocycle that has attracted considerable interest in kinase inhibitor discovery for its rigid, planar structure and favorable electronic properties. The two ring nitrogens can participate in kinase recognition, while the fused aromatic framework supports hydrophobic and π-mediated interactions within ATP-binding pockets. These features have made quinoxaline a useful scaffold for developing receptor tyrosine kinase inhibitors, including compounds that modulate c-Met-associated signaling pathways.

Chandrasekhar and co-workers reported a series of quinoxaline aryl ethers as potential anticancer agents targeting c-Met-associated signaling [136]. The molecular design combined a quinoxaline core with a piperazine-containing region and a substituted phenoxy group. The piperazine moiety served as a basic, potentially solubilizing element and provided a convenient site for late-stage diversification.

**Scheme 19:**
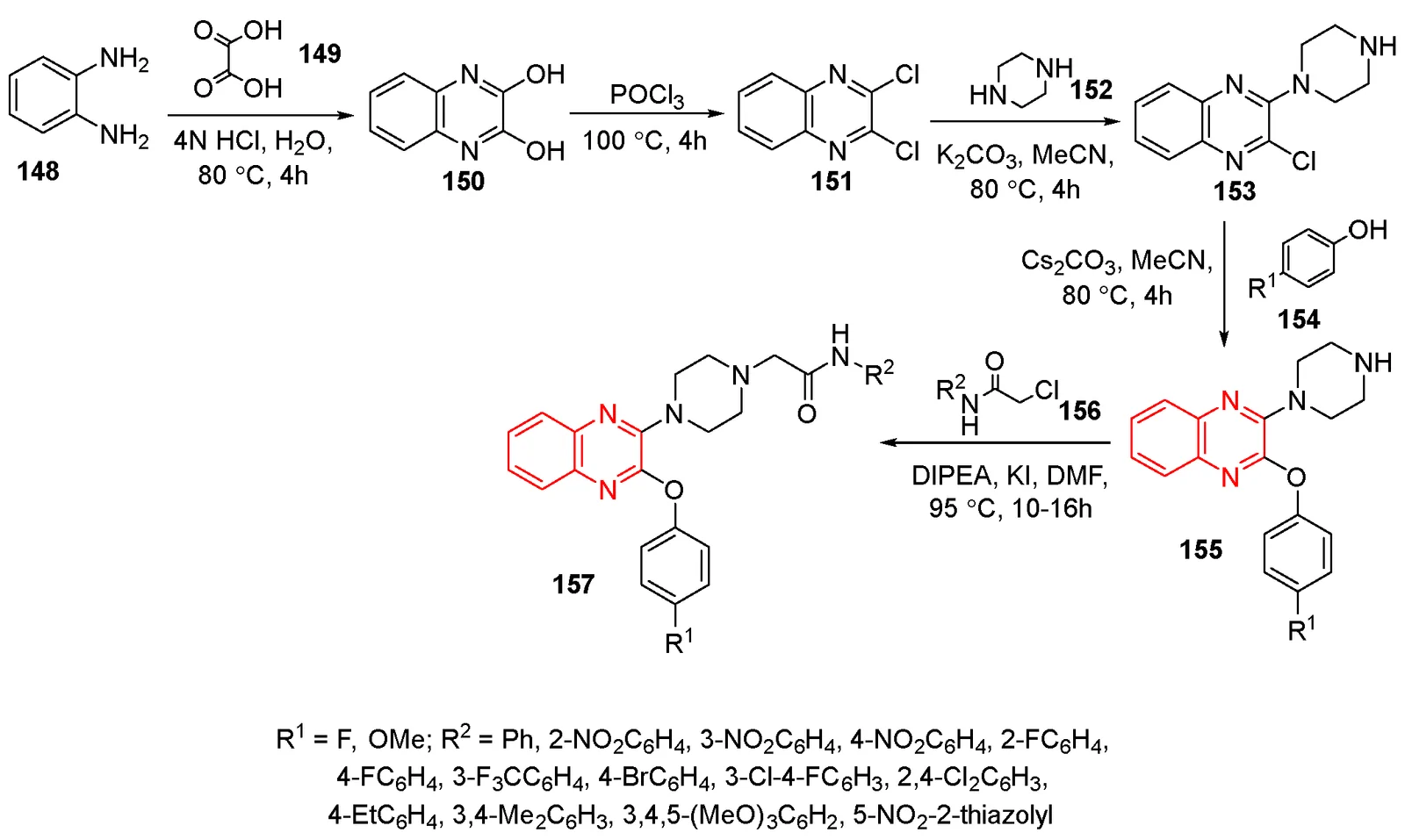
Synthetic route towards quinoxaline aryl ethers **157**.

The target compounds **157** were synthesized via nucleophilic aromatic substitution of 2-chloro-3-(piperazin-1-yl)quinoxaline **153** with substituted phenols to afford intermediates **155**, which were then coupled with substituted chloroanilides **156** to yield the final derivatives **157** (Scheme 19) [136].

Biological evaluation showed that the simpler intermediates **155** exhibited greater antiproliferative activity than the more structurally elaborate final derivatives **157**. In particular, the para-fluoro and para-methoxy analogs were the most active compounds in the series. The fluoro-substituted derivative inhibited DU145, HCT116, HEK-293, and MDA-MB-231 cells with IC_50_ values of 9.35, 13.54, 18.25, and 8.01 μM, respectively, whereas the methoxy analog produced IC_50_ values of 13.08, 15.46, 22.36, and 11.81 μM [136].

Among the tested models, the strongest activity was observed in MDA-MB-231 triple-negative breast cancer cells, where both compounds outperformed 5-fluorouracil under the reported conditions. The superior activity of the fluoro-substituted derivative compared with the corresponding methoxy analog suggests that fluorine provided a more favorable balance of electronic and physicochemical properties within this scaffold.

Mechanistic studies focused on the two active intermediates **155**. Treatment of MDA-MB-231 cells increased apoptotic cell populations, as demonstrated by Annexin V/7-aminoactinomycin D staining, and caspase-3/7 activation supported the induction of apoptosis [136]. Western blot analysis further indicated suppression of c-Met-associated signaling pathways following compound treatment.

Although these observations support involvement of c-Met signaling, direct biochemical inhibition of c-Met was not reported. Consequently, the active quinoxaline derivatives are more appropriately described as c-Met-pathway modulators rather than as confirmed direct c-Met kinase inhibitors. The observed effects may result from direct target engagement, altered receptor expression or stability, modulation of upstream signaling pathways, or secondary consequences of cellular stress and apoptosis.

Molecular docking studies using the c-Met kinase structure (PDB ID: 3F66) predicted favorable accommodation of the active intermediates within the ATP-binding site, and molecular dynamics simulations supported the stability of the proposed protein–ligand complexes [136]. These computational results provide a plausible structural rationale for the observed pathway modulation but do not independently establish direct kinase inhibition.

Overall, the quinoxaline series highlights a common observation in medicinal chemistry: increasing molecular complexity did not improve biological activity. The simpler fluoro- and methoxy-substituted intermediates outperformed their more elaborated analogs, suggesting that preserving a compact quinoxaline–phenoxy pharmacophore may be preferable to extensive structural expansion in this scaffold class. Further studies incorporating direct c-Met enzyme assays, cellular target-engagement experiments, and broader kinase profiling will be required to clarify the mechanism of action and assess the potential of quinoxaline-based compounds as c-Met-directed therapeutics.

### 4.10. Benzo[d]thiazole-Based c-Met Inhibitors

Benzo[*d*]thiazole is a privileged sulfur- and nitrogen-containing bicyclic heterocycle that has attracted considerable interest in anticancer drug discovery for its rigid, planar structure, favorable electronic properties, and ability to engage in hydrogen-bonding, hydrophobic, and π-mediated interactions. The benzothiazole ring system provides an extended aromatic surface suitable for kinase-binding pockets and tolerates extensive structural diversification through scaffold fusion and peripheral substitution. These features make benzo[*d*]thiazole an attractive platform for developing c-Met-directed anticancer agents.

A structurally diverse series of benzo[*d*]thiazole-containing fused heterocycles **159**–**165** was synthesized using a combination of multicomponent and cyclization reactions [137]. The synthetic program yielded several distinct scaffold classes, including chromenones **160**, quinolinones **161**, thieno[3,2-*f*]chromenes **162**, chromeno[5,6-*d*]thiazoles **163**, and pyrano[2,3-*g*]indazoles **165**, enabling evaluation of the effects of scaffold fusion and conformational restriction while preserving a common benzothiazole pharmacophore.

**Scheme 20:**
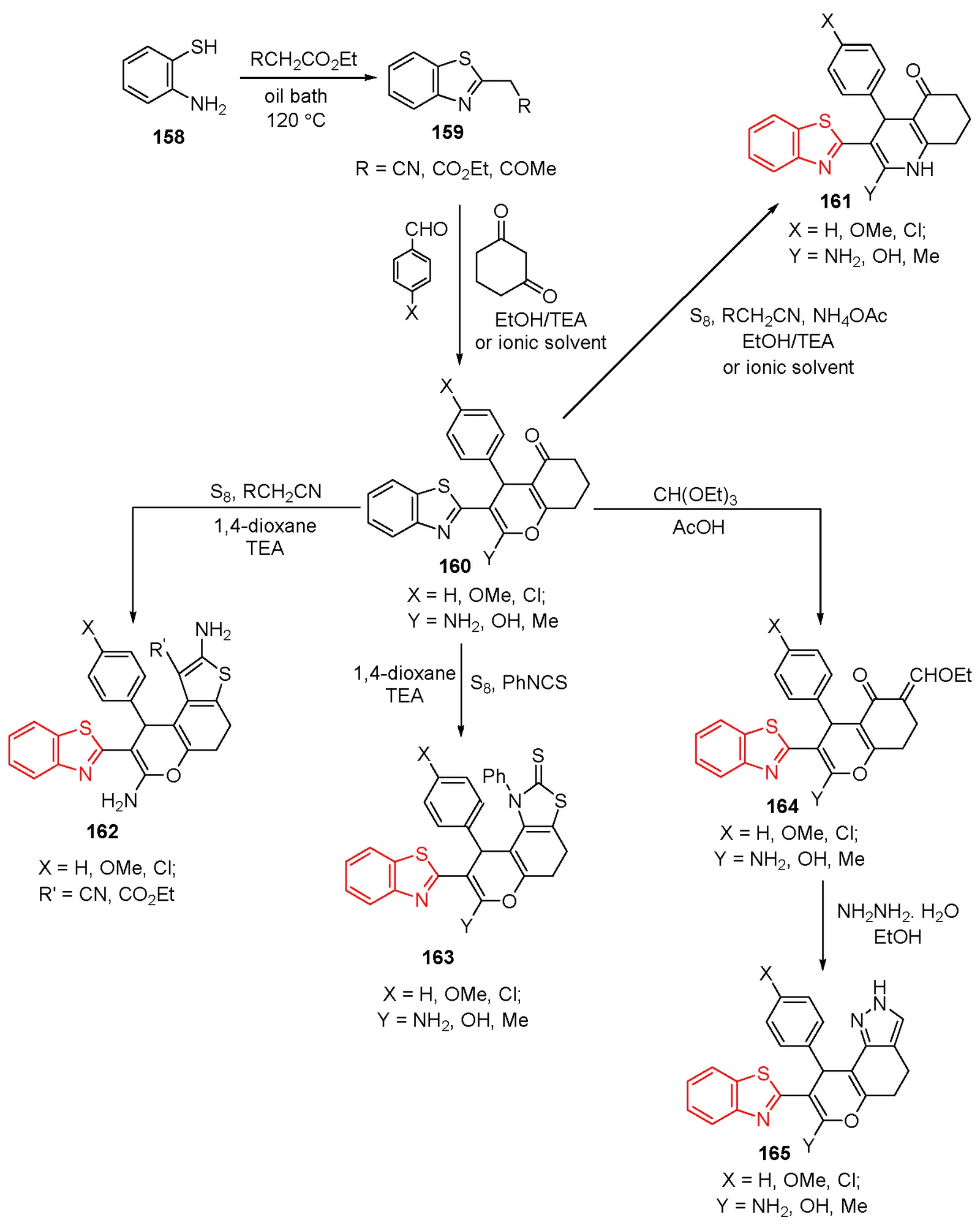
Synthetic pathway to benzo[*d*]thiazole-containing fused heterocycles **159**–**165**.

The benzo[*d*]thiazol-2-yl precursors **159** were prepared from 2-aminothiophenol **158** and multicomponent reactions and heterocyclization steps, then furnished the target fused-ring systems (Scheme 20) [137].

The compounds were evaluated against HepG2 hepatocellular carcinoma and HeLa cervical cancer cells using the MTT assay, with VERO cells included as a nonmalignant comparator. Several derivatives exhibited potent antiproliferative activity while showing substantially lower toxicity toward VERO cells.

Among the series, compounds **161**, **163**, and **164** were the most active derivatives. Compound **164** inhibited HepG2 and HeLa cells with IC_50_ values of 0.18 and 0.24 μM, respectively, and exhibited an IC_50_ of 44.63 μM against VERO cells [137]. Similarly, compounds **163** and **161** displayed IC_50_ values of 0.19/0.26 μM and 0.21/0.26 μM against HepG2 and HeLa cells, with VERO-cell IC_50_ values above 60 μM [137]. These results indicate a favorable preliminary separation between tumor-cell activity and nonmalignant-cell toxicity.

Direct biochemical evaluation revealed exceptionally potent inhibition of c-Met. Compounds **161**, **163**, and **164** inhibited c-Met with IC_50_ values of 0.15, 0.12, and 0.15 nM, respectively, compared with foretinib’s 1.16 nM under the same assay conditions [137]. Thus, the most active members of the series achieved subnanomolar enzyme potency and were substantially more potent than foretinib in the reported biochemical assay.

Despite their exceptional enzyme activity, a pronounced gap remained between biochemical and cellular potency. c-Met inhibition occurred at approximately 0.12–0.15 nM, whereas antiproliferative activity was observed in the 0.18–0.26 μM range. This difference suggests that factors such as cellular exposure, intracellular target engagement, ATP competition, protein binding, or tumor dependence on c-Met signaling influence the overall biological response.

The consistent activity of compounds **161**, **163**, and **164**, despite their belonging to different fused-ring subclasses, suggests that the shared benzothiazole-containing pharmacophore and the common chloro/hydroxyl substitution pattern contribute significantly to activity. The rigid fused-ring architectures may also favor productive target recognition by reducing conformational flexibility.

Taken together, direct subnanomolar c-Met inhibition and submicromolar cellular activity identify this series as one of the most potent scaffold classes discussed in this review. Although broader kinase-selectivity profiling and cellular target-engagement studies remain necessary, compounds **161**, **163**, and **164** represent experimentally validated c-Met inhibitors and demonstrate the considerable promise of benzothiazole-containing fused heterocyclic systems for future c-Met-directed drug discovery [137].

### 4.11. Thieno[2,3-b]pyridine-Based c-Met Inhibitors

Thieno[2,3*-b*]pyridine is a sulfur-containing fused heterocyclic scaffold that has emerged as an attractive bioisosteric alternative to quinoline in kinase inhibitor design. Replacing the benzene component of quinoline with a thiophene ring alters the electronic distribution and polarizability of the fused system while preserving the pyridine nitrogen required for hinge-region recognition. These features make thieno[2,3-*b*]pyridine a useful scaffold for exploring alternative ATP-competitive kinase inhibitors, including those targeting c-Met.

Using a scaffold-hopping strategy, Wang and co-workers designed a series of thieno[2,3-*b*]pyridine derivatives **169** based on the pharmacophoric architecture of cabozantinib **10** [138]. In this design, the quinoline hinge-binding region of cabozantinib was replaced with a thieno[2,3-*b*]pyridine nucleus, while the pyridine nitrogen was retained to preserve the proposed interaction with the hinge residue Met1160. In addition, the conventional flexible hydrogen-bonding motif present in many Type II c-Met inhibitors was replaced with a conformationally restricted 2-pyridone ring to maintain the required hydrogen-bonding geometry while reducing conformational flexibility.

**Scheme 21:**
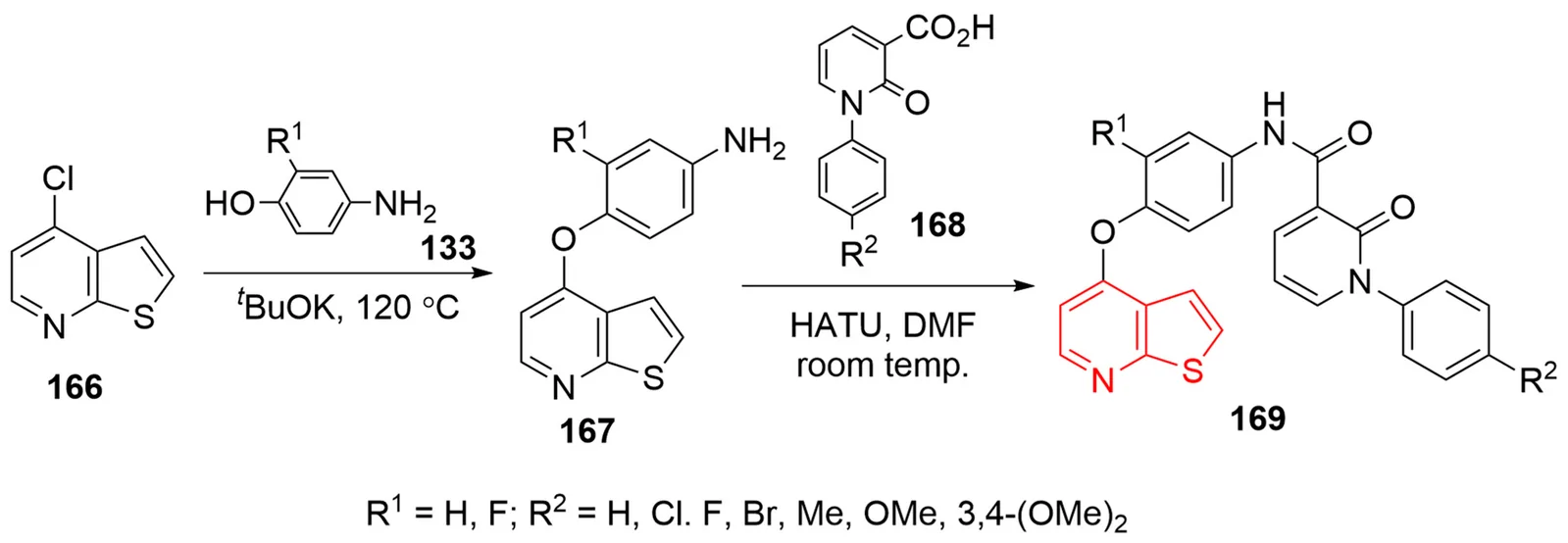
Synthetic route towards thieno[2,3-*b*]pyridines **169**.

The target compounds were synthesized via nucleophilic aromatic substitution of 4-chlorothieno[2,3-*b*]pyridines **166** with substituted 4-aminophenols **133** to afford intermediates **167**, followed by HATU-mediated coupling with substituted 1-aryl-2-oxo-1,2-dihydropyridine-3-carboxylic acids **168** to furnish the final derivatives **169** (Scheme 21) [138].

Biological evaluation against A549 non-small-cell lung, HeLa cervical, and MCF7 breast cancer cells identified the analog with R^1^ = F and R^2^ = H as the most active member of the series. The compound inhibited A549, HeLa, and MCF7 cells with IC_50_ values of 0.005, 2.833, and 13.581 μM, respectively [138]. Under the same experimental conditions, compared with cabozantinib, the compound showed markedly improved activity in A549 cells, similar activity in HeLa cells, and weaker activity in MCF7 cells. These results indicate a strongly cell-line-dependent response rather than uniformly superior potency.

The exceptionally low A549 IC_50_ of 5 nM is particularly noteworthy and was accompanied by inhibition of colony formation and suppression of cell migration in both A549 and HeLa models [138]. These findings suggest substantial effects on tumor-cell proliferation and motility. However, because direct c-Met inhibition was not measured, the observed cellular activity cannot be conclusively attributed to c-Met blockade.

Molecular docking with the c-Met kinase structure (PDB ID: 3LQ8) predicted binding orientations similar to those proposed for cabozantinib. The pyridine nitrogen was positioned to maintain the hinge-region interaction with Met1160, while the phenoxy linker and the 2-pyridone-containing region extended toward the hydrophobic and hydrogen-bonding regions associated with Type II inhibitor recognition [138]. These results support the structural feasibility of c-Met binding and provide a rationale for the observed biological activity.

Nevertheless, the study did not report direct biochemical c-Met inhibition, kinase selectivity profiling, or cellular target engagement experiments. Consequently, the available evidence supports classifying the series as putative c-Met-directed compounds rather than experimentally validated c-Met inhibitors. Additional biochemical and mechanistic studies will be required to determine whether the impressive cellular activity, particularly in A549 cells, arises primarily from c-Met inhibition, multitarget activity, or an alternative mechanism.

Overall, this work demonstrates that thieno[2,3-*b*]pyridine can serve as a viable quinoline replacement in c-Met inhibitor design and highlights the potential of scaffold hopping to generate structurally distinct compounds with promising anticancer activity. The series is particularly notable for achieving substantial cellular activity despite the absence of direct enzymatic validation, making it an interesting scaffold for future optimization and target-confirmation studies.

### 4.12. 1,2,4-Triazin-6-one-Based c-Met Inhibitors

The 1,2,4-triazin-6-one scaffold is a nitrogen-rich heterocycle that has attracted interest in medicinal chemistry for its synthetic accessibility, favorable hydrogen-bonding properties, and ability to mimic purine-like interactions. The heteroatom-rich core contains multiple hydrogen-bond donor and acceptor sites that may support recognition within kinase ATP-binding pockets. However, 1,2,4-triazin-6-one derivatives have been explored primarily as androgen receptor-targeted agents rather than as established kinase inhibitors. Consequently, their relevance to c-Met inhibitor discovery remains preliminary and requires direct experimental validation.

A series of 1,3-disubstituted-1,2,4-triazin-6-ones **177** during the development of new therapeutics for prostate cancer [139]. The compounds were designed as antiandrogen agents and were subsequently evaluated for potential c-Met-binding capability using molecular docking studies.

**Scheme 22:**
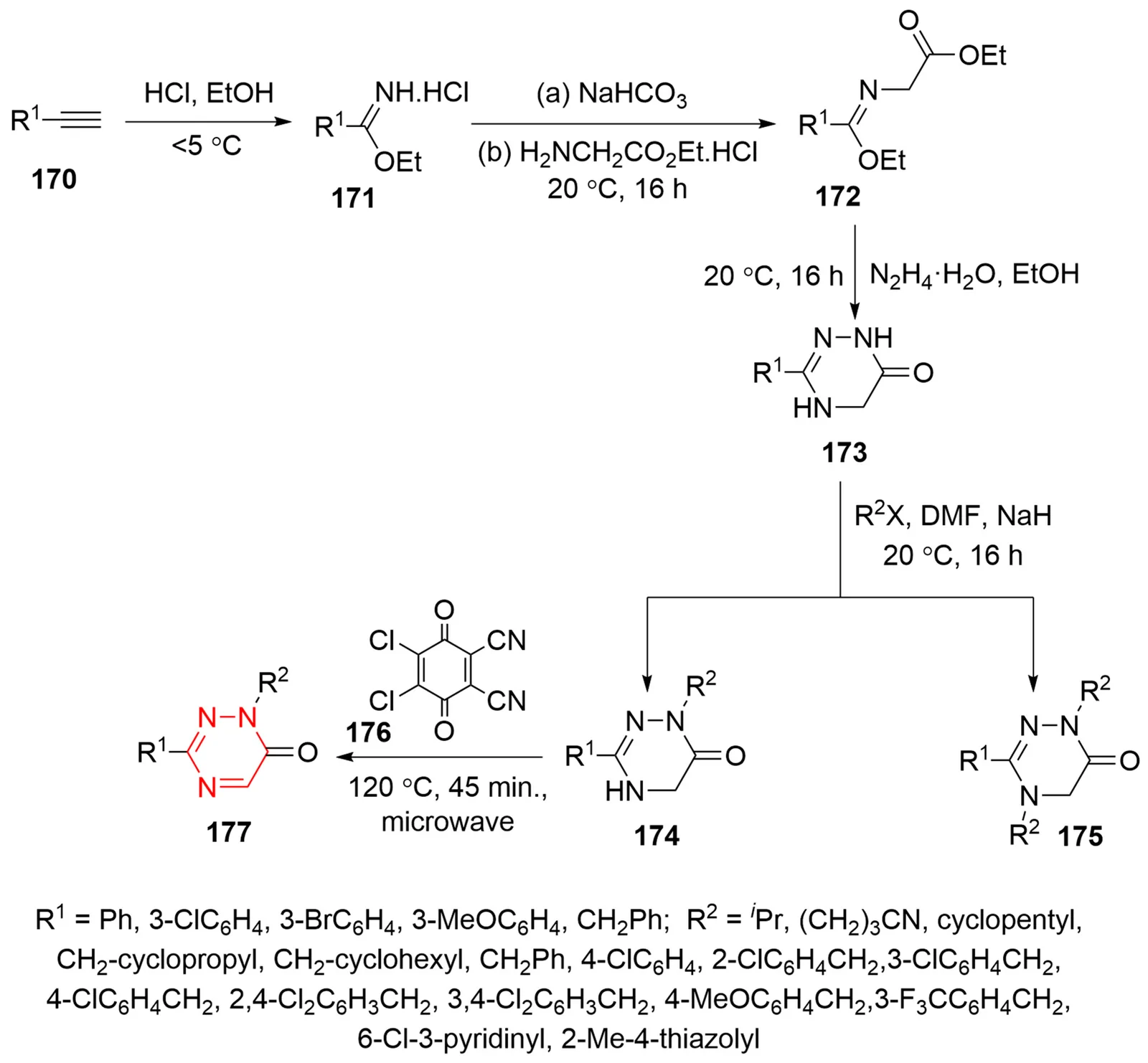
Synthetic pathway for 1,3-disubstituted-1,2,4-triazin-6-ones **177**.

The target compounds were synthesized from substituted nitriles **170** via formation of ethyl imidate hydrochlorides **171**, conversion to glycine imidates **172**, cyclization to 1,2,4-triazin-6(1*H*)-ones **173**, alkylation to afford intermediates **174**, and final oxidation with DDQ **176** under microwave conditions to furnish the desired 1,3-disubstituted-1,2,4-triazin-6-ones **177** (Scheme 22) [139]. The modular route enabled independent modification of substituents at the 1- and 3-positions of the triazinone core, facilitating exploration of hydrophobic and halogenated aromatic groups.

**Figure 12:**
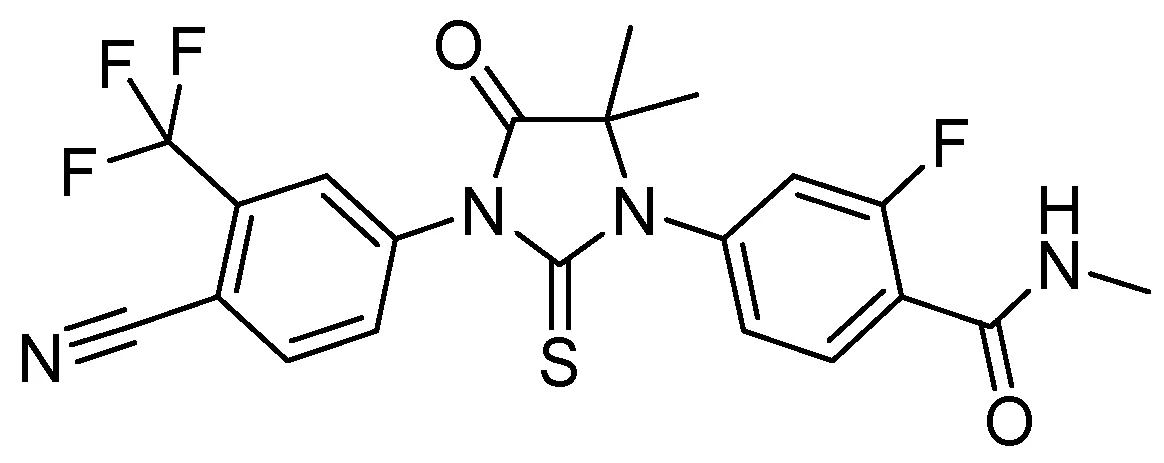
Chemical structure of enzalutamide **178**, an FDA-approved androgen receptor inhibitor used as the reference compound.

The synthesized compounds were evaluated against 22Rv1 androgen receptor-dependent and PC-3 androgen receptor-independent prostate cancer cells. Among the series, the derivative bearing a benzyl group and a 2,4-dichlorobenzyl substituent emerged as the lead compound. It exhibited IC_50_ values of 0.18 μM against 22Rv1 cells and 1.48 μM against PC-3 cells, compared with 36.0 μM and 42.0 μM for enzalutamide **178** (Figure 12), respectively [139].

The strong activity observed in both androgen receptor-dependent and androgen receptor-independent models suggests that the compound may act through mechanisms beyond classical androgen receptor antagonism. Consistent with this observation, the lead derivative demonstrated favorable reported pharmacokinetic properties and significant antitumor activity in a 22Rv1 xenograft model, supporting adequate in vivo exposure and biological activity [139].

To explore alternative targets, molecular docking studies were conducted using the c-Met kinase structure (PDB ID: 3CD8). The simulations suggested that the triazinone derivative could adopt a favorable orientation within the ATP-binding region, with the heteroatom-rich core contributing polar interactions and the benzyl substituents occupying hydrophobic regions of the binding pocket [139].

Although these results provide a plausible rationale for c-Met engagement, no direct biochemical c-Met inhibition, biophysical binding measurements, cellular target engagement studies, or c-Met phosphorylation analyses were reported. Consequently, the available evidence does not support classifying this series as confirmed c-Met inhibitors. Instead, the lead compound should be regarded as a triazinone anticancer agent with predicted c-Met-binding potential.

The study nevertheless demonstrates the utility of molecular docking for identifying potential secondary targets and suggests that nitrogen-rich triazinone scaffolds may be adapted for kinase-directed drug discovery. Future studies should include direct c-Met kinase assays, kinase-selectivity profiling, cellular target-engagement experiments, and pathway-phosphorylation analyses to determine whether c-Met modulation meaningfully contributes to the observed antitumor activity.

### 4.13. Pyrazolo[3,4-b]pyridine-Based c-Met Inhibitors

Pyrazolo[3,4-*b*]pyridine is a fused bicyclic heterocycle that has attracted considerable interest in kinase inhibitor discovery for its rigid, purine-mimetic architecture and its suitability for ATP-pocket recognition. The adjacent pyrazole and pyridine nitrogen atoms provide multiple potential hydrogen-bond donor and acceptor sites, while the fused aromatic framework can support hydrophobic and π-mediated interactions within kinase-binding pockets. Its structural rigidity may reduce the conformational penalty associated with target binding, making pyrazolo[3,4-*b*]pyridine an attractive scaffold for developing ATP-competitive c-Met inhibitors.

**Scheme 23:**
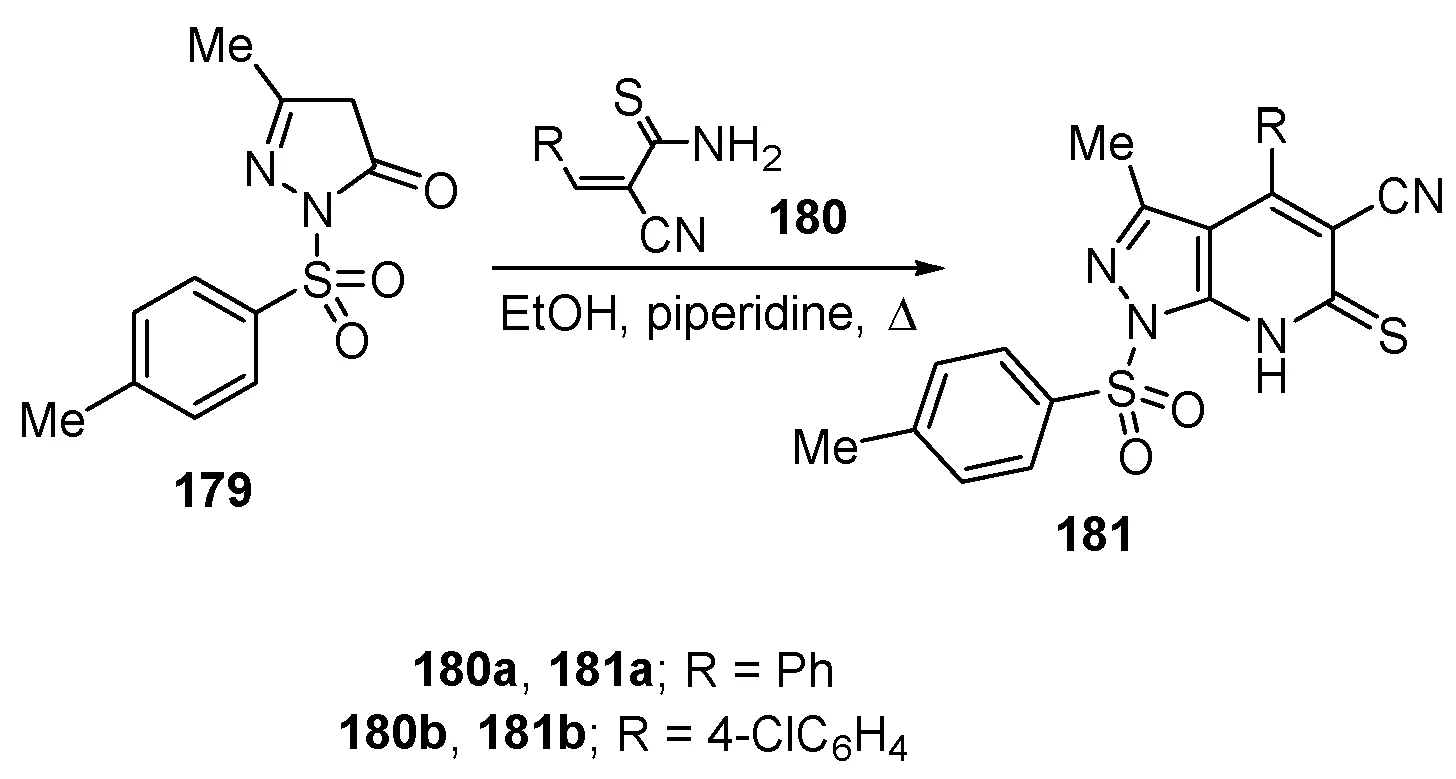
Synthetic route toward pyrazolo[3,4-*b*]pyridines **181**.

A set of pyrazolo[3,4-*b*]pyridine derivatives **181** was synthesized via a base-catalyzed cyclocondensation of pyrazol-5(4*H*)-one **179** with substituted arylidene cyanothioacetamides **180** in refluxing ethanol, using piperidine as the catalyst (Scheme 23) [140]. This concise synthetic route provided direct access to the fused pyrazolopyridine framework and allowed for variation in the aryl substituent.

The synthesized compounds were evaluated against HepG2 hepatocellular carcinoma, MCF7 breast cancer, and HCT116 colorectal cancer cells. Compounds **181a** and **181b** were the most active derivatives. Compound **181a** inhibited HepG2, MCF7, and HCT116 cells with IC_50_ values of 3.42, 5.25, and 6.27 μM, respectively, while compound **181b** produced corresponding IC_50_ values of 3.56, 6.82, and 9.21 μM [140]. Both compounds displayed low-micromolar antiproliferative activity comparable to the reference drugs 5-fluorouracil and erlotinib, although neither consistently outperformed the comparators across all cell lines.

Importantly, the compounds exhibited substantially lower cytotoxicity against WI-38 human lung fibroblasts, with IC_50_ values of 80.46 μM for **181a** and 85.10 μM for **181b** [140]. This yielded favorable preliminary selectivity windows, particularly for compound **181a** which showed the best overall balance of potency and selectivity.

Direct biochemical evaluation using a homogeneous time-resolved fluorescence assay confirmed potent c-Met inhibition. Compounds **181a** and **181b** inhibited c-Met with IC_50_ values of 4.27 and 7.95 nM, respectively, compared with cabozantinib at 5.38 nM under the same assay conditions [140]. These results demonstrate that the pyrazolo[3,4-*b*]pyridine scaffold can support low-nanomolar c-Met inhibition and establish **181a** as a particularly potent member of the series.

Notably, biochemical potency translated only partially into cellular activity. Although c-Met inhibition occurred at low-nanomolar concentrations, antiproliferative effects were observed at micromolar concentrations. This enzyme-to-cell potency shift is consistent with observations for several other scaffold classes discussed in this review and highlights the influence of intracellular exposure, ATP competition, target dependence, and other pharmacological factors beyond enzyme affinity alone.

Molecular docking studies using the c-Met crystal structure (PDB ID: 3U6I) predicted favorable accommodation of compounds **181a** and **181b** within the ATP-binding pocket and identified interactions between the fused nitrogen-containing core and key kinase-recognition residues [140]. Because direct biochemical inhibition was demonstrated, the computational studies primarily provide structural support for the experimentally observed activity.

Overall, the pyrazolo[3,4-*b*]pyridine scaffold is a compact purine-mimetic platform that supports potent c-Met inhibition. The combination of low-nanomolar biochemical activity, reasonable cellular potency, and favorable preliminary selectivity identifies compounds **181a** and **181b** as promising leads for further optimization. Future studies should focus on improving cellular translation, defining kinase selectivity, and establishing the structural basis for c-Met recognition through more detailed mechanistic and structural investigations.

### 4.14. Pyrrolo[2,3-d]pyrimidine-Based c-Met Inhibitors

Pyrrolo[2,3-*d*]pyrimidine is a privileged fused bicyclic heterocycle widely used in kinase inhibitor design because its purine-mimetic architecture closely resembles the adenine moiety of ATP. The scaffold contains multiple nitrogen atoms that can form productive interactions with kinase hinge-region residues while maintaining a favorable balance of rigidity and synthetic versatility. Consequently, pyrrolo[2,3-*d*]pyrimidines have emerged as attractive hinge-binding motifs for receptor tyrosine kinases, including c-Met.

**Figure 13:**
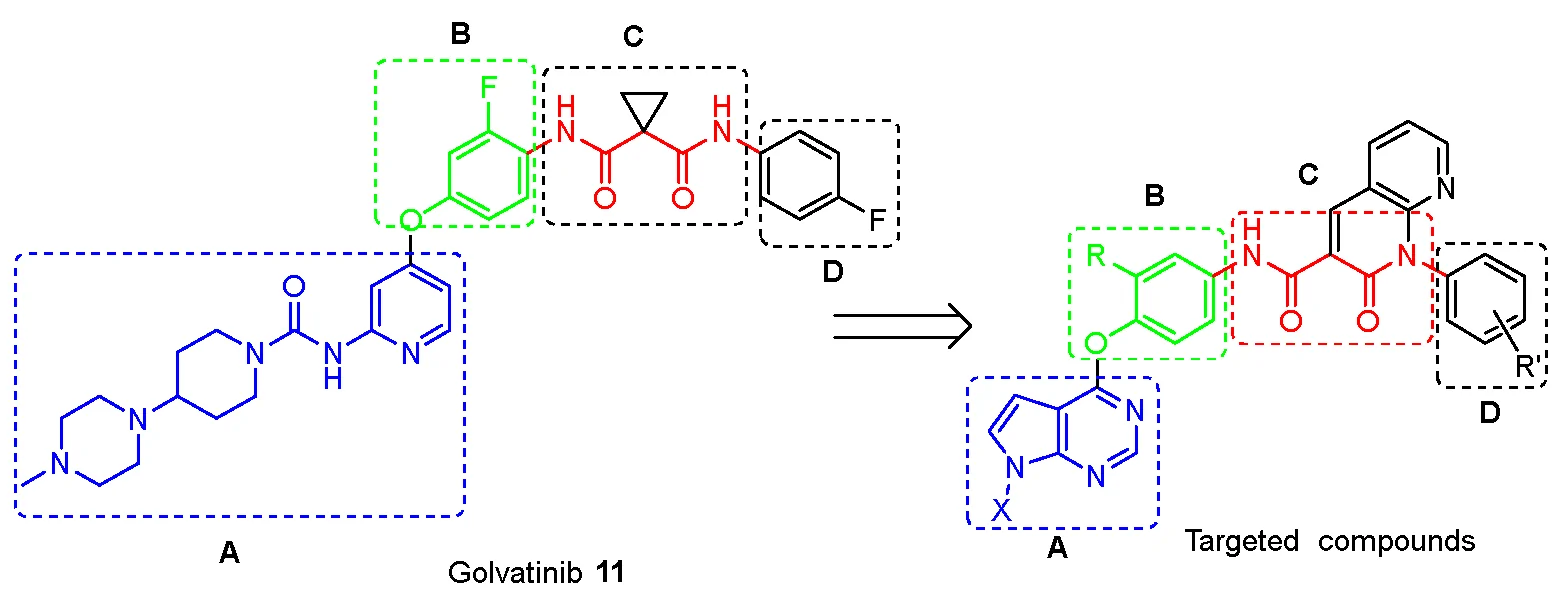
Design of the targeted pyrrolo[2,3-*d*]pyrimidine-containing compounds.

Guided by pharmacophore analysis of the Type II c-Met inhibitor golvatinib, Liu and colleagues designed a first-generation series of pyrrolo[2,3-*d*]pyrimidines **192** through a scaffold-hopping strategy [141]. The quinoline hinge-binding region of golvatinib was replaced with a pyrrolo[2,3-*d*]pyrimidine core, and a 1,8-naphthyridin-2-one moiety was introduced as a rigid hydrogen-bonding pharmacophore to preserve the extended architecture characteristic of Type II c-Met inhibitors (Figure 13).

**Scheme 24:**
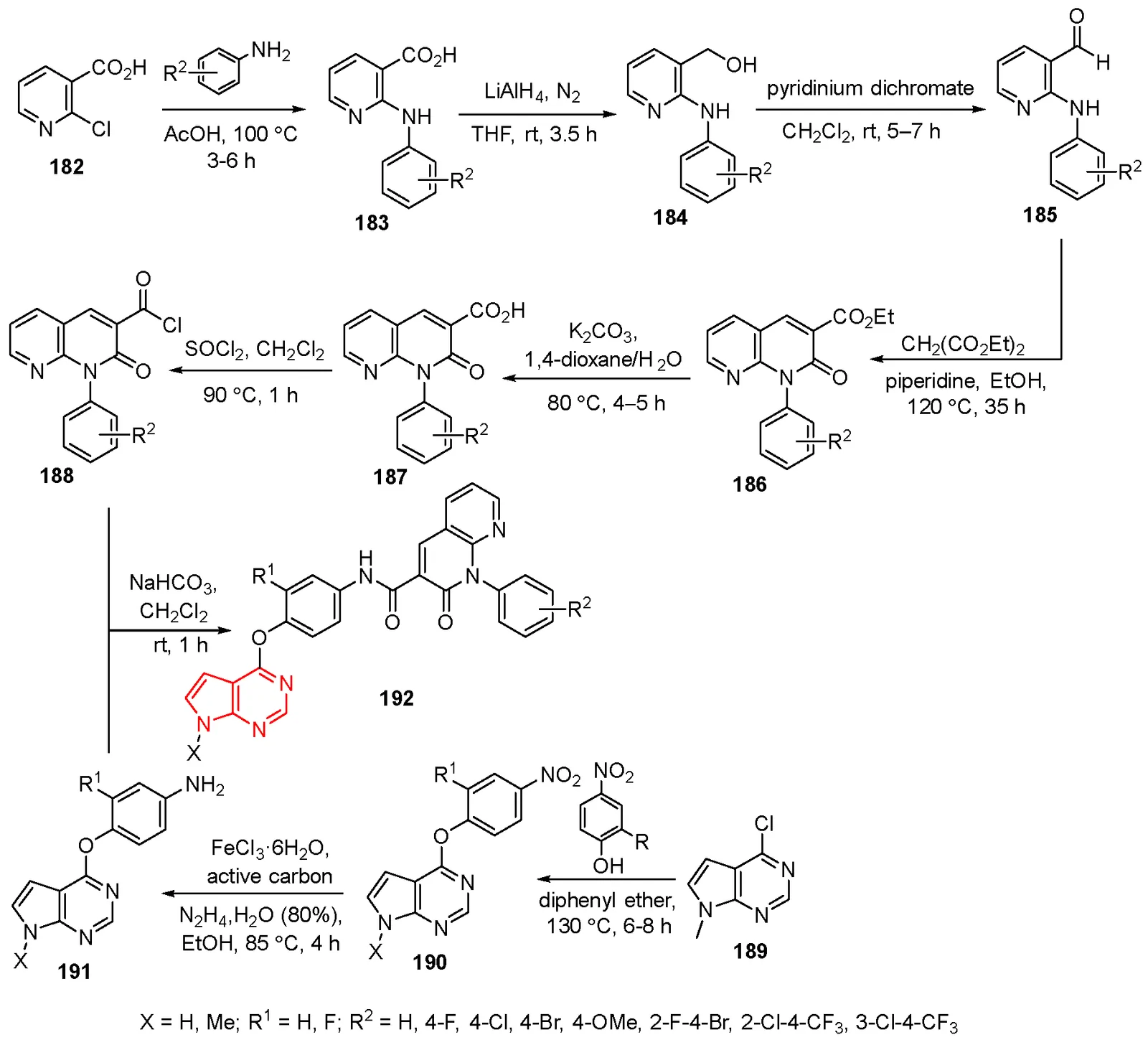
Synthetic route toward pyrrolo[2,3-*d*]pyrimidines **192** incorporating a 1,8-naphthyridin-2-one pharmacophore.

The target compounds **192** were synthesized via a convergent route that involved preparing 1,8-naphthyridinone-derived acid chlorides **188** and pyrrolo[2,3-*d*]pyrimidine-containing anilines **191**, followed by coupling to afford the desired products (Scheme 24) [141].

Biological evaluation against A549, HeLa, and MCF7 cells identified the derivative with X = H, R^1^ = F, and R^2^ = 4-F as the most active member of the series. The compound produced IC_50_ values of 5.29, 3.72, and 9.23 μM, respectively, representing modest improvements over golvatinib in the same cellular models [141]. Flow-cytometric analysis further showed induction of G_0_/G_1_-phase arrest in A549 cells.

Docking studies using the c-Met crystal structure (PDB ID: 3LQ8) suggested that the pyrrolo[2,3-*d*]pyrimidine nucleus could serve as a hinge-binding element, while the phenoxy linker and naphthyridinone region extended toward the hydrophobic back pocket associated with Type II inhibitor binding [141]. However, direct biochemical inhibition of c-Met was not reported for this first-generation series. Consequently, compounds **192** are best regarded as putative c-Met-directed derivatives, supported by pharmacophore design, cellular activity, and docking studies, rather than as experimentally validated c-Met inhibitors.

Building on this initial scaffold, the same group subsequently developed a second-generation series of pyrrolo[2,3-*d*]pyrimidines **199** that incorporate a 1,8-naphthyridin-4-one pharmacophore [142]. This modification was designed to optimize the orientation and hydrogen-bonding properties of the terminal heterocyclic region while preserving the pyrrolo[2,3-*d*]pyrimidine hinge-binding core and the overall Type II inhibitor architecture.

**Scheme 25:**
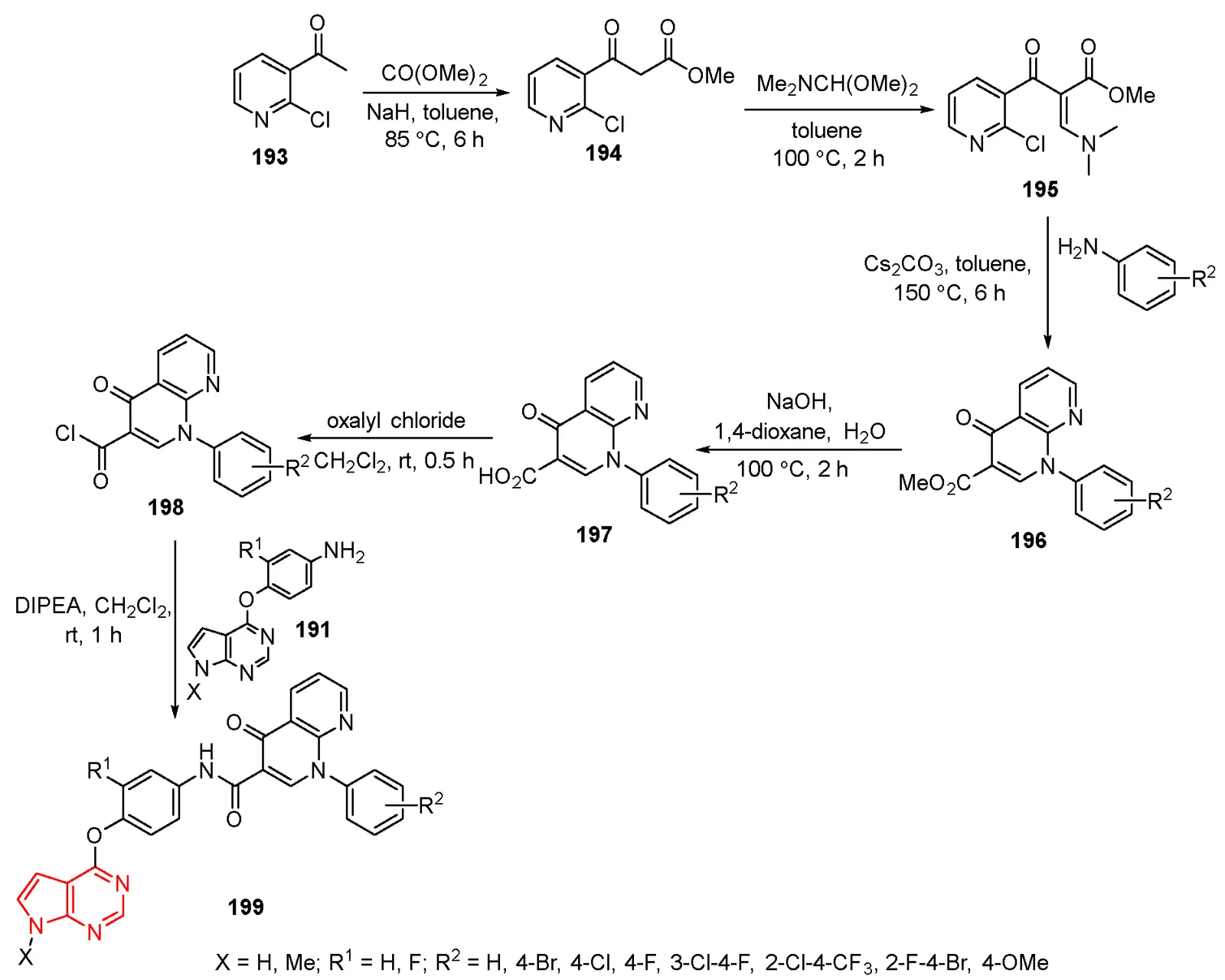
Synthetic route toward pyrrolo[2,3-*d*]pyrimidines **199** incorporating a 1,8-naphthyridin-4-one pharmacophore.

The target compounds **199** were synthesized by preparing functionalized naphthyridinone acid chlorides **198**, followed by coupling with pyrrolo[2,3-*d*]pyrimidine-containing anilines **191** (Scheme 25) [142].

The optimized lead compound, bearing R^2^ = 2-Cl-4-CF_3_, showed markedly improved cellular activity compared with the first-generation series. It inhibited A549, HeLa, and MCF7 cells with IC_50_ values of 0.66, 0.38, and 0.44 μM, respectively [142]. Under the same experimental conditions, cabozantinib produced IC_50_ values of 0.76, 0.32, and 0.45 μM, indicating that the lead compound’s cellular activity was broadly comparable to that of the approved drug.

Importantly, the second-generation lead showed substantially improved activity compared with the earlier scaffold, with approximately eightfold, tenfold, and twentyfold enhancements in A549, HeLa, and MCF7 cells, respectively. These results suggest that optimizing the naphthyridinone pharmacophore and the terminal aromatic substituent significantly improved cellular performance.

The compound also showed a favorable preliminary selectivity profile. An IC_50_ of 67.43 μM was reported against LO2 nonmalignant hepatic cells, yielding selectivity indices exceeding 100 relative to the tested cancer cell lines [142]. In addition, apoptosis was confirmed by acridine orange staining and Annexin V/propidium iodide analysis.

A notable feature of this series was its reported kinase selectivity profile. Unlike broad-spectrum inhibitors such as cabozantinib, the lead compound preferentially inhibited c-Met over PDGFR-β, FLT3, RON, VEGFR-2, c-Kit, and ALK [142]. Although quantitative selectivity values were not fully reported, the results suggest that scaffold optimization improved target specificity while maintaining strong cellular activity.

Docking studies using the c-Met crystal structure (PDB ID: 3LQ8) predicted productive interactions between the pyrrolo[2,3-*d*]pyrimidine hinge-binding region and key residues, including Met1160, Asp1222, and Phe1223 [142]. While these results support the proposed binding model, additional structural or kinetic studies would be needed to confirm whether the compounds function as Type II c-Met inhibitors.

Overall, the pyrrolo[2,3-*d*]pyrimidine scaffold is among the more promising chemotypes discussed in this review. The progression from first-generation compounds **192** to the optimized series **199** illustrates how scaffold refinement and pharmacophore optimization can dramatically improve cellular potency while maintaining favorable selectivity. Among the synthetic scaffolds reviewed, the second-generation pyrrolo[2,3-*d*]pyrimidines offer one of the most balanced combinations of cellular activity, preliminary selectivity, and mechanistic support for c-Met-directed anticancer drug discovery.

### 4.15. Triazolotriazine-Based c-Met Inhibitors

Triazolotriazines are compact, nitrogen-rich fused heterocycles with structural and electronic features well-suited for kinase inhibitor design. Their high heteroatom density enables productive interactions with kinase hinge regions, while their rigid, planar framework may minimize the conformational penalty of ATP-pocket binding. Although less extensively explored than pyridine-, quinoline-, or pyrimidine-based scaffolds, triazolotriazines have emerged as promising platforms for potent c-Met inhibition.

**Scheme 26:**
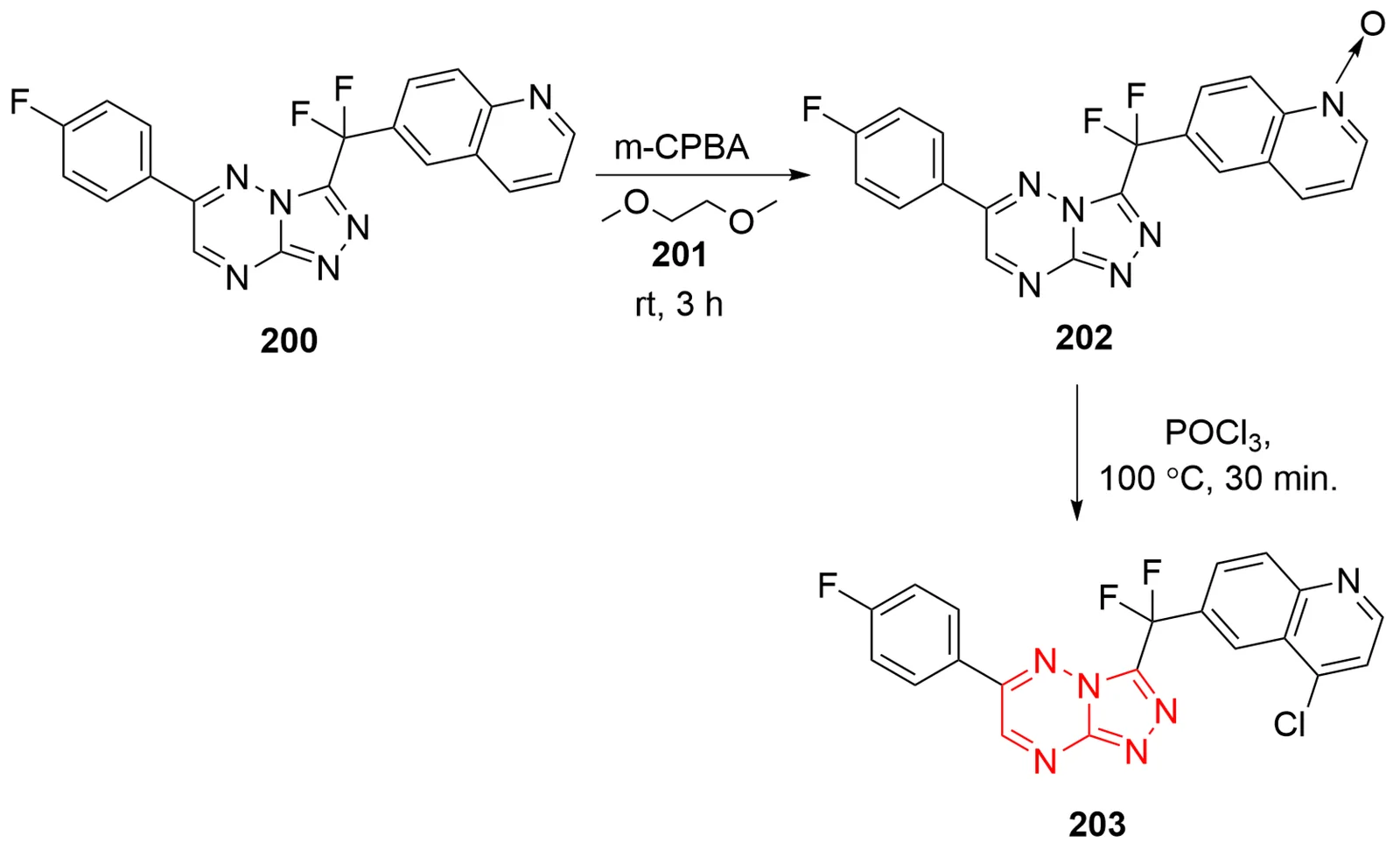
Synthetic route toward [1,2,4]triazolo [4,3-*b*][1,2,4]triazine **203**.

4-Chloro-[1,2,4]triazolo[4,3-*b*][1,2,4]triazine **203** was reported as a potent c-Met inhibitor [22,33]. The target compound was prepared by oxidizing the parent triazolotriazine **200** to the corresponding N-oxide **202**, followed by chlorination with POCl_3_ to afford the 4-chloro derivative **203** (Scheme 26). This concise synthetic route highlights the attractiveness of this compact scaffold for medicinal chemistry optimization.

Biological evaluation in c-Met-dependent EBC-1 non-small-cell lung cancer and MKN-45 gastric cancer cells revealed pronounced antiproliferative activity. Compound **203** produced IC_50_ values of <0.2 nM and 0.3 nM, respectively, substantially outperforming the clinical-stage c-Met inhibitor JNJ-38877605 **2** (Figure 1) under the same experimental conditions [22,33].

Direct biochemical evaluation confirmed potent c-Met inhibition, with compound **203** exhibiting an IC_50_ of 1.8 nM, compared with 0.95 nM for JNJ-38877605 [22,33]. Although slightly less potent than the comparator in the isolated enzyme assay, compound **203** showed markedly stronger activity in c-Met-driven cellular models, indicating exceptionally efficient translation of biochemical activity into cellular efficacy.

This scaffold shows a notable relationship between biochemical and cellular potency. Whereas many kinase inhibitor series show substantial loss of activity when moving from enzyme assays to cellular systems, compound **203** maintained its biochemical potency and, in cellular terms, appeared to exceed it. This observation suggests particularly effective engagement of c-Met-dependent signaling pathways in the tested tumor models, although the molecular basis remains unclear.

The available data establish compound **203** as an experimentally validated c-Met inhibitor and highlight the triazolotriazine nucleus as a highly efficient hinge-binding pharmacophore. However, broad kinase-selectivity profiling and detailed mechanistic studies have not yet been reported. Consequently, the scaffold’s broader selectivity profile and resistance coverage remain to be established.

Overall, the triazolotriazine series shows that highly compact, nitrogen-rich heterocycles can achieve potent c-Met inhibition while maintaining exceptional cellular activity. Among the scaffold classes discussed in this review, compound **203** is one of the most striking examples of efficient translation from biochemical inhibition to c-Met-dependent cellular activity, underscoring the potential of triazolotriazines as next-generation kinase-inhibitor scaffolds.

### 4.16. Comparative SAR Analysis of Synthetic c-Met Inhibitors

The structurally diverse synthetic compounds discussed in this review highlight several recurring medicinal chemistry principles relevant to the design of c-Met inhibitors. Potent ATP-competitive inhibitors generally contain (i) a nitrogen-containing heterocycle capable of recognizing the kinase hinge region, (ii) strategically positioned hydrogen-bond donors and acceptors, and (iii) hydrophobic substituents that occupy the ATP-binding pocket and adjacent regulatory regions. However, the contribution of these features depends heavily on scaffold geometry, substitution pattern, binding mode, and physicochemical properties, indicating that no single pharmacophore arrangement applies uniformly to all Type I and Type II c-Met inhibitors.

A consistent feature among validated c-Met inhibitors is the presence of hinge-binding heterocycles such as pyridines, pyrimidines, quinolines, thiazoles, pyrazolo[3,4-*b*]pyridines, pyrrolo[2,3-*d*]pyrimidines, and triazolotriazines. These scaffolds frequently position heteroatoms to interact with the hinge residue Met1160 and facilitate ATP-pocket recognition. In many Type II inhibitor series, a hinge-binding heterocycle is linked through a phenoxy spacer to a hydrogen-bond donor/acceptor motif and a terminal hydrophobic group, enabling simultaneous occupation of the ATP-binding site and the adjacent hydrophobic back pocket. Interactions involving Met1160, Lys1110, Asp1222, and Phe1223 repeatedly emerged as important contributors to inhibitor potency. Scaffold-hopping approaches further demonstrated that quinoline-derived hinge-binding motifs could be successfully replaced by pyridine-, pyrimidine-, thieno[2,3-*b*]pyridine-, or pyrrolo[2,3-*d*]pyrimidine-based systems while maintaining c-Met inhibitory activity, highlighting the flexibility of the hinge-recognition pharmacophore.

Several structural modifications were recurrently associated with improved biological activity. Fluorine, chlorine, and trifluoromethyl substituents frequently enhanced biochemical potency and cellular activity, possibly through favorable hydrophobic interactions, conformational effects, increased membrane permeability, and improved metabolic stability. Hydrophobic terminal aromatic groups commonly improved occupancy of lipophilic regions within the kinase binding site, whereas piperazine-, morpholine-, and related cyclic amine substituents often improved aqueous compatibility and cellular exposure. Likewise, 1,8-naphthyridinone-containing pharmacophores and extended 4-phenoxy linker architectures repeatedly produced favorable activity profiles by enabling optimal orientation of hinge-binding and terminal recognition motifs. Conformational restriction through fused-ring systems such as benzothiazoles, pyrazolopyridines, and triazolotriazines often reduced entropic penalties associated with target binding and contributed to enhanced inhibitory activity.

Another recurring medicinal chemistry theme was the successful application of scaffold hopping, bioisosteric replacement, and molecular hybridization strategies. Replacement of quinoline nuclei with pyridine, pyrimidine, thieno[2,3-*b*]pyridine, or pyrrolo[2,3-*d*]pyrimidine scaffolds expanded chemical diversity while preserving critical c-Met recognition elements. Similarly, molecular hybridization approaches generated compounds capable of simultaneously modulating c-Met and complementary therapeutic targets, producing multitarget inhibitors directed against VEGFR-2, EGFR, AXL, MER, PARP1, Pim-1, and other oncogenic pathways. Such polypharmacological designs may provide advantages by addressing pathway redundancy and adaptive resistance mechanisms; however, broad kinase activity does not inherently translate into superior therapeutic efficacy. Effective multitarget inhibitors require biologically relevant target combinations, balanced potency profiles, and acceptable safety margins.

One important observation across multiple scaffold classes was that potent biochemical inhibition of c-Met did not consistently translate into strong cellular antiproliferative activity. For example, some compounds, such as 1,3-thiazole derivatives **131**, benzothiazole compounds **161**, **163**, and **164**, and pyrazolo[3,4-b]pyridines **181a,b**, showed very strong inhibition of purified c-Met but required much higher concentrations to inhibit cancer cell growth. This gap is likely due to factors such as membrane permeability, ATP competition within cells, protein binding, metabolic stability, efflux, and the degree to which each tumor model depends on c-Met signaling. So, just improving enzyme potency is not enough for lead optimization. Future work should combine biochemical activity with tests for cellular target engagement, physicochemical properties, pharmacokinetics, and pharmacodynamics. On the other hand, triazolotriazine **202** showed very strong activity in c-Met-dependent EBC-1 and MKN-45 models, highlighting the importance of cellular context when assessing inhibitor performance.

The reviewed compounds also differed substantially in the strength of evidence supporting their classification as c-Met inhibitors. Several scaffold classes, including pyridine, quinoline, thiazole, pyrimidine, benzothiazole, pyrazolo[3,4-*b*]pyridine, pyrrolo[2,3-*d*]pyrimidine, and triazolotriazine derivatives, were supported by direct biochemical inhibition data and therefore constitute experimentally validated c-Met inhibitors. In contrast, some compounds acted primarily as c-Met-pathway modulators by suppressing receptor phosphorylation or downstream signaling without direct confirmation of kinase inhibition, as exemplified by the active quinoxaline derivatives **155**. Other compounds, including thieno[2,3-b]pyridines **169**, triazinones **177**, and selected indolinone-, pyrazole-, and quinazolinone-containing derivatives, were supported principally by docking studies or indirect biological observations and should therefore be regarded as putative c-Met-directed compounds pending further validation. This distinction matters because docking provides valuable hypotheses about target engagement but should not be interpreted as definitive evidence of direct kinase inhibition.

In summary, the trends in Table 3 are recurring observations, not strict SAR rules. Many studies examined small sets of analogs, altered several structural features simultaneously, or employed different biological assays. Because of this, compare studies carefully. Still, the overall evidence offers useful guidance for designing selective and multitarget c-Met inhibitors. Key points include hinge recognition, filling hydrophobic pockets, optimizing scaffolds, balancing physicochemical properties, ensuring cellular target engagement, and thorough experimental validation.

## 5. Multi-Target Drug Design Strategies for c-Met Inhibitors

Although selective c-Met inhibitors have achieved significant clinical success, acquired resistance, activation of compensatory signaling pathways, and the molecular heterogeneity of advanced cancers often limit their long-term efficacy. Tumor cells can escape c-Met inhibition by upregulating alternative receptor tyrosine kinases, including EGFR, VEGFR, AXL, MER, and RON, or by reactivating downstream signaling networks such as PI3K/AKT, MAPK, and STAT3. Consequently, inhibition of c-Met alone is often insufficient to achieve durable suppression of oncogenic signaling.

Combination therapy with multiple targeted agents is one approach to overcoming these limitations but is often associated with increased treatment complexity, cumulative toxicities, pharmacokinetic variability, and potential drug–drug interactions. As an alternative, medicinal chemists have increasingly adopted multi-target-directed ligand (MTDL) strategies, in which a single molecule is designed to modulate multiple therapeutically relevant targets simultaneously.

Application of this strategy to c-Met inhibitor discovery has led to the development of numerous dual- and multitarget agents that suppress complementary pathways involved in tumor growth, angiogenesis, invasion, metastasis, DNA repair, and therapeutic resistance. By concurrently targeting c-Met and additional oncogenic mediators such as VEGFR-2, EGFR, AXL, MER, PARP1, CDKs, and tubulin, these compounds may reduce pathway redundancy, delay resistance, broaden antitumor activity, and simplify treatment compared with combination regimens [143,144,145].

From a medicinal chemistry perspective, successful multitarget inhibitors typically arise from molecular hybridization, pharmacophore fusion, linker-based integration, or scaffold-hopping approaches that integrate the essential recognition elements of multiple targets into a single chemical framework. The clinical success of cabozantinib and the growing number of investigational multitarget agents underscore the therapeutic potential of this strategy.

The following sections summarize recent advances in dual- and multitarget c-Met inhibitor design, emphasizing the medicinal chemistry principles used to balance target potency, selectivity, and drug-like properties. Particular attention is given to compounds that simultaneously target c-Met and VEGFR-2, EGFR, CDK2, PARP1, MER, AXL, and tubulin, as well as related cancer-relevant pathways, which have emerged as promising approaches for overcoming resistance and improving anticancer efficacy.

### 5.1. c-Met/VEGFR-2 Dual Inhibitors

Simultaneously inhibiting c-Met and vascular endothelial growth factor receptor-2 (VEGFR-2) is among the most successful multitarget strategies in anticancer drug discovery. c-Met signaling primarily regulates tumor-cell proliferation, invasion, epithelial–mesenchymal transition (EMT), and metastasis, whereas VEGFR-2 is a central mediator of tumor angiogenesis [146,147]. Crosstalk between these pathways frequently drives tumor progression and therapeutic resistance, providing a strong biological rationale for dual inhibition.

**Figure 14:**
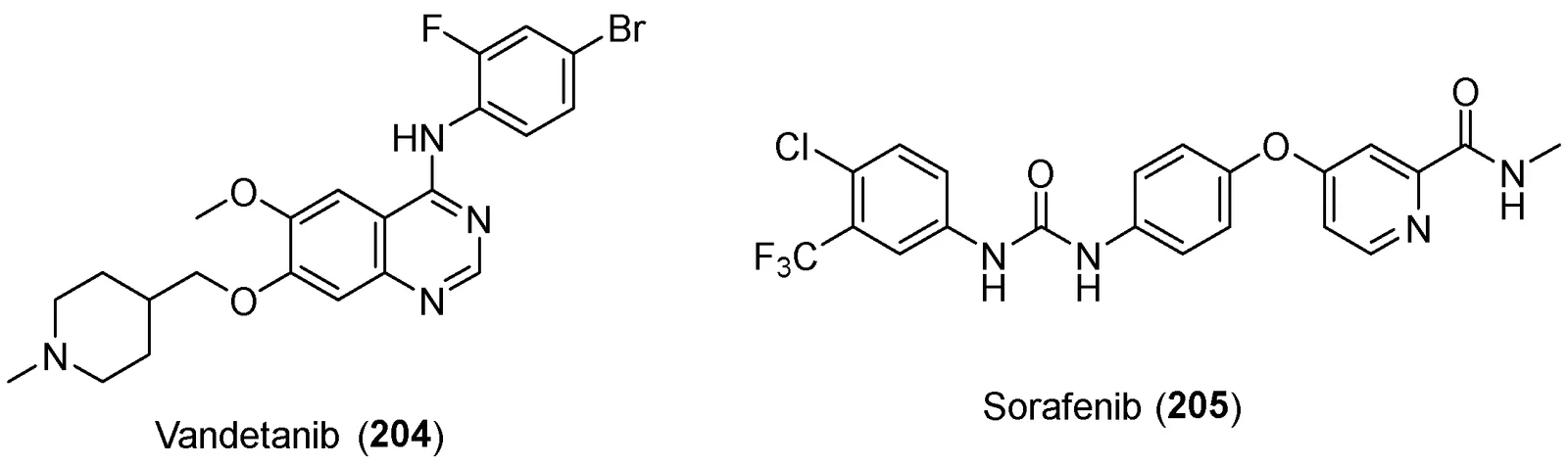
Clinically approved VEGFR-2-targeted kinase inhibitors used as design templates for the development of dual c-Met/VEGFR-2 inhibitors.

Inspired by the success of multitarget kinase inhibitors such as vandetanib **204** [148,149] and sorafenib **205** [150,151] (Figure 14), several groups have used molecular hybridization and pharmacophore-fusion strategies to develop dual c-Met/VEGFR-2 inhibitors. These approaches typically combine a hinge-binding kinase-recognition element with structural features that engage both kinase domains.

**Scheme 27:**
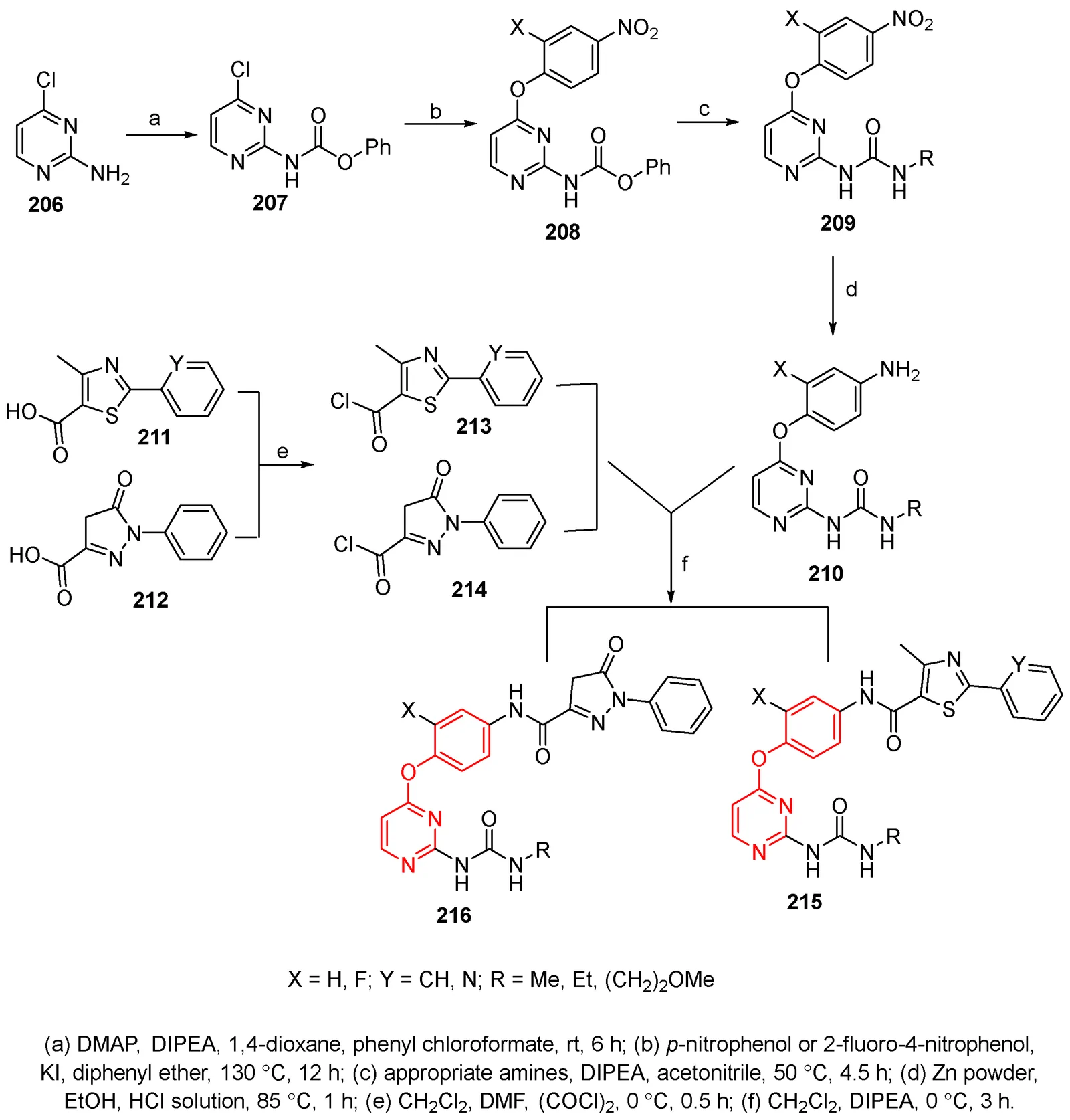
Synthetic route towards 4-phenoxy-pyrimidines **215**/**216**.

Building on the structural features of vandetanib **204**, sorafenib **205**, crizotinib **1**, and foretinib **11**, a series of 4-phenoxy-pyrimidines **215**/**216** was developed as dual c-Met/VEGFR-2 inhibitors (Scheme 27) [152]. Biological evaluation against A549, MCF7, HepG2, and OVCAR-3 cells identified compound **215** (X = H, Y = CH, R = (CH_2_)_2_OMe) as the most promising analog. The compound showed antiproliferative activity comparable to or greater than that of the reference inhibitors across several models and exhibited minimal toxicity toward normal LO2 hepatocytes (IC_50_ > 100 μM).

Mechanistic studies demonstrated dose-dependent G_0_/G_1_-phase arrest and induction of apoptosis in A549 cells. Enzymatic assays confirmed the intended dual-target profile, with IC_50_ values of 1.43 μM for c-Met and 1.05 μM for VEGFR-2. Molecular docking and dynamics simulations further supported productive interactions with both kinase domains, providing evidence that the hybridization strategy successfully generated a balanced dual inhibitor [152].

**Scheme 28:**
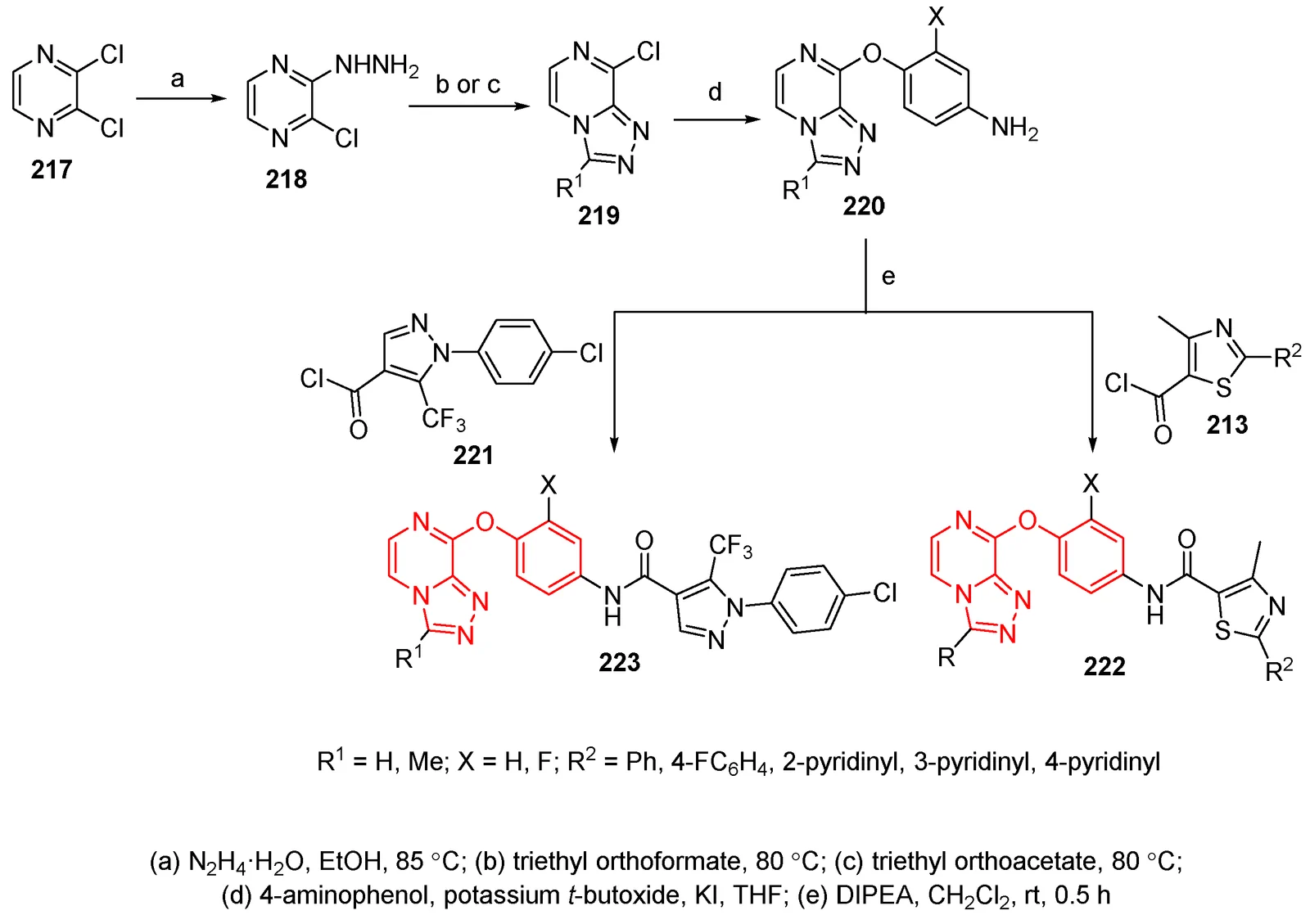
Synthetic route towards [1,2,4]triazolo[4,3-*a*]pyrazines **222**/**223**.

A second approach used a [1,2,4]triazolo[4,3-*a*]pyrazine scaffold (**222**/**223**) derived from foretinib’s pharmacophoric architecture (Scheme 28) [153]. In this design, foretinib’s quinoline hinge-binding region was replaced with a triazolopyrazine core, while the remaining features associated with kinase recognition were retained.

Among the synthesized compounds, **223** (X = F, R = Me) emerged as the lead derivative. The compound showed antiproliferative activity comparable to foretinib across A549, MCF7, and HeLa cells and inhibited c-Met with an IC_50_ of 26 nM, compared with 19 nM for foretinib [153]. It also induced G_0_/G_1_-phase arrest and apoptosis in A549 cells. Docking studies using both c-Met and VEGFR-2 structures supported favorable binding interactions and provided a structural rationale for the observed dual-target activity.

**Scheme 29:**
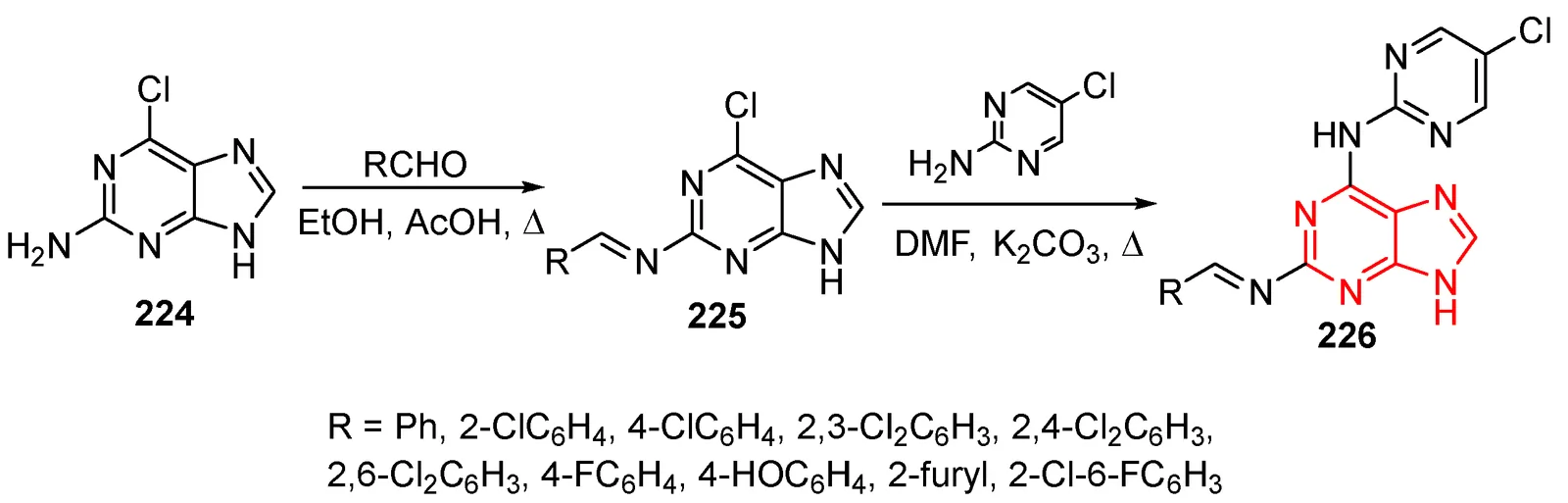
Synthetic route towards 2-(arylideneamino)-*N*-(5-chloropyrimidin-2-yl)-9*H*-purine-6-amines **226**.

A third design strategy employed a purine–pyrimidine hybrid scaffold, yielding a series of 2-(arylideneamino)-N-(5-chloropyrimidin-2-yl)-9*H*-purine-6-amines **226** (Scheme 29) [154]. The most active derivative, bearing a 4-fluorophenyl substituent, showed favorable antiproliferative activity against HT-29 and COLO-205 colorectal cancer cells and potent VEGFR-2 inhibition. Molecular docking suggested productive accommodation within both the c-Met and VEGFR-2 kinase domains. However, direct biochemical c-Met inhibition was not reported, so the proposed dual-target mechanism remains computationally supported rather than experimentally confirmed [154].

Collectively, these studies demonstrate the effectiveness of combining c-Met and VEGFR-2 pharmacophores within a single molecular framework. Pyrimidine-, triazolopyrazine-, and purine-based scaffolds yielded compounds that suppress both tumor-cell signaling and angiogenic pathways. Among the reported series, compounds **215** and **223** provide the strongest experimental support for dual c-Met/VEGFR-2 inhibition, whereas the purine–pyrimidine hybrids **226** remain promising but require direct biochemical validation of c-Met inhibition. The recurring success of phenoxy-linked architectures and hinge-binding nitrogen-rich heterocycles highlights important design principles that will be revisited in the comparative analysis of multitarget c-Met inhibitors (Section 5.8).

### 5.2. c-Met/EGFR Dual Inhibitors

Concurrent inhibition of c-Met and the epidermal growth factor receptor (EGFR) is a promising strategy to overcome tumor progression and acquired resistance associated with single-target kinase inhibitors. EGFR is a major oncogenic driver in many epithelial malignancies [155,156], particularly non-small-cell lung cancer (NSCLC), in which aberrant receptor activation promotes proliferation, survival, invasion, and metastasis. Extensive crosstalk between EGFR and c-Met signaling networks enables tumor cells to bypass EGFR inhibition through compensatory c-Met activation, making dual targeting an attractive approach to improve therapeutic durability and suppress resistance.

**Figure 15:**
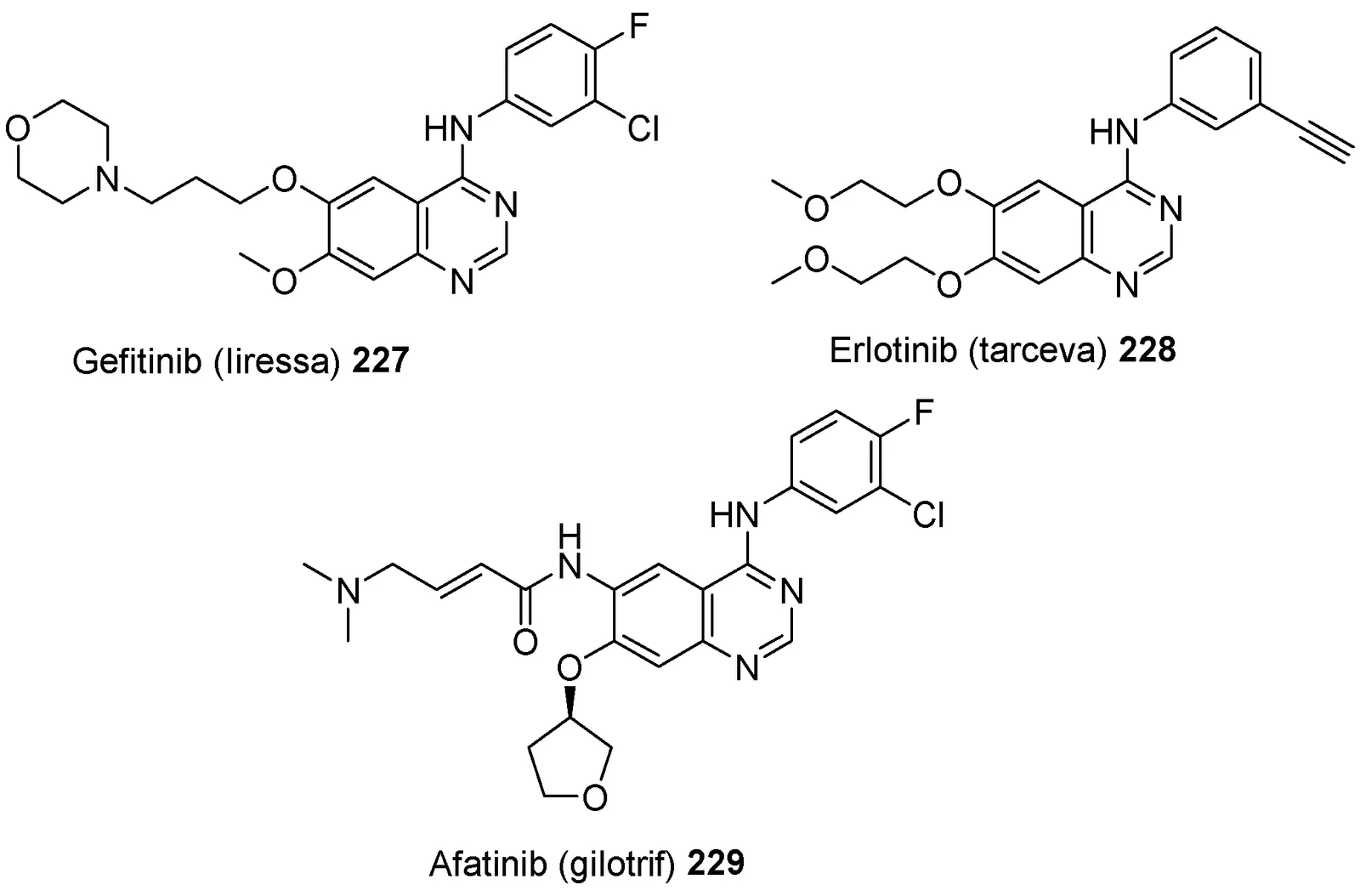
Clinically approved EGFR inhibitors used as design templates for dual c-Met/EGFR inhibitor development.

The clinical success of quinazoline-based EGFR inhibitors, including gefitinib **227** [157,158], erlotinib **228** [159,160], and afatinib **229** [161,162], established the quinazoline nucleus as a privileged EGFR-binding pharmacophore (Figure 15). Consequently, many dual c-Met/EGFR inhibitor programs have focused on combining the quinazoline scaffold with structural features required for c-Met inhibition.

**Figure 16:**
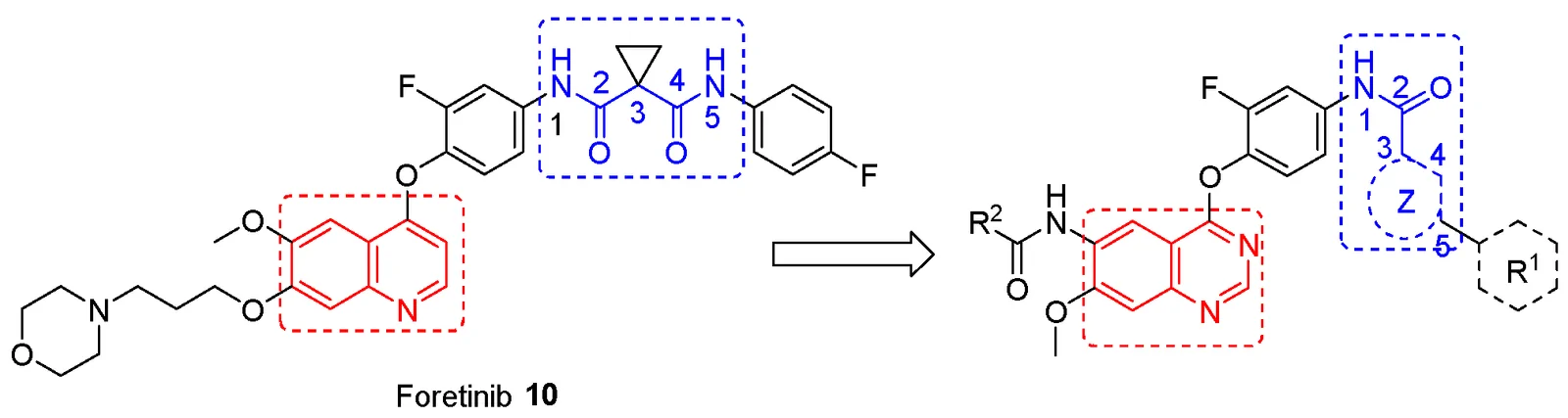
Design strategy for 4-(2-fluorophenoxy)-7-methoxyquinazolines as dual EGFR/c-Met inhibitors.

Inspired by the pharmacophoric features of afatinib and foretinib, a series of 4-(2-fluorophenoxy)-7-methoxyquinazoline derivatives **235** was developed as dual c-Met/EGFR inhibitors (Figure 16) [163]. In this design, the quinoline hinge-binding motif of foretinib was replaced with a quinazoline core to introduce EGFR activity while preserving the characteristic hydrogen-bonding architecture associated with Type II c-Met inhibition.

**Scheme 30:**
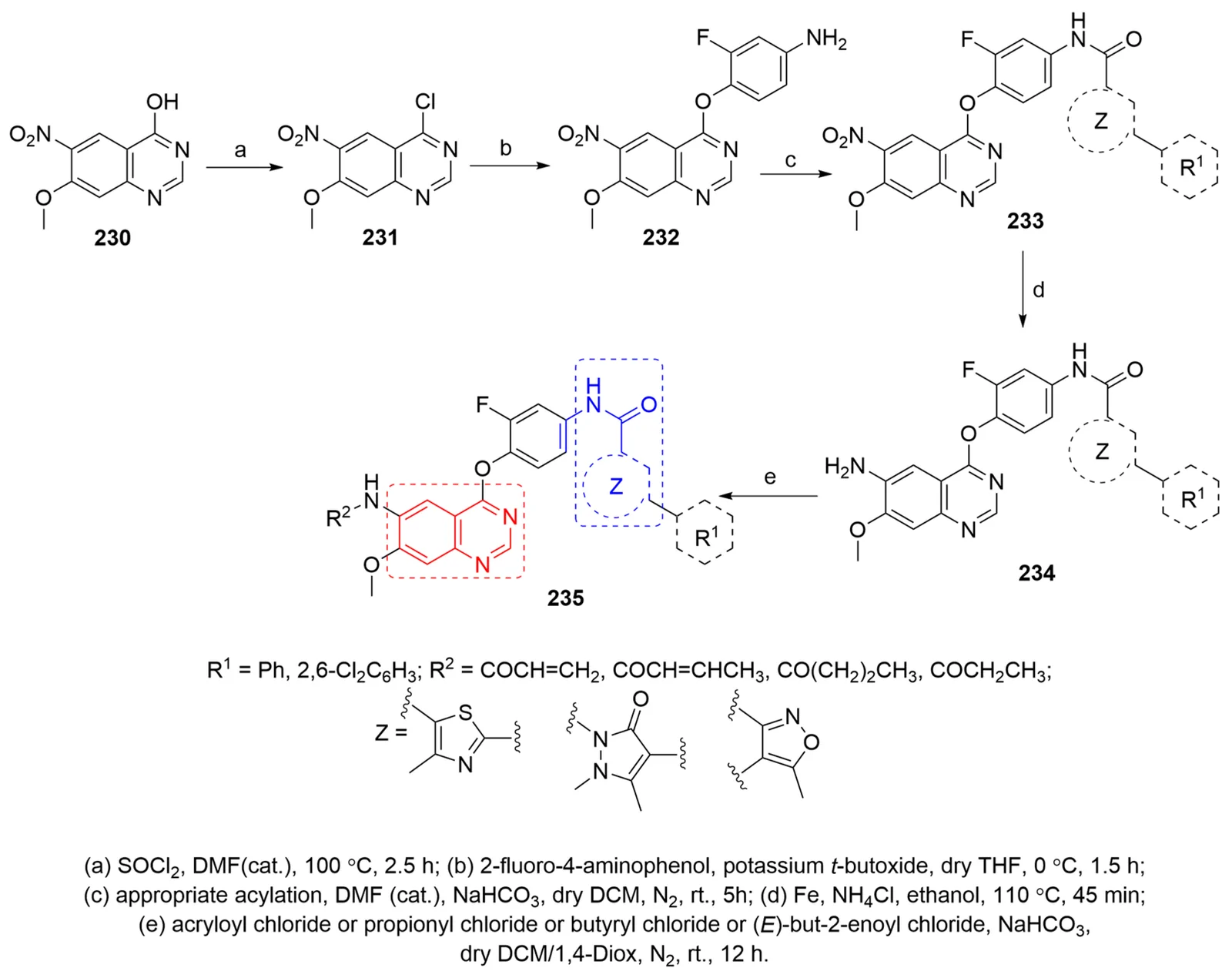
Synthetic route towards 4-(2-fluorophenoxy)-7-methoxyquinazolines **235**.

The target compounds were synthesized via sequential chlorination, nucleophilic aromatic substitution, acylation, nitro reduction, and final coupling reactions (Scheme 30). Biological evaluation against A549-P, H1975, and PC-9 NSCLC cells identified the derivative with R^1^ = Ph, R^2^ = COCH=CH_2_, and Z = 1,5-dimethyl-3-oxo-2,3-dihydro-1*H*-pyrazol-4-yl substituent as the lead compound.

This analog displayed potent antiproliferative activity, with IC_50_ values of 1.06, 1.13, and 1.05 μM against A549-P, H1975, and PC-9 cells, respectively. Enzymatic assays confirmed a balanced dual-target profile, inhibiting EGFR^WT^ (IC_50_ = 99.8 nM), EGFR^L858R^ (IC_50_ = 68.1 nM), and c-Met (IC_50_ = 0.26 nM) [163]. Notably, c-Met activity substantially exceeded that of foretinib under the assay conditions reported. In vivo evaluation in A549-P xenograft-bearing mice demonstrated 55.3% tumor growth inhibition, surpassing that of afatinib. Docking studies of EGFR (PDB ID: 5GMP) and c-Met (PDB ID: 3LQ8) further supported productive interactions with both kinase domains [163].

**Scheme 31:**
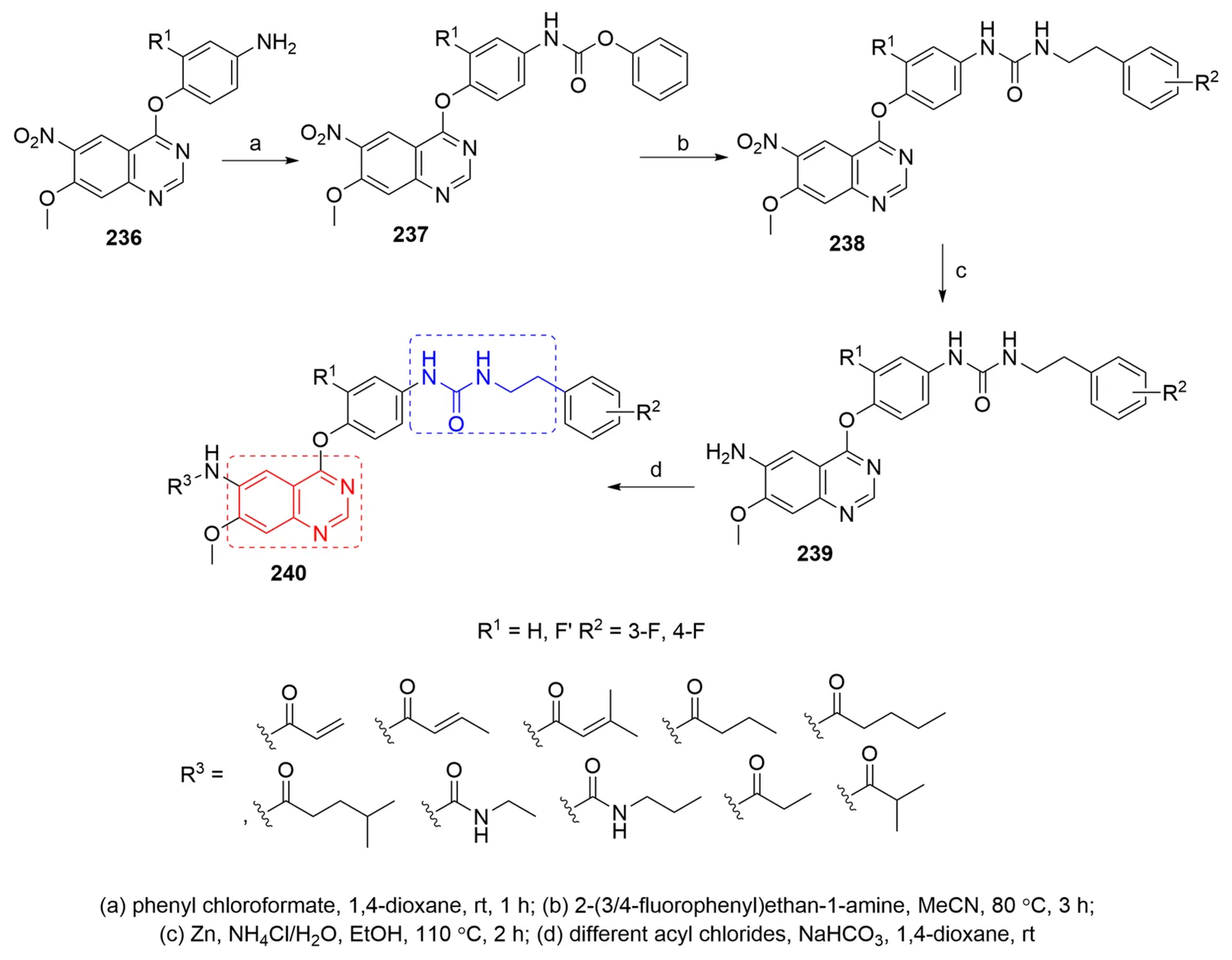
Synthetic route towards 4-phenoxyquinazolines **240**.

A second series of 4-phenoxyquinazoline derivatives **240** was developed using a related design strategy (Scheme 31) [164]. These compounds retained the quinazoline hinge-binding motif while incorporating optimized substituents to balance inhibition of EGFR and c-Met.

Several derivatives showed promising antiproliferative activity against A549, H1975, H460, MCF7, and HeLa cells. The most active analog, with R^1^ = F, R^2^ = 4-F, and an acryloyl substituent, exhibited IC_50_ values of 2.76, 2.27, 2.48, 3.53, and 2.32 μM, respectively [164].

Biochemical analyses showed inhibition of EGFR^WT^ (IC_50_ = 64.8 nM), the resistant EGFR^L858R/T790M^ mutant (IC_50_ = 305.4 nM), and c-Met (IC_50_ = 137.4 nM). This activity against a clinically relevant EGFR resistance mutation underscores the benefit of integrating electrophilic and irreversible inhibitor features into dual-target kinase inhibitors. In vivo tests in mice with A549 xenografts revealed 44.7% suppression of tumor growth, comparable to the effect of afatinib [164].

Dual c-Met/EGFR inhibition is among the most clinically relevant multitarget strategies, particularly in NSCLC, where compensatory c-Met activation frequently drives resistance to EGFR-directed therapy. Across the reported series, the quinazoline scaffold preserved EGFR recognition while accommodating structural features required for c-Met inhibition. Compounds **235** and **240** showed balanced activity against both kinase targets, favorable cellular potency, and encouraging in vivo efficacy. Maintaining activity against mutant EGFR variants while suppressing c-Met signaling further highlights the therapeutic potential of this design strategy to overcome resistance in EGFR-driven cancers.

### 5.3. CDK-2/EGFR/c-Met Triple Inhibitors

Simultaneously inhibiting cyclin-dependent kinase 2 (CDK2), the epidermal growth factor receptor (EGFR), and c-Met is an attractive multitarget strategy for disrupting complementary oncogenic processes. CDK2 is a key regulator of G_1_/S-phase progression and cell-cycle control, whereas EGFR and c-Met drive proliferation, survival, invasion, and metastatic signaling. Because these pathways cooperate in many tumors, concurrently inhibiting cell-cycle progression and growth-factor signaling may yield broader antitumor activity and reduce the likelihood of adaptive resistance.

**Scheme 32:**
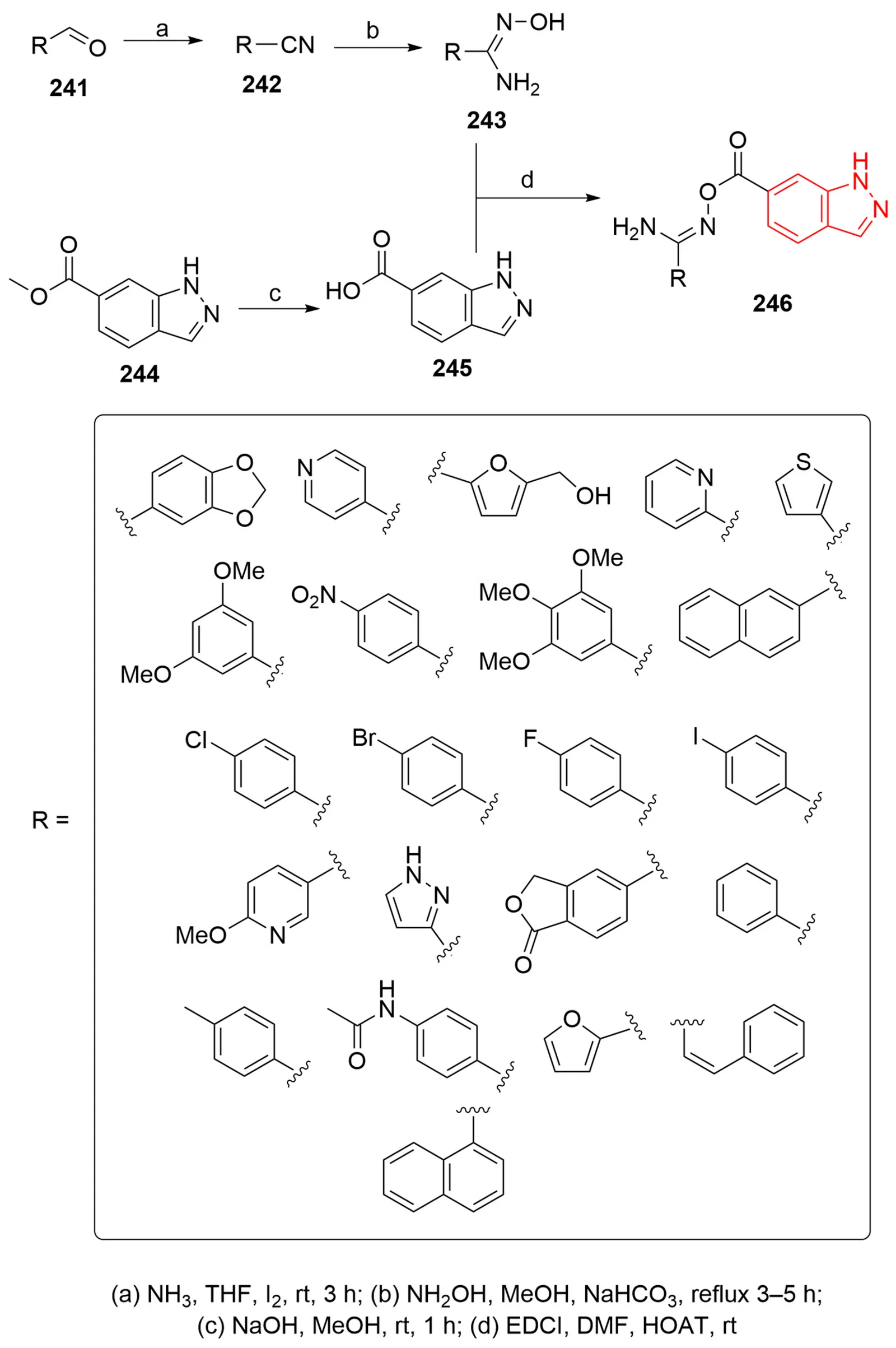
Synthetic route toward indazole derivatives **246**.

To explore this concept, a series of indazole derivatives **246** was designed and synthesized, using an indazole scaffold as the central kinase-binding pharmacophore [165,166]. The target compounds were prepared by coupling 1*H*-indazole-6-carboxylic acid **245** with appropriately substituted amidoximes **243** under EDCI/HOAt-mediated coupling conditions (Scheme 32). The indazole nucleus was selected for its ability to engage kinase ATP-binding sites while allowing the structural diversification required for multitarget recognition.

Biological evaluation against A549 lung, MCF7 breast, Panc-1 pancreatic, and HT-29 colorectal cancer cells identified several highly active derivatives. Compounds with 3,5-dimethoxyphenyl, 6-methoxy-3-pyridinyl, or 4-phenylacetamide substituents were the most promising analogs, showing IC_50_ values of 0.60–1.20 μM across the tested cell lines [166]. The broad antiproliferative activity across multiple tumor types suggests that coordinated inhibition of multiple signaling pathways substantially contributes to their biological effects.

Direct kinase profiling confirmed the intended multitarget mechanism. The lead compounds inhibited CDK2, EGFR, and c-Met, with IC_50_ values of approximately 14–22 nM, 80–89 nM, and 3.10–4.10 nM, respectively [166]. These activities compare favorably with those of the reference inhibitors dinaciclib (CDK2), erlotinib (EGFR), and foretinib (c-Met), demonstrating successful integration of three kinase-targeting pharmacophores into a single molecular framework.

Mechanistic studies further supported the antiproliferative activity of this series. Treatment of MCF7 cells led to significant upregulation of caspase-3, indicating activation of apoptotic pathways and suggesting that programmed cell death contributes to the observed anticancer effects [166].

The indazole-based series **246** demonstrates the feasibility of combining inhibition of cell-cycle regulation (CDK2) with suppression of growth-factor signaling (EGFR and c-Met) in a single molecule. Unlike many dual-target inhibitors that focus exclusively on compensatory kinase pathways, this approach targets both proliferative signaling and cell-cycle progression, providing a broader mechanistic basis for antitumor activity. The balanced nanomolar inhibition of CDK2, EGFR, and c-Met, together with low-micromolar cellular potency, identifies this scaffold as a noteworthy example of rational triple-target kinase inhibitor design and highlights the potential of indazole-based architectures for advanced polypharmacology strategies.

### 5.4. PARP1/c-Met Dual Inhibitors

Poly(ADP-ribose) polymerase 1 (PARP1) is a key DNA-damage response enzyme that detects and repairs single-strand DNA breaks. Although PARP inhibitors have achieved considerable clinical success in ovarian, breast, pancreatic, and prostate cancers, intrinsic and acquired resistance remain major limitations. Increasing evidence suggests that aberrant c-Met signaling can contribute to PARP inhibitor resistance by promoting compensatory survival pathways and enhancing tumor-cell adaptability. Consequently, simultaneous inhibition of PARP1 and c-Met has emerged as a promising strategy to enhance therapeutic efficacy and overcome resistance.

**Scheme 33:**
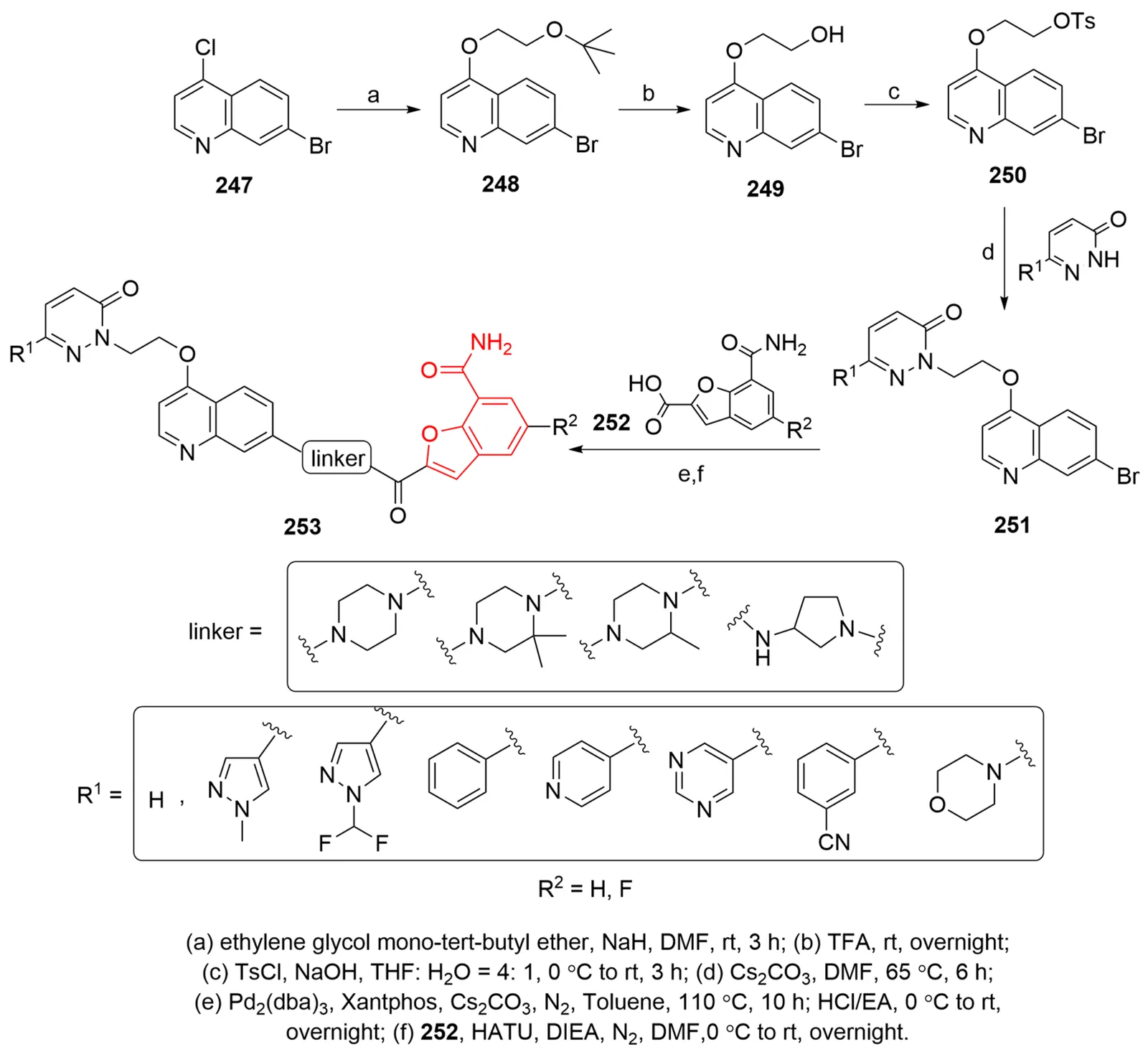
Synthetic route towards benzofuran-7-carboxamide containing compounds **253**.

To address this challenge, a series of benzofuran-7-carboxamide derivatives **253** was developed as dual PARP1/c-Met inhibitors (Scheme 33) [167]. The design integrated a PARP1-targeting benzofuran carboxamide pharmacophore with structural features associated with c-Met inhibition, thereby combining DNA-repair blockade and kinase inhibition in a single molecule. The target compounds were synthesized from 7-bromo-4-chloroquinoline **247** through a multistep sequence that afforded the key intermediate **251**, which was then coupled with substituted benzofuran-7-carboxamides **252** using either piperazinyl or 3-aminopyrrolidinyl linkers.

**Figure 17:**
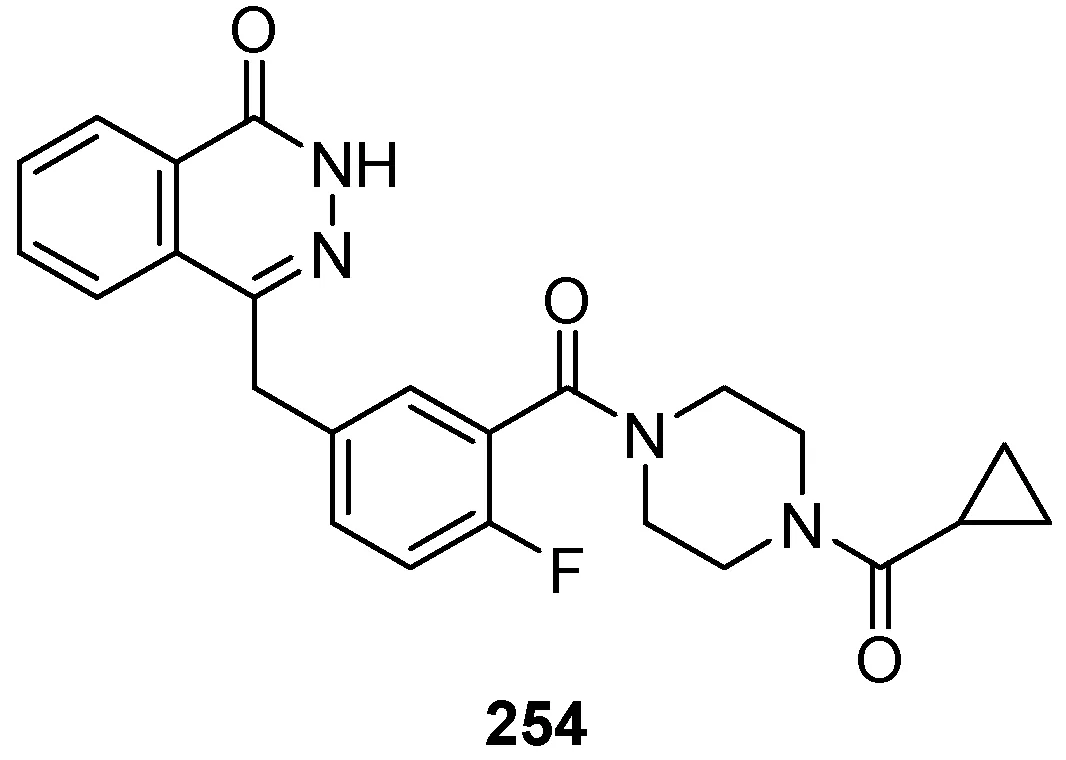
Chemical structure of the FDA-approved PARP inhibitor olaparib **254**.

Biological evaluation in the olaparib-resistant HCT116OR colorectal cancer model identified the derivative bearing a 3-tolyl substituent (R^2^ = H) and a piperazinyl linker as the lead compound [167]. The compound inhibited HCT116OR cell proliferation with an IC_50_ of 4.05 μM, markedly outperforming the clinically approved PARP inhibitor olaparib **254** [168] (Figure 17), which exhibited an IC_50_ of 50.82 μM under the same experimental conditions.

Direct enzymatic assays confirmed the intended dual-target profile. The lead compound inhibited PARP1 and c-Met, with IC_50_ values of 21.8 nM and 30.23 nM, respectively, demonstrating balanced nanomolar activity against both targets [167]. These results validate the multitarget design strategy and support the simultaneous disruption of DNA repair and oncogenic signaling pathways.

Importantly, the compound also demonstrated significant in vivo efficacy in a Balb/c nude mouse xenograft model established with HCT116OR cells. Administration at 25 mg/kg/day and 50 mg/kg/day for 21 days resulted in tumor growth inhibition (TGI) of 67% and 72%, respectively [167]. Under comparable conditions, olaparib and crizotinib produced substantially lower antitumor effects, highlighting the potential therapeutic advantage of the dual-target approach in resistant tumors.

Molecular docking studies using the PARP1 (PDB ID: 7KK5) and c-Met (PDB ID: 3CD8) structures identified favorable interactions with both targets and provided a structural rationale for the observed biological activity [167]. Because direct enzymatic inhibition of both PARP1 and c-Met was experimentally demonstrated, the computational studies provide mechanistic support rather than primary evidence of target engagement.

The benzofuran-7-carboxamide series exemplifies rational multitarget design beyond kinase–kinase combinations. By simultaneously inhibiting PARP1-mediated DNA repair and c-Met-driven survival signaling, these compounds target a clinically relevant resistance mechanism while maintaining balanced nanomolar potency against both targets. Robust cellular activity in an olaparib-resistant model, together with encouraging xenograft efficacy, suggests that PARP1/c-Met dual inhibition may be a promising strategy to overcome resistance to DNA damage response therapies and to expand the therapeutic utility of PARP inhibitors.

### 5.5. Mer/c-Met Dual Inhibitors

MER proto-oncogene tyrosine kinase (MERTK) is a member of the TAM receptor tyrosine kinase family, which also includes TYRO3 and AXL. Under physiological conditions, MER regulates immune homeostasis, efferocytosis, and tissue repair. In cancer, aberrant MER activation has been linked to tumor-cell survival, immune evasion, migration, metastasis, and therapeutic resistance. Because co-activation of MER and c-Met signaling frequently drives tumor progression and poor clinical outcomes, concurrent inhibition of both kinases has emerged as an attractive strategy to suppress complementary oncogenic pathways and reduce the likelihood of resistance associated with single-target therapies [169].

**Scheme 34:**
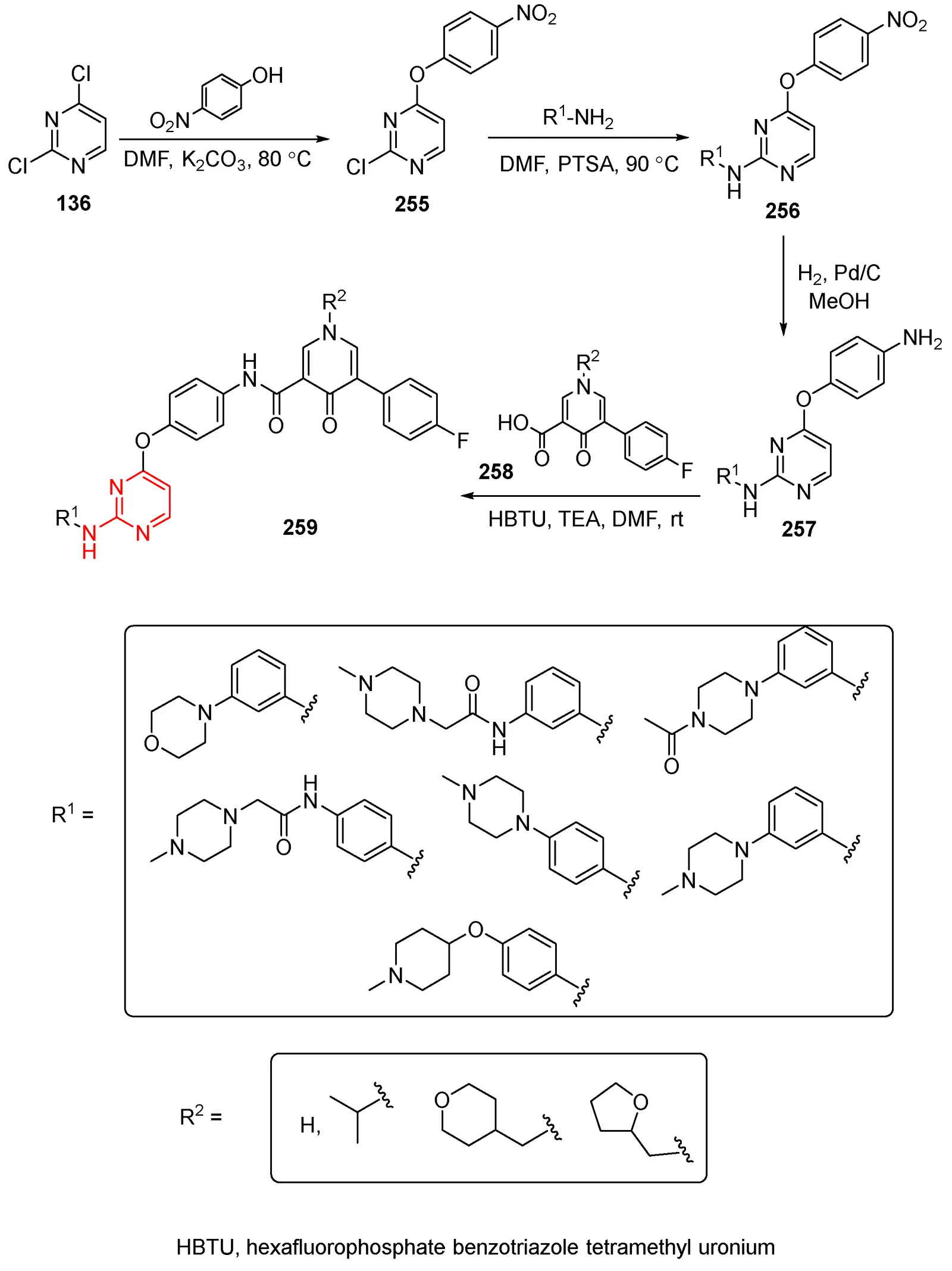
Synthetic route towards Mer/c-Met inhibitors **259**.

To explore this approach, a series of pyrimidine-based dual MER/c-Met inhibitors **259** was developed using a rational kinase-inhibitor design strategy (Scheme 34) [169]. The target compounds were synthesized by sequentially functionalizing a 2,4-dichloropyrimidine scaffold, installing aminophenoxy intermediates, and finally coupling with substituted 4-oxo-1,4-dihydropyridine-3-carboxylic acid derivatives. The resulting architecture incorporated structural elements capable of recognizing the ATP-binding sites of both MER and c-Met.

Biological evaluation identified a lead compound with a 4-(1-methylpiperazin-4-yl)phenyl substituent (R^1^) and a 2-methyltetrahydrofuran group (R^2^). This derivative showed potent dual-target activity, inhibiting MER and c-Met with IC_50_ values of 1.0 nM and 19.0 nM, respectively [169]. Although its c-Met activity was lower than that of cabozantinib in the reported enzyme assay, the compound demonstrated superior cellular performance.

Antiproliferative evaluation against HCT116 colon, A2780 ovarian, and PC-3 prostate cancer cells yielded IC_50_ values of 1.01, 0.96, and 3.42 μM, respectively, outperforming cabozantinib across all tested cell lines [169]. Molecular docking studies using MER (PDB ID: 4M3Q) and c-Met (PDB ID: 3LQ8) structures supported productive binding interactions in both kinase domains and provided a structural rationale for the observed dual inhibitory activity.

**Scheme 35:**
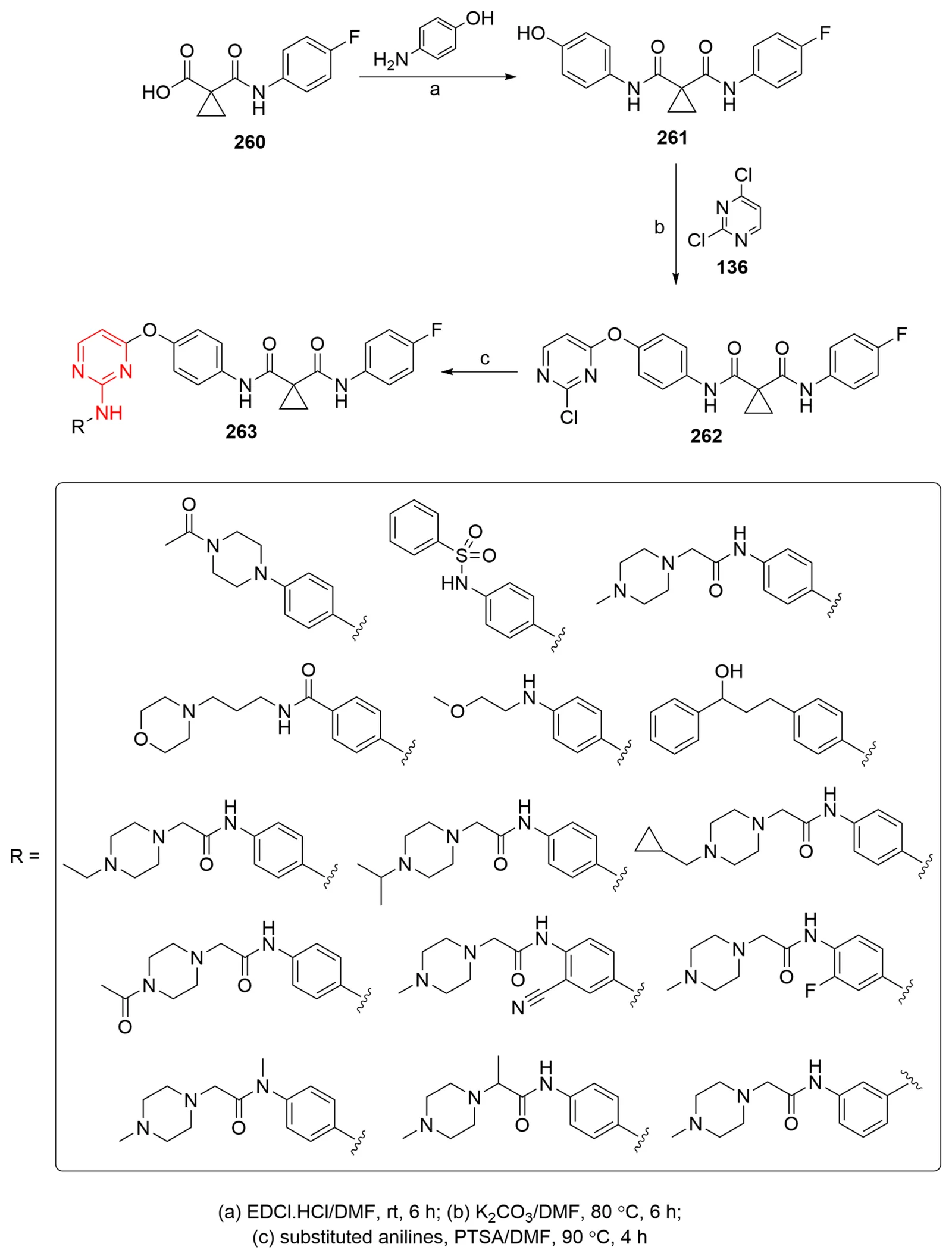
Synthetic route towards Mer/c-Met inhibitors **263**.

A second series of pyrimidine-based dual inhibitors **263** was developed to further optimize target engagement and biological activity (Scheme 35) [170]. In this series, the incorporation of a cyclopropanecarboxamide-containing pharmacophore was intended to improve kinase recognition while preserving the dual-target design.

Several analogs showed measurable activity against both kinases. The most active compound, bearing a 2-(4-methylpiperazin-1-yl)-N-(4-phenyl)acetamide substituent, inhibited MER and c-Met with IC_50_ values of 18.5 nM and 33.6 nM, respectively [170]. Although less potent than the lead compound from series **259**, it retained balanced dual-target activity.

The compound also exhibited antiproliferative activity against HepG2 liver, MDA-MB-231 breast, and HCT116 colon cancer cells, with IC_50_ values of 6.63, 8.90, and 4.95 μM, respectively [170]. Mechanistic studies demonstrated induction of apoptosis and significant inhibition of HCT116 cell migration in Transwell assays, indicating potential effects on both tumor growth and metastatic behavior. Docking studies using MER (PDB ID: 4M3Q) and c-Met (PDB ID: 3LQ8) crystal structures further supported the experimentally observed dual-target profile [170].

Dual MER/c-Met inhibition is a rational strategy for simultaneously targeting tumor-cell survival, metastatic progression, and resistance-associated signaling. Across the reported studies, the pyrimidine scaffold proved highly effective at enabling balanced recognition of both kinase targets. In particular, compound **259** demonstrated an attractive combination of subnanomolar MER inhibition, nanomolar c-Met inhibition, and strong cellular activity, highlighting the value of integrating TAM-family and c-Met-directed pharmacophores within a single molecular framework. These findings further support the broader concept that rational polypharmacology can enhance biological activity even when individual target potency does not exceed that of established single-target inhibitors.

### 5.6. Tubulin Polymerization/c-Met Dual Inhibitors

Microtubules are essential cytoskeletal components that regulate mitotic spindle formation, chromosome segregation, intracellular transport, and maintenance of cellular architecture. Because microtubule dynamics are indispensable for cell division, disrupting tubulin polymerization has long been an effective anticancer strategy. However, resistance and dose-limiting toxicities associated with classical microtubule-targeting agents have spurred the development of multitarget approaches that simultaneously disrupt cytoskeletal function and oncogenic signaling. In this context, concurrent inhibition of tubulin polymerization and c-Met is an attractive strategy for enhancing antitumor activity while reducing the likelihood of resistance [171].

**Scheme 36:**
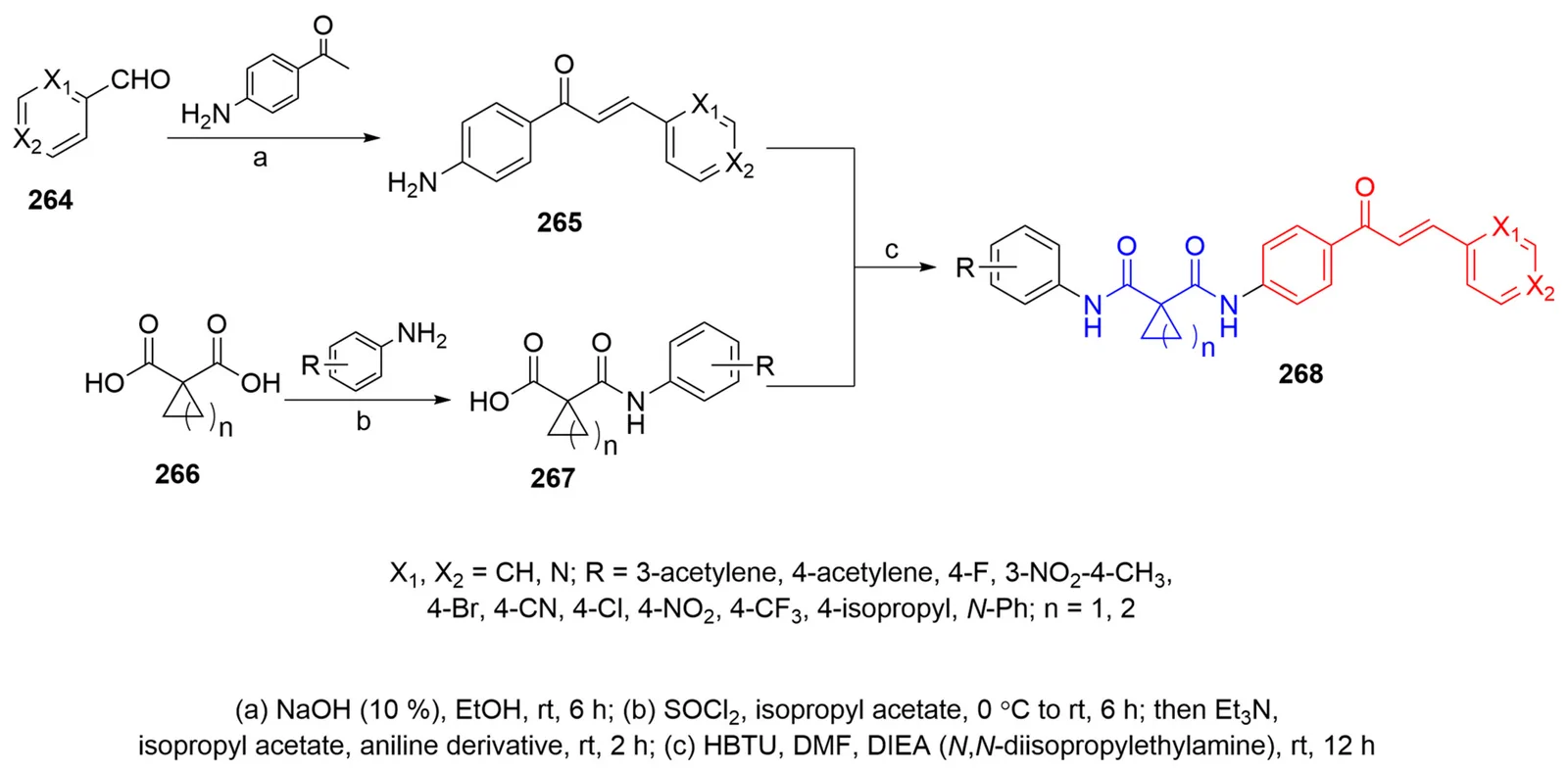
Synthetic route towards cycloalkyl diacetamide-bearing chalcones **268**.

To explore this concept, a series of cycloalkyl diacetamide-bearing chalcones **268** was designed and synthesized as dual tubulin/c-Met inhibitors (Scheme 36) [171]. The design combined the chalcone pharmacophore, a well-established tubulin-binding motif, with structural features associated with c-Met inhibition. The target compounds were prepared via Claisen–Schmidt condensation to afford chalcone intermediates, which were then coupled with cycloalkyl diacetamide-containing fragments.

Biological evaluation revealed promising antiproliferative activity against HeLa cervical and PC-3 prostate cancer cells. Two compounds emerged as the most active derivatives: one with X_1_ = N, X_2_ = CH, R = 4-Br, n = 2, and another with X_1_ = CH, X_2_ = N, R = 4-isopropyl, n = 1 [171]. These compounds exhibited IC_50_ values of 3.90 and 4.50 μM against HeLa cells and 0.22 and 0.54 μM against PC-3 cells, respectively. Notably, the most active analog showed greater activity against PC-3 cells than the reference c-Met inhibitor, foretinib, under the assay conditions reported.

Direct biochemical evaluation confirmed potent c-Met inhibition, with IC_50_ values of 24 nM and 45 nM for the two lead compounds, respectively, compared with 14 nM for foretinib [171]. Although their tubulin-polymerization inhibitory activity was less potent than that of classical microtubule-disrupting agents such as paclitaxel or colchicine, the combination of c-Met inhibition and interference with microtubule dynamics likely contributed to the observed cellular potency.

Molecular docking studies using the c-Met crystal structure (PDB ID: 3LQ8) identified productive interactions within the ATP-binding pocket and provided a structural rationale for the experimentally observed kinase inhibition [171]. Because direct biochemical c-Met activity was confirmed, the docking studies primarily serve as mechanistic support for the dual-target design.

The cycloalkyl diacetamide-bearing chalcone series demonstrates the feasibility of combining cytoskeletal disruption with kinase inhibition in a single molecular framework. While c-Met inhibition appears to be the primary driver of anticancer activity, modulating tubulin dynamics may further enhance therapeutic efficacy and reduce reliance on a single mechanism of action. These findings illustrate how integrating kinase-targeting and microtubule-targeting pharmacophores can generate multifunctional anticancer agents and further expand the scope of rational multitarget c-Met inhibitor design.

### 5.7. c-Met/Axl Dual Inhibitors

AXL is a member of the TAM (TYRO3, AXL, and MER) receptor tyrosine kinase family and is frequently overexpressed in a variety of solid tumors, where it promotes tumor-cell proliferation, epithelial–mesenchymal transition (EMT), invasion, metastasis, immune evasion, and resistance to targeted therapies. Increasing evidence indicates that concurrent activation of AXL and c-Met signaling drives aggressive tumor behavior and therapeutic resistance, particularly in cancers that develop resistance to receptor tyrosine kinase inhibitors. Consequently, simultaneous inhibition of AXL and c-Met has emerged as a promising strategy to suppress complementary oncogenic pathways while reducing reliance on single-target therapeutics [172].

**Scheme 37:**
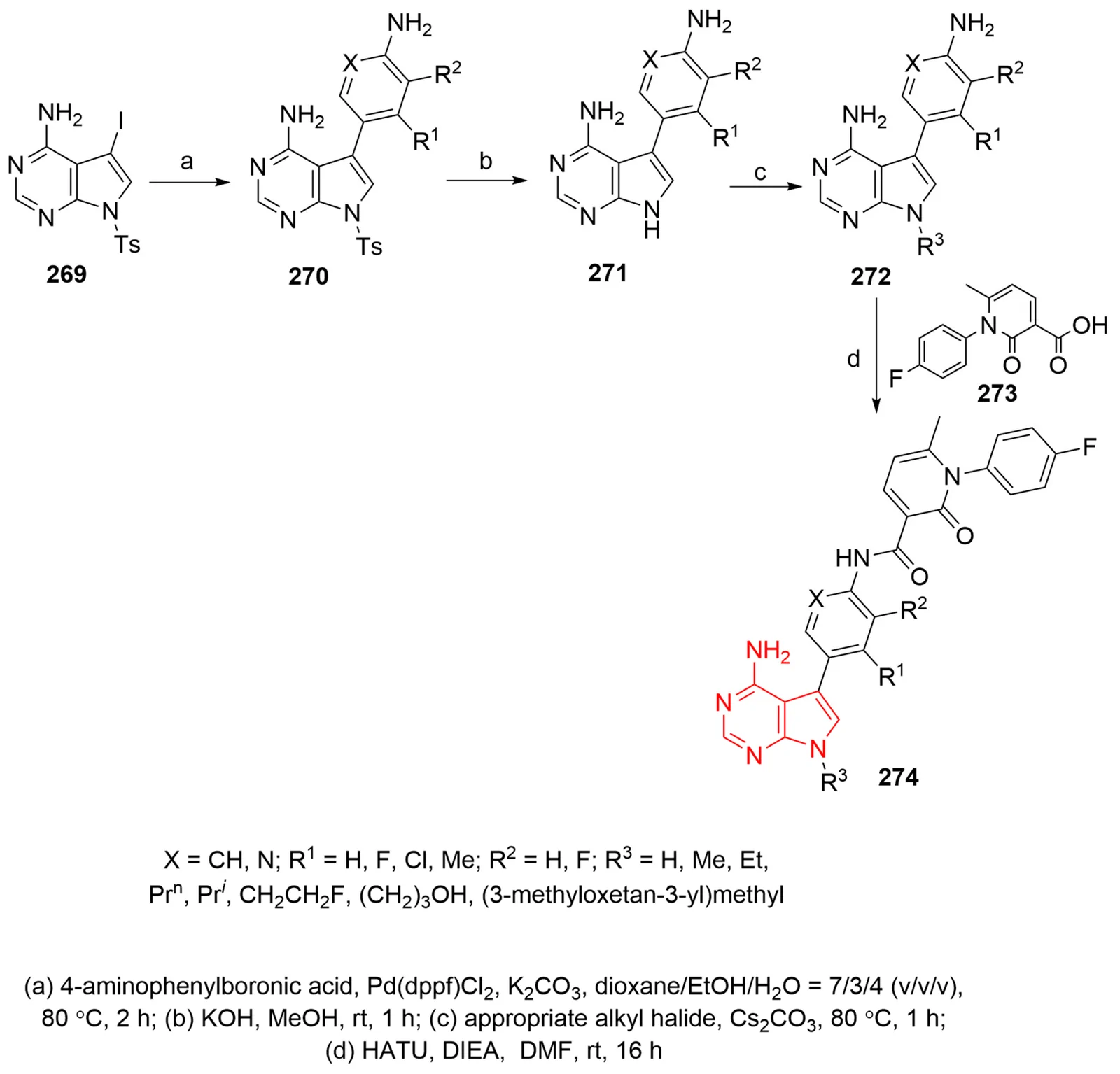
Synthetic route towards 4-amino-7*H*-pyrrolo[2,3-*d*]pyrimidines **274**.

To exploit this approach, a series of 4-amino-7*H*-pyrrolo[2,3-*d*]pyrimidine derivatives **274** was designed and synthesized as dual c-Met/AXL inhibitors (Scheme 37) [172]. The design combined the established kinase-recognition properties of the pyrrolo[2,3-*d*]pyrimidine scaffold with structural modifications intended to achieve balanced inhibition of both receptor tyrosine kinases. The target compounds were prepared via Suzuki cross-coupling of 5-iodo-7-tosyl-7*H*-pyrrolo[2,3-*d*]pyrimidin-4-amine **269**, followed by detosylation, alkylation, and final amide coupling with a 1-(4-fluorophenyl)-6-methyl-2-oxo-1,2-dihydropyridine-3-carboxylic acid derivative **273**.

Biochemical evaluation demonstrated potent dual-target activity. The lead compound, with X = CH, R^1^ = R^3^ = H, and R^2^ = F, inhibited AXL and c-Met with IC_50_ values of 15 nM and 7 nM, respectively, compared with 8 nM and 4 nM for cabozantinib under the same assay conditions [172]. Although slightly less potent than cabozantinib at the enzymatic level, the compound showed superior cellular and in vivo performance.

The lead derivative exhibited broad-spectrum antiproliferative activity across multiple cancer cell lines, including NSCLC models H1975, H460, and A549, with IC_50_ values of 3.3, 5.8, and 0.52 μM, respectively [172]. Similar activity was observed in HepG2, Hep3B, Sk-hep-1, MHCC-97H, and HCT116 cells, consistently matching or exceeding cabozantinib’s activity. These findings suggest that balanced inhibition of both c-Met and AXL may confer advantages beyond maximal potency against either kinase alone.

Importantly, the compound demonstrated remarkable in vivo efficacy. Oral administration at just 1 mg/kg achieved 98.2% tumor-growth inhibition in MKN-45 gastric cancer xenografts and 87.2% in HCT116 colorectal cancer xenografts [172]. This activity highlights the therapeutic potential of simultaneously suppressing c-Met- and AXL-mediated signaling pathways that contribute to tumor progression and resistance.

Molecular docking studies using the crystal structures of AXL (PDB ID: 5U6B) and c-Met (PDB ID: 6SDD) revealed productive interactions within both ATP-binding pockets and provided a structural rationale for the experimentally observed dual-target activity [172].

Dual c-Met/AXL inhibition is among the most compelling multitarget strategies to combat therapeutic resistance and metastatic progression. The pyrrolo[2,3-*d*]pyrimidine scaffold enabled potent, balanced inhibition of both kinases and delivered strong cellular activity and exceptional in vivo activity. Notably, the lead compound demonstrated that superior biological performance can be achieved even without maximizing enzyme potency relative to established inhibitors, underscoring the importance of balanced target coverage, cellular activity, and pharmacological behavior in multitarget drug design. These findings further support c-Met/AXL dual inhibitors as promising candidates for treating aggressive and therapy-resistant malignancies.

### 5.8. Comparative Principles in Multitarget c-Met Inhibitor Design

The multitarget inhibitors discussed in this review illustrate the growing transition from highly selective kinase inhibition toward rational polypharmacology. Although the target combinations varied considerably, several recurring medicinal chemistry principles emerged across successful dual- and multitarget c-Met inhibitor programs. These design strategies seek to preserve potent c-Met inhibition while simultaneously modulating complementary signaling pathways involved in tumor proliferation, angiogenesis, metastasis, DNA repair, cell-cycle progression, immune evasion, and therapeutic resistance. Collectively, these approaches reflect the increasing recognition that the complexity and adaptability of cancer signaling networks often require simultaneous intervention at multiple molecular nodes.

A common characteristic of many multitarget c-Met inhibitors is the incorporation of a hinge-binding heterocyclic core that maintains productive interactions within the c-Met ATP-binding site while accommodating structural features necessary for recognition of additional therapeutic targets. Nitrogen-rich heterocycles such as pyrimidines, quinazolines, pyrrolo[2,3-*d*]pyrimidines, and triazolopyrazines have proven particularly valuable because they provide robust ATP-mimetic properties and permit extensive structural diversification. These scaffolds frequently preserve c-Met affinity while simultaneously supporting interactions with kinases such as VEGFR-2, EGFR, AXL, and MER. Across the reviewed studies, multitarget inhibitors were commonly generated using molecular hybridization, pharmacophore fusion, scaffold hopping, or linker-based integration strategies. These complementary approaches enabled the incorporation of multiple target-recognition elements into a single molecular framework while avoiding excessive molecular complexity and maintaining acceptable drug-like properties.

Several recurring structural motifs were associated with successful multitarget activity. Nitrogen-containing hinge-binding heterocycles remained central to target recognition, whereas 4-phenoxy linker architectures frequently facilitated productive spatial orientation of pharmacophoric elements, particularly in c-Met/VEGFR-2 and c-Met/EGFR inhibitor series. Amide and carboxamide functionalities often served as key hydrogen-bonding motifs capable of interacting with residues such as Asp1222 and Lys1110, while fluorinated aromatic substituents repeatedly contributed to improved potency and cellular activity. In addition, piperazine- and morpholine-containing side chains commonly enhanced physicochemical properties and cellular exposure, whereas hydrophobic terminal aromatic groups promoted occupancy of secondary kinase pockets. Importantly, no single structural feature was sufficient to guarantee multitarget activity. Rather, biological performance depended on the precise spatial arrangement of pharmacophoric elements and their ability to simultaneously engage multiple targets while maintaining favorable physicochemical and pharmacokinetic properties.

The multitarget inhibitors reviewed in this article can be broadly classified according to their biological objectives (Table 4). Among these strategies, dual c-Met/VEGFR-2 inhibitors were primarily developed to simultaneously suppress tumor-cell proliferation and angiogenesis, whereas c-Met/EGFR inhibitors were designed to overcome compensatory signaling mechanisms and acquired resistance in EGFR-driven malignancies. More recently, investigators have expanded beyond kinase–kinase combinations to include c-Met/PARP1 inhibitors that integrate blockade of DNA repair with inhibition of oncogenic survival signaling. Additional approaches targeting c-Met together with MER, AXL, CDK2, or tubulin have sought to suppress metastatic progression, immune evasion, cell-cycle dysregulation, and resistance-associated pathways. Although c-Met/VEGFR-2 and c-Met/EGFR inhibitors currently represent the most clinically advanced multitarget strategies, emerging combinations involving PARP1, MER, AXL, and tubulin demonstrate the expanding scope of rational polypharmacology in c-Met-directed drug discovery.

An important lesson emerging from recent multitarget c-Met inhibitor programs is that balanced target coverage is often more important than maximizing potency against any single target. Several compounds showed superior cellular or in vivo antitumor activity despite slightly weaker biochemical inhibition of c-Met than established reference agents. This phenomenon was particularly evident in the MER/c-Met and AXL/c-Met inhibitor series, where coordinated suppression of complementary signaling pathways elicited greater biological responses than predicted by enzyme inhibition data alone. Similarly, compounds exhibiting comparable kinase IC_50_ values often displayed markedly different antiproliferative activities, highlighting the imperfect relationship between biochemical potency and therapeutic performance.

These findings underscore that successful multitarget inhibitor development requires evaluation beyond isolated enzyme assays. Factors such as cellular permeability, ATP competition, protein binding, metabolic stability, and target engagement in cells all affect outcomes. Future work on multitarget c-Met inhibitors should combine lab potency with thorough checks of kinase selectivity, target engagement in cells, pathway effects, apoptosis, cell-cycle changes, pharmacokinetics, pharmacodynamics, activity in resistant cancer models, and efficacy and tolerability in animals. This broad approach is needed to turn rational multitarget designs into effective c-Met cancer treatments.

## 6. MET-Targeted Protein Degradation Strategies: PROTACs and Emerging Degraders

Targeted protein degradation has emerged as a transformative therapeutic modality that extends beyond the limitations of conventional occupancy-driven small-molecule inhibition. Unlike traditional c-Met kinase inhibitors, which require sustained target engagement to suppress enzymatic activity, targeted protein degraders selectively eliminate the target protein through endogenous protein quality-control systems, most notably the ubiquitin–proteasome pathway. This catalytic mechanism of action offers several potential advantages, including prolonged target suppression, elimination of both kinase-dependent and kinase-independent functions, and the potential to overcome resistance mechanisms that compromise conventional ATP-competitive inhibitors. Consequently, targeted protein degradation has gained increasing attention as a next-generation strategy for c-Met-directed cancer therapy [173,174,175].

Proteolysis-targeting chimeras (PROTACs) are the most studied type of targeted protein degraders. They are made of a target-binding part, a section that recruits an E3 ubiquitin ligase, and a chemical linker joining them. When both the target protein and E3 ligase are brought together, the target is tagged for degradation by the proteasome. Unlike traditional inhibitors that block protein function only temporarily, PROTACs degrade the protein entirely, which may lead to longer-lasting effects and reduce the chance of resistance due to incomplete inhibition [176,177,178].

The biological rationale for targeting c-Met with targeted protein degradation is particularly compelling. Aberrant c-Met signaling drives numerous oncogenic processes, including tumor-cell proliferation, survival, invasion, metastasis, epithelial–mesenchymal transition, angiogenesis, and therapeutic resistance [9,10,11,16]. Furthermore, acquired resistance to clinically approved c-Met inhibitors frequently arises from kinase-domain mutations, particularly substitutions involving Asp1228 and Tyr1230, which impair inhibitor binding while preserving receptor activity [25,26,27,28,46,60]. Because protein degraders eliminate the target protein rather than merely suppressing catalytic activity, they may retain efficacy in settings where conventional ATP-competitive inhibitors become ineffective. In addition, degradation-based approaches can disrupt non-catalytic scaffolding functions of c-Met that may contribute to tumor progression independently of kinase activity.

Although c-Met-targeted degradation remains at a considerably earlier stage of development than degrader programs directed toward androgen receptor, estrogen receptor, BTK, or EGFR, several proof-of-concept studies have demonstrated the feasibility of selective c-Met degradation using PROTAC-based approaches [176,177,178]. Most reported c-Met degraders employ established c-Met inhibitor pharmacophores linked to ligands that recruit cereblon (CRBN) or von Hippel–Lindau (VHL) E3 ligases [173,176]. These molecules have been shown to induce efficient c-Met degradation, suppress downstream PI3K/AKT and MAPK signaling pathways, and inhibit proliferation in c-Met-dependent cancer models. Collectively, these findings support the suitability of c-Met as a target for degradation-based therapeutics and establish a foundation for future medicinal chemistry optimization.

From a medicinal chemistry perspective, the development of c-Met degraders presents challenges distinct from those associated with conventional kinase inhibitors. Beyond maintaining adequate target affinity, successful degraders must promote productive ternary-complex formation while retaining physicochemical properties compatible with cellular permeability, metabolic stability, and favorable pharmacokinetic behavior. The relatively large molecular size and increased polarity characteristic of many PROTACs often result in reduced oral bioavailability and limited intracellular exposure. Consequently, linker composition, linker length, attachment vectors, E3 ligase selection, and ternary-complex cooperativity have emerged as critical optimization parameters. Future studies will need to establish robust structure–degradation relationships alongside traditional structure–activity relationships to identify degrader candidates with improved drug-like properties.

Beyond PROTACs, several emerging degradation technologies may further expand therapeutic opportunities for c-Met-directed intervention. Molecular glues represent a mechanistically distinct class of degraders that promote target degradation by stabilizing interactions between endogenous E3 ligases and target proteins without requiring bifunctional architectures [179]. Additional modalities, including lysosome-targeting chimeras (LYTACs) [180], autophagy-targeting chimeras (AUTACs) [181], and autophagosome-tethering compounds (ATTECs) [182], have broadened the scope of targeted protein degradation and may ultimately provide alternative approaches for eliminating membrane-associated receptor tyrosine kinases such as c-Met. Although these technologies remain largely unexplored in c-Met biology, they represent promising directions for future investigation.

The integration of targeted protein degradation with precision oncology may significantly influence the next generation of c-Met-directed therapeutics. Opportunities exist to develop mutation-selective degraders, dual-target degraders that simultaneously suppress c-Met and resistance-associated signaling pathways, and biomarker-guided therapeutic strategies tailored to molecularly defined patient populations. As advances continue in structural biology, protein–protein interaction analysis, chemical biology, and computational drug design, targeted protein degradation is expected to become an increasingly important component of c-Met-directed drug discovery and may provide innovative solutions to challenges that currently limit the long-term success of conventional kinase inhibitors.

## 7. Conclusions and Future Perspectives

The HGF/c-Met signaling axis remains a well-validated therapeutic target in oncology because of its central role in tumor growth, survival, invasion, metastasis, angiogenesis, and therapeutic resistance. The clinical success of agents such as crizotinib, capmatinib, tepotinib, savolitinib, and cabozantinib has firmly established c-Met as a druggable oncogenic driver and demonstrated the therapeutic value of c-Met-directed intervention in molecularly defined cancers.

Recent advances in medicinal chemistry, structural biology, and structure-based drug design have significantly broadened the repertoire of c-Met inhibitors, yielding diverse heterocyclic scaffolds with improved potency, selectivity, and pharmacological profiles. Across these scaffold classes, several recurring medicinal chemistry principles have emerged, including hinge-binding heterocycles, optimized hydrogen-bonding interactions, hydrophobic pocket occupancy, conformational control, and strategic scaffold modification. These advances have greatly enhanced our understanding of the structural determinants of c-Met inhibition and continue to guide the design of future inhibitors.

A key lesson from recent discovery programs is that biochemical potency alone cannot predict therapeutic success. Effective c-Met-directed agents must balance target affinity with favorable physicochemical, pharmacokinetic, and cellular properties while retaining activity in biologically relevant tumor models. Similarly, the growing success of dual- and multitarget inhibitors underscores the importance of addressing pathway redundancy and adaptive resistance through rational polypharmacology rather than focusing exclusively on highly selective kinase inhibition.

Despite significant progress, major challenges remain, including acquired resistance driven by kinase-domain mutations, activation of compensatory signaling pathways, tumor heterogeneity, and limited durability of clinical responses. Addressing these obstacles will require continued integration of medicinal chemistry, chemical biology, pharmacology, and precision oncology.

Looking ahead, next-generation c-Met therapeutics are expected to include mutation-directed inhibitors, allosteric and covalent modulators, rational multitarget agents, and targeted protein-degradation technologies. Together with advances in cryo-electron microscopy, artificial intelligence-assisted drug discovery, machine learning-guided SAR analysis, molecular dynamics simulations, and structure-based lead optimization, these emerging approaches are expected to accelerate the development of more effective and durable c-Met-targeted therapies. Continued innovation in these areas will further strengthen the role of c-Met-directed therapeutics in precision oncology and may offer new opportunities to overcome resistance and improve clinical outcomes for cancer patients.

## Figures and Tables

**Table 1:** Representative Type I c-Met inhibitors and their medicinal chemistry characteristics.

Compound	Type	Major Kinase Targets	Clinical Status	Primary Indication	Key Medicinal Chemistry Characteristics
Crizotinib **1**	Ia	c-Met, ALK, ROS1	FDA-approved	NSCLC	First clinically successful c-Met-directed Type Ia inhibitor; multitarget profile
JNJ-38877605 **2**	Ia	c-Met	Phase I (terminated)	Solid tumors	Highly potent but discontinued because of metabolite-induced nephrotoxicity
MK-2461**3**	Ia (non-classical)	c-Met, RON, VEGFR1	Phase I	Solid tumors	Active-state selective ATP-competitive inhibitor
Capmatinib **4**	Ib	c-Met	FDA-approved	c-Met exon 14-skipping NSCLC	Highly selective Type Ib inhibitor with optimized ATP-pocket interactions
Tepotinib **5**	Ib	c-Met	FDA-approved	c-Met exon 14-skipping NSCLC	High selectivity and durable clinical efficacy
Savolitinib **6**	Ib	c-Met	Approved (China)	NSCLC	Selective c-Met inhibitor undergoing expanded clinical development
Vebreltinib **7**	Ib	c-Met	Approved (China)	NSCLC	Optimized oral c-Met inhibitor
Glumetinib **8**	Ib	c-Met	Approved (China)	NSCLC	Improved pharmacokinetic profile
AMG-208 **9**	Ib	c-Met, RON	Phase I/II	Prostate cancer	Dual c-Met/RON inhibitory profile

**Table 2:** Representative Type II c-Met inhibitors and their medicinal chemistry characteristics.

Compound	Major Kinase Targets	Clinical Status	Key Medicinal Chemistry Characteristics	Major Limitations
Cabozantinib **10**	c-Met, VEGFR2, AXL, RET, KIT, FLT3, ROS1	FDA approved	Prototype Type II inhibitor occupying the ATP site and hydrophobic back pocket	Off-target toxicities associated with broad kinase inhibition
Foretinib **11**	c-Met, VEGFR2, RON, AXL, TIE2	Phase II	Quinoline-based scaffold with potent antiangiogenic activity	Pharmacokinetic variability
Golvatinib **12**	c-Met, VEGFR2, Eph receptors	Clinical	Dual kinase inhibitor with anti-invasive activity	Limited clinical efficacy
Merestinib **13**	c-Met, MST1R (RON), AXL, FLT3	Phase II	Broad-spectrum kinase inhibitor	Development discontinued
Glesatinib **14**	c-Met, AXL, VEGFR	Phase II	Active against several c-Met resistance mutations	Clinical efficacy under evaluation
S-49076 **15**	c-Met, AXL, FGFR	Phase I	Targets resistance-associated pathways	Early-stage development
BMS-777607 **16**	c-Met, RON, AXL, TYRO3	Clinical/Preclinical	Broad-spectrum ATP-competitive inhibitor	Limited selectivity
Pamufetinib **17**	c-Met, AXL	Clinical	Optimized for mutation coverage	Limited clinical data
RXDX-106 **18**	TAM kinases, c-Met	Phase I	Combined TAM/c-Met inhibition	Early-stage clinical evaluation

**Table 3:** Comparative medicinal-chemistry trends among synthetic c-Met-directed scaffolds.

Structural Feature or Design Strategy	Representative Scaffolds or Examples	Observed Contribution	Qualifications or Limitation
**Nitrogen-containing hinge-binding heterocycles**	Indoles and indolinones, pyridines, quinolines, pyrimidines, pyrazolo[3,4-*b*]pyridines, pyrrolo[2,3-*d*]pyrimidines, triazolotriazines	Frequently support ATP-pocket recognition and hydrogen bonding with hinge-region residues, commonly Met1160	The interacting nitrogen and binding geometry must be demonstrated for each scaffold; not every nitrogen-rich heterocycle necessarily binds the hinge in the same manner
**Pyridine-containing cores**	Pyridine bioisosteres, 4-phenoxypyridines, thieno[2,3-*b*]pyridines, pyrazolo[3,4-*b*]pyridines	Enable scaffold hopping, hinge recognition, electronic modulation, and attachment of extended pharmacophores	Improved activity depends on substitution pattern and orientation; pyridine incorporation alone does not ensure greater potency or selectivity
**Quinoline-derived extended architectures**	4-Phenoxyquinolines and quinoline-naphthyridine hybrids	Support extended ATP-site and back-pocket occupancy and are compatible with Type II and multikinase inhibitor design	Extended aromatic structures may increase lipophilicity, protein binding, and off-target kinase activity
**Fluorine substitution**	Pyridine, quinoline, pyrazole, thienopyridine, and pyrrolo[2,3-*d*]pyrimidine derivatives	Frequently associated with improved biochemical or cellular activity and may influence hydrophobic contacts, conformation, permeability, and metabolic stability	The benefit was context-dependent; matched nonfluorinated analogs and physicochemical measurements are required to define the mechanism
**Chlorine and trifluoromethyl substitution**	Benzothiazoles, hexahydroquinolines, triazinones, and pyrrolo[2,3-*d*]pyrimidines	Often increased hydrophobic-pocket occupancy and antiproliferative activity	May also increase lipophilicity, protein binding, metabolic persistence, nonspecific binding, and tissue accumulation
**Hydrophobic terminal aromatic groups**	Pyridines, pyrimidines, quinolines, quinazolinone hydrazones, benzothiazoles	Can improve occupancy of lipophilic regions within or adjacent to the ATP-binding pocket	Excessive aromaticity and planarity may compromise solubility, permeability, metabolic behavior, and selectivity
**4-Phenoxy linker architecture**	Pyridine-, quinoline-, pyrimidine-, and dual-target inhibitor series	Provides an extended geometry that can orient hinge-binding and terminal pharmacophores across the ATP site and hydrophobic back pocket	Linker length, substitution, and torsional preferences require optimization; the motif does not establish Type II binding without structural evidence
**Five-atom hydrogen-bond donor/acceptor motif**	Cabozantinib-inspired pyridines, pyrimidines, quinolines, and related Type II designs	Frequently positions polar groups for interactions with residues such as Lys1110 and Asp1222	Its contribution depends on complete molecular geometry; assignment of DFG-out binding requires kinetic or structural confirmation
**1,8-Naphthyridinone pharmacophore**	Quinoline hybrids and pyrrolo[2,3-*d*]pyrimidine series **192** and **199**	Associated with improved cellular activity and, in the optimized series, a more favorable kinase-selectivity profile	The effects of naphthyridinone orientation cannot be separated fully from simultaneous changes elsewhere in the molecules
**Piperazine, morpholine, and related polar cyclic amines**	Pyridines, quinolines, pyrimidines, and selected multitarget inhibitors	May improve aqueous compatibility, modulate basicity, and enhance cellular exposure	These benefits should be confirmed through solubility, pK_a_, permeability, efflux, and pharmacokinetic measurements
**Conformational restriction**	Fused benzothiazoles, thienopyridine-pyridone derivatives, pyrazolopyridines, and triazolotriazines	May preorganize pharmacophores and reduce the entropic cost of binding	Increased rigidity and planarity can also strengthen crystal packing and reduce aqueous solubility
**Scaffold hopping**	Quinoline → pyridine, pyrimidine, thieno[2,3-*b*]pyridine, or pyrrolo[2,3-*d*]pyrimidine	Preserved key pharmacophoric elements while expanding chemical space and, in selected cases, improving potency or selectivity	Comparisons are meaningful only when matched compounds are evaluated under equivalent assay conditions
**Molecular hybridization**	Benzofuran–pyrazoles, quinazolinone hydrazones, indolyl–pyridones, and imidazol-4-ones	Enables integration of complementary pharmacophores and supports dual- or multikinase activity	Larger molecular size and complexity may reduce solubility, permeability, metabolic stability, and mechanistic clarity
**Multitarget pharmacophore integration**	c-Met/VEGFR-2, c-Met/EGFR, c-Met/Pim-1, c-Met/AXL, c-Met/MER, and PARP1/c-Met inhibitors	May suppress pathway redundancy and broaden antitumor activity	Multitarget activity complicates SAR interpretation, biomarker selection, dose optimization, and attribution of efficacy and toxicity
**Docking-guided target assignment**	Thienopyridines **169**, triazinones **177**, and selected indolinone, quinazolinone, and pyrazole derivatives	Generates testable hypotheses regarding ATP-pocket accommodation and possible residue interactions	Should not be presented as proof of direct c-Met inhibition without biochemical, biophysical, or cellular target-engagement evidence

**Table 4:** The reviewed multitarget inhibitors can be broadly categorized according to their biological objectives.

Target Combination	Therapeutic Rationale
**c-Met/VEGFR-2**	Simultaneous suppression of tumor-cell growth and angiogenesis
**c-Met/EGFR**	Prevention of compensatory signaling and resistance in EGFR-driven tumors
**c-Met/CDK2/EGFR**	Combined inhibition of cell-cycle progression and growth-factor signaling
**c-Met/PARP1**	Integration of DNA-repair inhibition with blockade of oncogenic survival signaling
**c-Met/MER**	Simultaneous disruption of tumor growth, immune escape, and metastatic signaling
**c-Met/AXL**	Overcoming resistance and suppressing epithelial–mesenchymal transition (EMT)
**c-Met/Tubulin**	Combining kinase inhibition with disruption of mitotic machinery

## Data Availability

No new data were created or analyzed in this study. Data sharing is not applicable to this article.

## Abbreviations

AKT	Ak strain transforming (often simply called protein kinase B or PKB)
ALK	Anaplastic lymphoma kinase
AR	Androgen receptor
ATP	Adenosine triphosphate
CDK2	Cyclin-dependent kinase 2
c-Kit	Mast/stem cell growth factor receptor
c-Met	Mesenchymal–epithelial transfer factor
CNS	Central nervous system
DIPEA	*N*,*N*-Diisopropylethylamine, Hünig’s base
DMAP	*p*-Dimethylaminopyridine
DMF-DMA	Dimethyl formamide dimethyl acetal
EDCl	1-Ethyl-3-(3-dimethylaminopropyl)carbodiimide
EGFR	Epidermal growth factor receptor
ERK	Extracellular signal-regulated kinase
FDA	Food and Drug Administration
FGFR	Fibroblast growth factor receptor
Flt1	Fms-like tyrosine kinase 1
Flt-3	Fms like tyrosine kinase 3
GDP	Guanosine diphosphate
GTP	Guanosine triphosphate
HATU	Hexafluorophosphate azabenzotriazole tetramethyl uranium
HBTU	Hexafluorophosphate benzotriazole tetramethyl uronium
HGFR	Hepatocyte growth factor receptor
HOAt	1-Hydroxy-7-azabenzotriazole
KDR	Kinase insert domain receptor
*m*-CPBA	*m*-Chloroperbenzoic acid
MEK1	Mitogen-activated protein kinase
Mer	Monocyte epithelial retinal
MST1R	Macrophage-stimulating 1 receptor
MTS	[3-(4,5-Dimethylthiazol-2-yl)-5-(3-carboxymethoxyphenyl)-2-(4-sulfophenyl)-2H-tetrazolium]
MTT	[3-(4,5-Dimethylthiazol-2-yl)-2,5-diphenyl-tetrazolium bromide]
NCI	National cancer institute
NSCLC	Non-small cell lung cancer
PARP1	Poly(ADP-ribose) polymerase 1
PDGFR	Platelet-derived growth factor receptor
PI	Propidium iodide
Pim-1	Proviral integration site for moloney murine leukemia virus-1
PTSA	*p*-Toluenesulfonic acid
RON	Receptive d’origine nantais
ROS1	c-Ros oncogene 1
SAR	Structure–activity relationship
SRB	Sulforhodamine B
TEA	Triethylamine
TGI	Tumor growth inhibition
THF	Tetrahydrofuran
TK	Tyrosine kinase
TYRO3	Tyrosine–protein kinase receptor TYRO3 is an enzyme that in humans is encoded by the *TYRO3* gene
VEGFR	Vascular endothelial growth factor receptor
